# Gut microbiota-metabolome remodeling associated with low bone mass: an integrated multi-omics study in fracture patients

**DOI:** 10.3389/fmolb.2025.1646361

**Published:** 2025-09-01

**Authors:** Xian Zhao, Bin Wu, Pengli Han, Zhongyu Wang, Renwei Cao, Shuo Chen, Cheng Cheng, Hongkai Lian, Yejun Zha, Minjuan Li

**Affiliations:** ^1^ Peking University Fourth School of Clinical Medicine, Beijing, China; ^2^ Department of Endocrinology, Qingdao Municipal Hospital, Qingdao, China; ^3^ Pharmaceutical Department, Zhengzhou Central Hospital Affiliated to Zhengzhou University, Zhengzhou, China; ^4^ Department of Orthopedic Trauma, Beijing Jishuitan Hospital, Capital Medical University, Beijing, China; ^5^ Department of Osteoporosis, Beijing Jishuitan Hospital, Capital Medical University, Beijing, China; ^6^ Institute of Trauma and Metabolism, Zhengzhou Central Hospital Affiliated to Zhengzhou University, Zhengzhou, China; ^7^ Tianjian Laboratory of Advanced Biomedical Sciences, Academy of Medical Sciences, Zhengzhou University, Zhengzhou, China; ^8^ Laboratory for Clinical Medicine, Capital Medical University, Beijing, China

**Keywords:** gut microbiota, metabolomics, bone mineral density, Lachnospira eligens, multiomics integration

## Abstract

**Background:**

The gut microbiota is increasingly implicated in the pathogenesis of osteoporosis, but its role in the specific context of fracture patients remains poorly defined. High-resolution multi-omics studies are needed to elucidate the complex interplay between microbes, their metabolites, and bone health. This study aimed to characterize the gut microbial and fecal metabolic signatures associated with low bone mass in fracture patients.

**Methods:**

We conducted a cross-sectional study of 51 fracture patients, stratified by bone mineral density into Normal, Osteopenia, and Osteoporosis groups. For key analyses, the latter two groups were combined into a Low Bone Mass (LBM) group. We performed shotgun metagenomic sequencing and untargeted liquid chromatography-mass spectrometry metabolomics on fecal samples. An integrated bioinformatics and statistical analysis were used to identify differential taxa and metabolites, construct correlation networks, and build diagnostic biomarker models.

**Results:**

Patients with LBM exhibited a distinct gut microbial and metabolic profile compared to controls. A notable finding was the unexpected enrichment of *Lachnospira eligens* in the LBM group, despite its previous association with gut health. In contrast, traditionally beneficial taxa such as *Bifidobacterium* species and *Bacteroides stercoris* were markedly depleted. Metabolomic analysis identified 127 differential metabolites, and integrated analysis revealed a strong correlation between *L. eligens* and inflammation-associated metabolites, including N-acetylneuraminate. A diagnostic model incorporating four key bacterial species accurately discriminated LBM patients from controls with an area under the curve (AUC) exceeding 0.9.

**Conclusion:**

Our findings reveal a significant remodeling of the gut microbiota-metabolome axis in fracture patients with low bone mass, highlighting a context-dependent, potentially pathological role for the typically beneficial species *L. eligens*. These distinct microbial and metabolic signatures suggest potential mechanistic insights into the gut-bone axis and represent promising, non-invasive biomarkers for assessing skeletal health.

## 1 Introduction

Osteoporosis (OP) is an age-related systemic skeletal disorder characterized by reduced bone mass, microarchitectural deterioration, and increased fracture susceptibility (NIH, 2001; Kanis et al., 1994). It represents a major global health burden, that is escalating with population aging (Johnell and Kanis, 2006; Burge et al., 2007). Current therapies, such as bisphosphonates and monoclonal antibodies, primarily target bone resorption but have limited efficacy in restoring bone quality or addressing the multifactorial etiology of OP(5). The pathogenesis of OP involves complex regulatory networks, including RANKL/OPG and Wnt/β-catenin pathways, as well as systemic modulators like gut-derived serotonin and GLP-1 (Manolagas, 2010; Farr and Khosla, 2019; Yadav et al., 2008). Despite these advances, the precise mechanisms underlying bone metabolic imbalance—particularly the role of extra-skeletal factors like the gut microbiota—are not fully understood (Raisz, 2005).

The gut microbiota, the body’s largest microbial community with a gene pool that vastly exceeds the human genome’s, is vital for host homeostasis (Lindell et al., 2022; Aron-Wisnewsky et al., 2021). A diverse and balanced microbiota is critical for immune regulation, metabolic control, and nutrient absorption (Lynch et al., 2016). Microbial dysbiosis is increasingly implicated in adverse skeletal health effects, contributing to bone loss and the development of OP (Hei et al., 2018; Heme in pathophysiology, 2024). Accumulating evidence supports a “gut-bone axis,” a concept linking microbial dysbiosis to altered bone mineral density (BMD) and fracture risk (Wang et al., 2021; Cheng et al., 2021). Commensal microbes influence bone metabolism via several mechanisms: producing bioactive metabolites (e.g., short-chain fatty acids [SCFAs], bile acids, vitamin K2) that regulate bone cells; modulating immune responses (e.g., the Th17/Treg balance and inflammatory cytokines); and maintaining intestinal barrier integrity (Zaiss et al., 2019; Lucas et al., 2018). For instance, SCFAs can inhibit bone resorption by suppressing NF-κB signaling, while certain bile acids promote osteoblast activity. These findings highlight the therapeutic potential of targeting microbiota-derived metabolites in bone diseases (Ohlsson and Sjögren, 2015; Zaiss et al., 2019), though significant knowledge gaps persist.

These gaps are partly due to methodological limitations in prior research. Many studies have relied on 16S rRNA sequencing, which often lacks the resolution for species-level identification and robust functional prediction that shotgun metagenomic sequencing provides (Hei et al., 2018). Similarly, targeted metabolomics may overlook novel metabolites or pathways relevant to bone health, such as underexplored microbial tryptophan metabolism in osteoporosis (Le Chatelier et al., 2013; Patti et al., 2012). Furthermore, the absence of fracture patients in previous studies has limited clinical translatability. Notably, research has predominantly focused on individuals with established osteoporosis, leaving osteopenia as a relatively understudied condition, especially in the specific context of the gut microbiome and metabolome within fracture patients (Wang et al., 2025). This gap is critical, as it may overlook early microbial or metabolic perturbations that precede severe bone loss and could represent a crucial window for intervention in preventing subsequent fractures (Ticinesi et al., 2025). Therefore, integrative multi-omics approaches are needed. Combining shotgun metagenomics with untargeted metabolomics offers a powerful strategy to decipher the complex microbial and metabolic interactions in bone health (Hei et al., 2018; Franzosa et al., 2018).

We hypothesized that distinct disruptions in gut microbiota–metabolite networks contribute to bone metabolic dysregulation across the spectrum of bone health, including in fracture patients. In this cross-sectional study of individuals with osteoporosis, osteopenia, or normal bone mass, we employed an integrated multi-omics approach (metagenomics and untargeted metabolomics) to: (NIH, 2001): identify microbial and metabolic signatures associated with different bone health states; (Kanis et al., 1994); construct networks linking these features to each other and to relevant metabolic pathways; and (Johnell and Kanis, 2006) identify potential biomarkers that may suggest possible targets for intervention. This strategy helps address previous methodological limitations and provides a more comprehensive framework for understanding the gut-related metabolic alterations in bone mass loss.

## 2 Materials and methods

### 2.1 Study design and ethics statement

This prospective, observational study was conducted at Beijing Jishuitan Hospital. The study was performed in accordance with the principles of the Declaration of Helsinki. The study protocol was reviewed and approved by the ethics committee (Approval No. K2024-273). Written informed consent was obtained from all participants prior to enrollment.

### 2.2 Study population and recruitment

Fragility fractures are often direct clinical manifestations of compromised bone strength and commonly occur in individuals with osteopenia or osteoporosis. Therefore, we selected fracture patients as the study population based on both clinical relevance and biological rationale. This provides a real-world, clinically meaningful context in which to explore the gut–bone axis under conditions of skeletal fragility. We also acknowledge that acute fracture-related factors—such as systemic inflammation, stress responses, and reduced mobility—may influence the gut microbiota composition. To minimize potential confounding effects from these variables, we incorporated the following design measures in our study.

Participants were recruited consecutively between May 2024 and September 2024 from the Department of Orthopedic Trauma at Beijing Jishuitan Hospital. Eligible individuals were inpatients admitted for fracture treatment who met the following criteria. Inclusion criteria: (NIH, 2001): age ≥50 years; (Kanis et al., 1994); radiographically confirmed fracture; (Johnell and Kanis, 2006); stable vital signs and (Burge et al., 2007) the ability to understand the study procedures. Exclusion criteria: (NIH, 2001): pathological fractures secondary to malignancy, infection, or other non-osteoporotic diseases; (Kanis et al., 1994); systemic antibiotic use within 1 month; (Johnell and Kanis, 2006); continuous use of oral or injectable glucocorticoids for more than 3 months within the 6 months; (Burge et al., 2007); presence of diseases known to significantly affect bone metabolism (e.g., hyperparathyroidism, end-stage renal disease, Paget’s disease); and (Raisz, 2005) a history of inflammatory bowel disease or major intestinal surgery.

### 2.3 Bone mineral density assessment and grouping

Following admission, all participants, excluding those with a prior osteoporosis diagnosis who were not receiving regular treatment, underwent Dual-energy X-ray Absorptiometry (DXA) scanning post-admission. Bone mineral density (BMD) was measured at the lumbar spine (L1-L4) and hip (femoral neck or total hip). Participants were classified based on the lowest T-score according to World Health Organization (WHO) criteria: Osteoporosis (T-score ≤−2.5 SD); Osteopenia (−2.5 SD < T-score <−1.0 SD); and Normal bone mass (T-score ≥−1.0 SD, designated as the Control group). For subsequent analyses, the osteoporosis and osteopenia groups were collectively defined as the low bone mass (LBM) group. This combination was based on both clinical and statistical considerations. Clinically, both osteopenia and osteoporosis increase fracture susceptibility. Statistically, combining them into a single LBM group enhanced statistical power to detect significant differences in microbial and metabolic profiles compared to the Control group. This strategy aligns with previous studies in bone health and other disease areas that combine intermediate severity groups to enhance analytical robustness and generalizability, especially when sample sizes for fine-grained stratification are limited (Aleidi et al., 2022).

### 2.4 Fecal sample collection, processing and storage

Fecal samples were collected from each participant prior to any major interventions (e.g., surgery, antibiotic administration) that could potentially affect the gut microbiota. Each sample was then homogenized, and two aliquots were prepared. One aliquot was designated for shotgun metagenomic sequencing and the other for untargeted metabolomics. Samples were immediately stored at −80 °C until further analysis.

### 2.5 Metagenomic sequencing

#### 2.5.1 DNA extraction and library construction

Microbial DNA was extracted from fecal samples using the Fecal Genome DNA Extraction Kit (AU46111-96, BioTeke, China) according to the manufacturer’s instructions. Blank controls, consisting of unused swabs, were processed concurrently with the samples. DNA was quantified using the Qubit 1X dsDNA HS Assay Kit (Invitrogen, Q33230), eluted in 50 µL of buffer, and stored at −80 °C. Subsequent quantification, library preparation, and sequencing were performed by LC-Bio Technology (Hangzhou, China). Paired-end libraries were constructed using the TruSeq Nano DNA LT Library Preparation Kit (FC-121–4001, Illumina). Briefly, approximately 200 ng of DNA per sample was fragmented to a size range of 200–500 bp using a Bioruptor Pico instrument (Diagenode), followed by end repair, A-tailing, adapter ligation, and PCR amplification (8 cycles). The prepared libraries were sequenced on an Illumina NovaSeq 6000 platform using a PE150 (paired-end 150 bp) strategy.

#### 2.5.2 Data preprocessing and assembly

Raw sequencing reads were subjected to quality control using fastp (v0.23.4; parameters: −l 100, -g, -W 6, −5, -q 20, -u 30). The resultant high-quality clean reads were aligned to the human reference genome (GRCh38/hg38) using Bowtie2 (v2.2.0) for the removal of host DNA sequences. Remaining non-host reads were *de novo* assembled on a per-sample basis using MEGAHIT (v1.2.9), and contigs ≥500 bp in length were retained for subsequent analyses.

#### 2.5.3 Gene prediction and annotation

Coding sequences (CDSs) were predicted from the assembled contigs using MetaGeneMark (v3.26); predicted CDSs shorter than 100 nt were discarded. A non-redundant unigene catalog was constructed by clustering all predicted genes using MMseqs2 (v15-6f452; identity ≥95%, coverage ≥90%). Clean non-host reads from each sample were mapped to this unigene catalog using Bowtie2. Unigenes with a total of ≤2 mapped reads across all samples were excluded from further analysis. Taxonomic annotation of unigene protein sequences was performed using DIAMOND (v0.9.14, blastp mode) against the NR_meta database, covering taxonomic ranks from Superkingdom to Species. Functional annotation of unigenes was conducted against the Kyoto Encyclopedia of Genes and Genomes (KEGG) and the Gene Ontology (GO) database (v87.1).

#### 2.5.4 Diversity analysis and biomarker identification

Alpha diversity metrics (Chao1, Observed Species, Shannon, and Simpson indices) were calculated using QIIME1. Beta diversity, based on Bray-Curtis dissimilarity, was visualized using Principal Coordinates Analysis (PCoA). Statistical significance of group differences in overall microbial community structure was assessed by Permutational Multivariate Analysis of Variance (Adonis) using the vegan R package. Differentially abundant taxa between groups were identified using Linear Discriminant Analysis Effect Size (LEfSe), applying a logarithmic LDA score threshold of 2.0. To account for multiple hypothesis testing in the LEfSe analysis, we performed a false discovery rate (FDR) correction on all p-values using the Benjamini–Hochberg procedure. Taxa with q-values <0.05 were considered statistically significant. The results have been included in the supplementary tables (Supplementary Tables S1, S2).

The predictive performance of selected differential taxa as potential biomarkers was evaluated by calculating the Area Under the Receiver Operating Characteristic Curve (AUC). For both binary and multiclass classification tasks (normal, osteopenia, osteoporosis), AUC values and their 95% confidence intervals were estimated using a non-parametric bootstrap resampling approach. In the multiclass setting, bone mass status was treated as an ordinal variable to compute overall AUC values. All proposed biomarker models are exploratory and require further validation in independent cohorts before clinical application. Statistical analyses were performed in R (version 4.5.0; R Foundation for Statistical Computing, Vienna, Austria) and Python (version 3.11.3; Python Software Foundation, Wilmington, DE, United States).

### 2.6 Untargeted metabolomics

#### 2.6.1 Sample preparation and LC-MS acquisition

For metabolite extraction, approximately 20 mg of each fecal sample was homogenized with 1 mL of ice-cold 50% methanol solution, vortex-mixed, incubated at room temperature for 10 min, and then stored overnight at −20 °C to facilitate protein precipitation. In the next day, the samples were thawed and centrifuged at 4,000 × g for 20 min at 4 °C. The resulting supernatants were carefully transferred to a new 96-well plates. Quality control (QC) samples were prepared by pooling equal aliquots from each individual sample extract. All sample extracts were stored at −80 °C until LC-MS analysis.

Chromatographic separation was performed on a Vanquish Flex UHPLC system (Thermo Fisher Scientific, Waltham, MA, United States) coupled with an ACQUITY UPLC BEH C18 column (1.8 µm, 2.1 mm × 100 mm; Waters Corporation, Milford, MA, United States) or a similar reversed-phase column as originally specified (e.g., “ACQUITY UPLC TC column”). The mobile phase consisted of 0.1% formic acid in water (solvent A) and acetonitrile (solvent B). The gradient elution program was as follows: 0–0.5 min, 5% B; 0.5–7.0 min, linear increase from 5% to 100% B; 7.0–8.0 min, 100% B; 8.0–8.1 min, decrease from 100% to 5% B; 8.1–10.0 min, 5% B. Mass spectrometric detection was carried out on an Orbitrap Exploris 480 mass spectrometer (Thermo Fisher Scientific) operating in both positive and negative ionization modes. Full scan MS1 spectra were acquired over an m/z range of 70–1050 with a resolution of 70,000. MS2 spectra were acquired using data-dependent acquisition for the top 3 most intense precursor ions per cycle, with Higher-energy Collisional Dissociation (HCD) and a resolution of 17,500.

#### 2.6.2 Data processing and metabolite identification

Raw LC-MS data files (.raw) were converted to the mzXML format and subsequently processed using the XCMS package in R for non-linear retention time alignment, peak detection, and feature integration. The CAMERA package in R was utilized for the annotation of isotopic peaks, adducts, and in-source fragments. Metabolite identification was achieved by matching accurate mass (mass tolerance <10 ppm) and MS/MS fragmentation patterns against public databases, including the Human Metabolome Database (HMDB) and KEGG. Features with acquired MS2 spectra were additionally matched against an in-house MS2 spectral library. The resulting feature matrix was preprocessed using the metaX package in R, involving the following steps: (NIH, 2001): exclusion of features missing in >50% of QC samples or >80% of biological samples; (Kanis et al., 1994); imputation of remaining missing values using the k-nearest neighbors (KNN) algorithm; and (Johnell and Kanis, 2006) removal of features with a relative standard deviation (RSD) >30% in the QC samples.

#### 2.6.3 Differential metabolite analysis

Principal Component Analysis (PCA) and Partial Least Squares Discriminant Analysis (PLS-DA) were employed to visualize differences in metabolic profiles among the study groups. The robustness and predictive ability of the PLS-DA models were assessed using 200 permutation tests and 7-fold cross-validation. Differential metabolites were identified based on a combination of criteria: a Variable Importance in Projection (VIP) score ≥1.0 from the PLS-DA model, a p-value <0.05 (derived from an appropriate statistical test, e.g., Student’s t-test or Mann-Whitney U test, depending on data distribution), and a fold change (FC) ≥1.2 or ≤0.83 (i.e., 1/1.2). P-values were adjusted for multiple comparisons using the Benjamini–Hochberg false discovery rate (FDR) procedure. KEGG pathway enrichment analysis was performed for the identified differential metabolites to elucidate associated biological pathways.

### 2.7 Integration of metagenomics and metabolomics

Spearman’s rank correlation analysis was performed using R to investigate associations between the relative abundances of differential microbial taxa and the normalized intensities of differential metabolites. Correlation pairs exhibiting an absolute Spearman’s rho (ρ) ≥0.4 and an FDR-adjusted p-value <0.05 were considered significant. These significant correlations were visualized using heatmaps and network diagrams.

### 2.8 General statistical analysis

General statistical analyses for demographic data were performed using R (version 4.5.0; R Foundation for Statistical Computing, Vienna, Austria). Data distribution was assessed for normality using Shapiro-Wilk test. For comparisons between two groups, Student’s t-test or Mann-Whitney U test was applied for continuous variables, respectively. For comparisons among three or more groups, one-way Analysis of Variance (ANOVA) with Tukey’s HSD *post hoc* test or the Kruskal–Wallis test followed by Dunn’s *post hoc* test was used. Categorical variables were compared using the Chi-squared test or Fisher’s exact test. For all analyses, a p-value <0.05 was considered statistically significant.

## 3 Results

### 3.1 Demographic and clinical characteristics of participants

**FIGURE 1:**
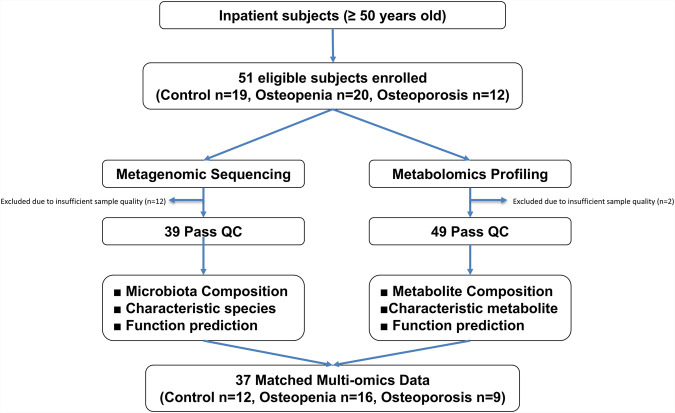
Participant flowchart illustrating sample recruitment, stratification, and reasons for exclusion from omics analyses. A total of 51 fracture patients were initially enrolled and stratified by bone mineral density into Control (n = 19), Osteopenia (n = 20), and Osteoporosis (n = 12) groups. From these participants, 39 participants provided fecal samples suitable for shotgun metagenomic sequencing, while 49 participants’ samples met the quality criteria for untargeted metabolomics profiling. Ultimately, 37 participants provided matched fecal samples that were suitable for both metagenomic and metabolomic analyses.

A total of 51 patients with recent fractures were enrolled and stratified into three groups based on bone mineral density: Control (n = 19), Osteopenia (n = 20), and Osteoporosis (n = 12). Among these, 39 participants underwent metagenomic sequencing, 49 participants underwent untargeted metabolomics profiling, and 37 participants provided matched fecal samples suitable for both analyses. The detailed participant flowchart, including sample distribution for each omics analysis, is presented in Figure 1.

The demographic and clinical characteristics of the participants are summarized in Table 1. The three groups showed a significant difference in age, with Control group being the youngest (59.16 ± 5.26 years) and the Osteoporosis group the oldest (69.17 ± 9.74 years) (*P* = 0.001). No statistically significant differences were observed among the groups regarding sex distribution, body mass index (BMI), lifestyle factors (smoking, alcohol, and tea consumption), prevalence of major comorbidities, or engagement in regular physical exercise. For the multi-omics analyses, matched metagenomic and metabolomic data were available for 12 patients in the Control group, 16 in the Osteopenia group, and 9 in the Osteoporosis group.

**TABLE 1:** Demographic and clinical characteristics among the three patient groups.

Characteristic	Control (n = 19)	Osteopenia (n = 20)	Osteoporosis (n = 12)	*P*
Age, years	59.0 [54.5–62.5]	61.5 [55.8–65.2]	70.6 [63.8–73.2]	0.007
Sex (Female), n (%)	7 (36.8)	14 (70.0)	8 (66.7)	0.089
BMI, kg/m^2^	25.7 [24.0–28.1]	24.4 [22.0–25.8]	24.0 [23.1–25.3]	0.126
Smoking, n (%)	2 (10.5)	1 (5.0)	1 (8.3)	0.827
Alcohol drinking, n (%)	3 (15.8)	1 (5.0)	1 (8.3)	0.622
Tea drinking, n (%)	4 (21.1)	4 (20.0)	1 (8.3)	0.722
Cardiovascular diseases, n (%)	7 (36.8)	5 (15.0)	4 (33.3)	0.741
Metabolic disorders, n (%)	3 (15.8)	3 (15.0)	5 (41.7)	0.191
Physical Exercise, n (%)	4 (21.1)	3 (15.0)	4 (33.3)	0.520
Participated Tests				0.736
-Both Metagenomics and Metabolomics, n (%)	12 (63.2)	16 (80.0)	9 (75.0)	NA
-Only Metagenomics, n (%)	1 (5.3)	1 (5.0)	0 (0.0)	NA
-Only Metabolomics, n (%)	6 (31.6)	3 (15.0)	3 (25.0)	NA

BMI, body mass index; NA, not available. P-values were calculated using Kruskal–Wallis test for continuous variables and Chi-square or Fisher’s exact test for categorical variables.

Note: Cardiovascular diseases include hypertension and coronary heart disease. Metabolic disorders include diabetes mellitus and hyperlipidemia.

### 3.2 Alterations in gut microbiota composition, diversity, and function among the three groups

Sequencing depth was confirmed to be sufficient for capturing the microbial diversity across all samples, as demonstrated by the plateauing rarefaction curves (Supplementary Figure S1A).

**FIGURE 2:**
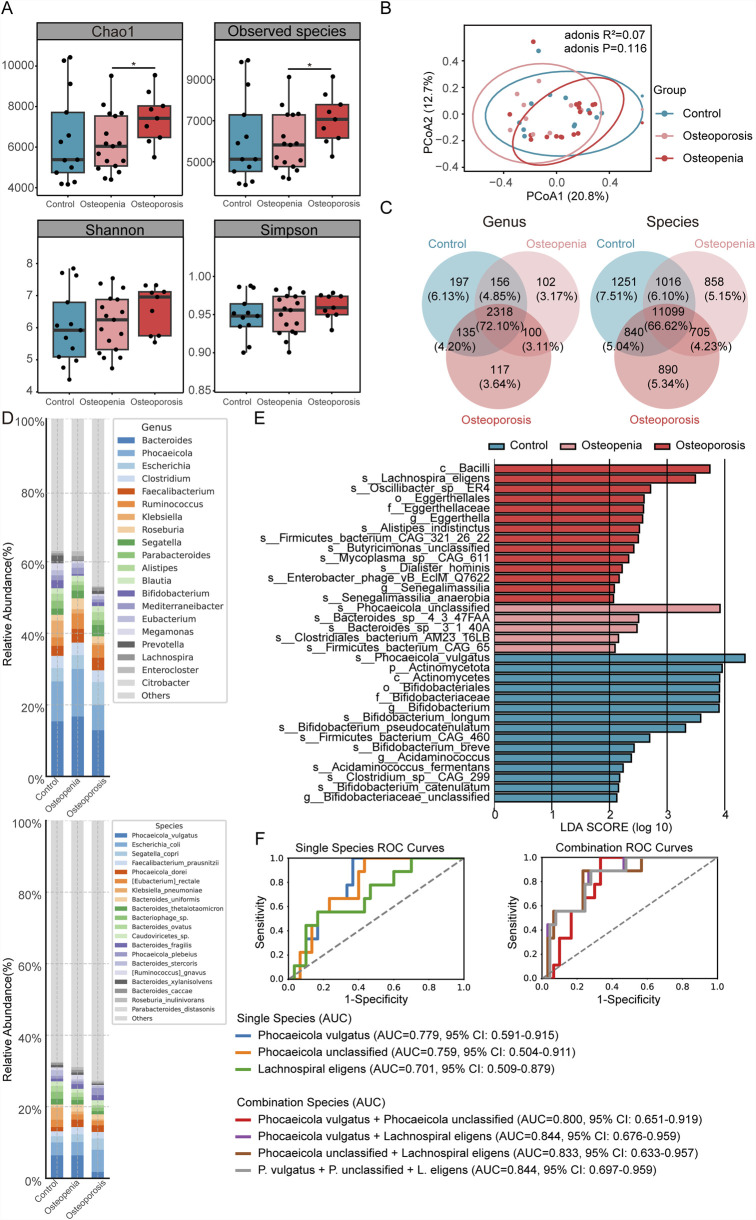
Gut microbiota composition and key taxonomic biomarkers across three bone mass groups. **(A)** Alpha diversity indices (Chao1 and Observed Species) showed increased species richness in the Osteoporosis group compared to Osteopenia. **(B)** Beta diversity via Principal Coordinates Analysis (PCoA) based on Bray-Curtis distances showed no significant separation in overall microbial community structure. **(C)** Venn diagrams illustrate the shared and unique microbial genera and species identified across the groups. **(D)** Stacked bar plots of the dominant genus and species-level taxonomic composition. **(E)** Linear Discriminant Analysis Effect Size (LEfSe) identified 34 significantly differentially abundant taxa (LDA score >2.0); five species were group-specific biomarkers. **(F)** Receiver Operating Characteristic (ROC) curve analysis evaluates the diagnostic potential of three key individual bacterial species (*Phocaeicola vulgatus*, *Phocaeicola unclassified*, *Lachnospira eligens*) and their combination model for classifying the three bone mass states.

Alpha diversity analysis revealed significant differences at the species level. The Osteoporosis group exhibited significantly higher species richness (Chao1 and Observed Species indices) compared to the Osteopenia group (*P* < 0.05, Figure 2A). However, beta diversity analysis using Principal Coordinates Analysis (PCoA) of Bray-Curtis distances showed no significant separation in overall microbial community structure among the three groups, confirmed by Adonis analysis (PERMANOVA, *P* = 0.116, Figure 2B). Venn diagrams illustrated the shared and unique taxa at both genus and species levels across the groups (Figure 2C).

Stacked bar plots provided an overview of the dominant taxonomic composition (Figure 2D). At the genus level, *Bifidobacterium* was significantly enriched in the Control group, while *Lachnospira* was a key biomarker for the Osteoporosis group. Compared to the Control group, the Osteoporosis group displayed substantial shifts in dominant genera, including reduced abundances of *Bacteroides* and *Phocaeicola*. Notably, *Citrobacter* was uniquely elevated in the Osteopenia group, indicating distinct microbiota characteristics for this intermediate bone mass state. Genera such as *Klebsiella*, *Phocaeicola*, *Prevotella*, *Alistipes*, and *Bifidobacterium* also displayed apparent differential trends across the groups, suggesting their potential as distinguishing taxa.

Metagenomic sequencing enabled a more precise analysis at the species level, revealing significant variation in *Phocaeicola vulgatus* across the groups. The Osteoporosis group was characterized by an increased proportion of low-abundance species and greater community dispersion. In the Osteoporosis group, *P. vulgatus* and *Klebsiella pneumoniae* were reduced, while *Escherichia coli* and *Phocaeicola plebeius* were elevated compared to other groups.

To identify specific taxa driving these compositional patterns, we performed Linear Discriminant Analysis Effect Size (LEfSe). This analysis identified 34 significantly differentially abundant taxa (LDA score >2.0), with 22 being at species level (Figure 2E). Applying a more stringent LDA threshold of >3.0, five species were identified as robust group-specific biomarkers: *P. vulgatus*, *Bifidobacterium longum*, and *Bifidobacterium pseudocatenulatum* were significantly enriched in the Control group; an unclassified species of *Phocaeicola* was enriched in the Osteopenia group; and *Lachnospira eligens* was enriched in the Osteoporosis group.

The diagnostic potential of these key species was evaluated using Receiver Operating Characteristic (ROC) curve analysis. Three individual species (*P. vulgatus*, *Phocaeicola unclassified*, *L. eligens*) each yielded an AUC >0.7. A combined model incorporating these three species achieved an AUC of 0.844 (95% CI: 0.697–0.959) for distinguishing between the three bone mass states (Figure 2F). It is important to note that these biomarker models are exploratory and require external validation in independent cohorts due to the study’s modest sample size.

Functional analysis of the metagenomes using KEGG pathway annotation revealed distinct microbial metabolic potentials (Supplementary Figures S1B–E) While no significant differences were observed at KEGG Level 1, the “Replication and repair” pathway at Level 2 was significantly altered. At KEGG Level 3, the pathways for “Lipoarabinomannan (LAM) biosynthesis,” “Chlorocyclohexane and chlorobenzene degradation,” and “DNA replication” were all found to be significantly upregulated in the Osteoporosis group compared to the other groups (Supplementary Figure S1F).

### 3.3 Alterations in gut microbiota composition and diversity between low bone mass and control groups

To identify microbial signatures robustly associated with bone loss, we combined the Osteopenia and Osteoporosis groups into a single Low Bone Mass (LBM) group and compared it against the Control group.

**FIGURE 3:**
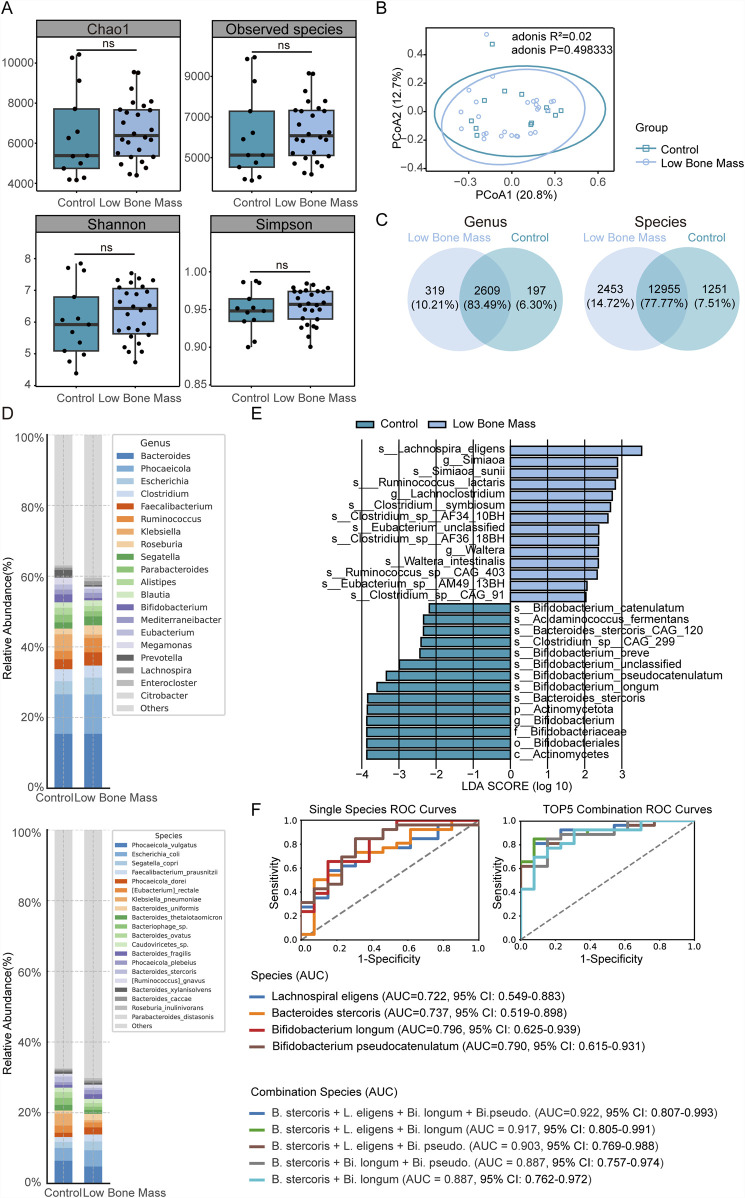
Differential gut microbiota features between Low Bone Mass (LBM) and Control groups. **(A,B)** Alpha and beta diversity metrics show minimal differences between LBM and Control groups. **(C)** Venn diagrams illustrate the shared and unique microbial genera and species. **(D)** Stacked bar plots of dominant genus and species-level taxonomic composition, revealing decreased *Bifidobacterium* and increased *Citrobacter* in LBM. **(E)** LEfSe identified *Lachnospira eligens* as enriched in LBM; *Bacteroides* stercoris and *Bifidobacterium* species enriched in Control. **(F)** ROC curve analysis evaluates the diagnostic potential of four key individual bacterial species and their combination models showed excellent diagnostic performance (AUC = 0.922) for LBM classification.

No significant differences in alpha diversity indices (Chao1, Observed Species, Shannon, Simpson) were found between the LBM and Control groups (Figure 3A). Similarly, the overall community structure did not differ significantly between the two groups (PCoA of Bray-Curtis dissimilarities; PERMANOVA, *R* ^2^ = 0.02, *P* = 0.50, Figure 3B). Venn diagrams illustrated the shared and unique gut microbial genera and species between the LBM and Control groups (Figure 3C).

Stacked bar plots displayed the dominant taxonomic composition (Figure 3D). At the genus level, *Bifidobacterium* was significantly reduced in the LBM group compared to the Control group. The relative abundances of dominant genera such as *Bacteroides* and *Phocaeicola* remained relatively stable across groups, while *Citrobacter* showed a distinct elevation in the LBM group, potentially indicating dysbiosis. Other genera, including *Klebsiella*, *Prevotella*, *Alistipes*, and *Blautia*, exhibited specific variation trends that may serve as potential microbial signatures of low bone mass. At the species level, *Bacteroides uniformis* and *Roseburia inulinivorans* were found to be elevated in the LBM group, whereas *Bacteroides stercoris* was more abundant in the Control group.

LEfSe analysis identified 28 differentially abundant taxa between the LBM and Control groups (LDA score >2.0), including 20 at the species level (Figure 3E). At a stricter LDA threshold of >3.0, four species emerged as robust biomarkers: *L. eligens* was significantly enriched in the LBM group, whereas *B. stercoris*, *B. longum*, and *B. pseudocatenulatum* were all significantly enriched in the Control group.

The diagnostic accuracy of these four species was assessed via ROC curve analysis. In this binary classification analysis, we calculated 95% confidence intervals for all AUC values using the DeLong method. Each species individually achieved an AUC >0.7 in discriminating between the two groups, with *B. longum* yielding the highest individual AUC of 0.796 (95% CI: 0.625–0.939). A combined model incorporating all four species achieved the highest diagnostic performance, with an AUC of 0.922 (95% CI: 0.807–0.993) (Figure 3F). To confirm the superiority of this new biomarker panel, we tested the performance of the previous three-species model (from the three-group analysis) in this binary classification scenario; it yielded a lower AUC of 0.763 (95% CI: 0.636–0.886). This result confirms that the four-species panel is a more powerful and specific biomarker for identifying low bone mass status. Therefore, this four-species signature was used for all subsequent integration analyses. These findings are hypothesis-generating and exploratory, thus requiring external validation in independent cohorts.

### 3.4 Distinct fecal metabolic profiles are associated with low bone mass

**FIGURE 4:**
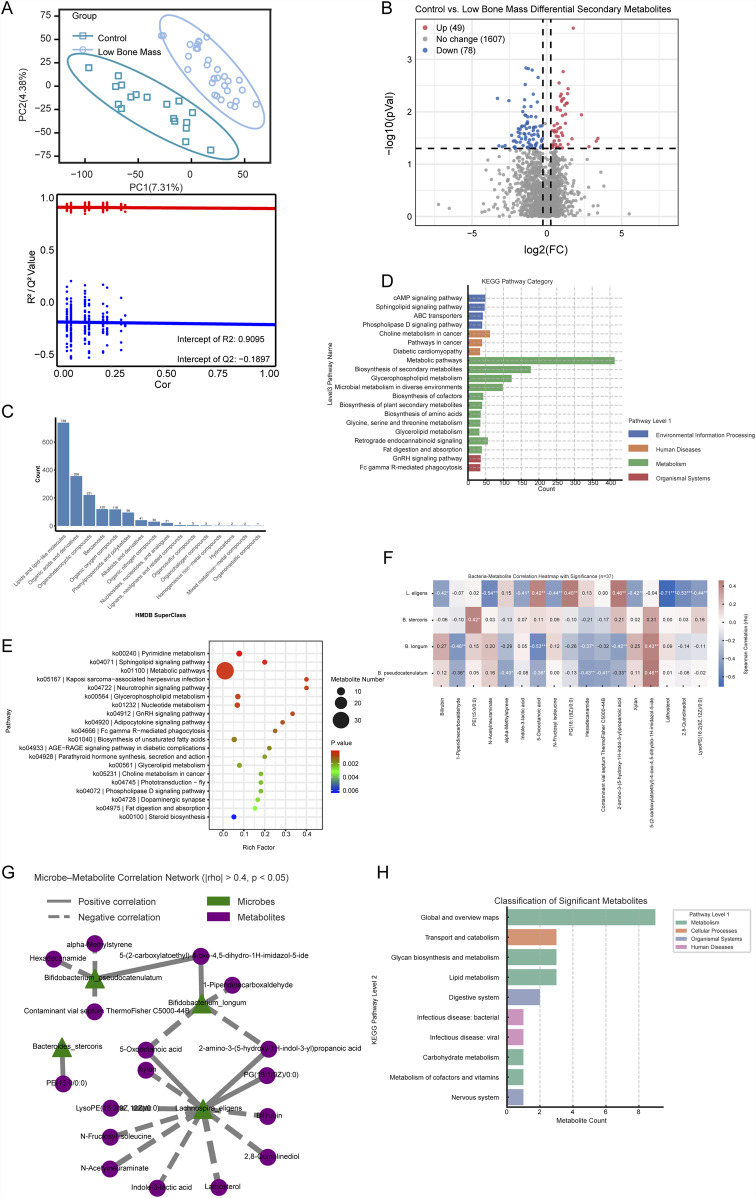
Metabolomic alterations and microbe–metabolite associations in LBM patients. **(A)** PLS-DA score plot showing metabolic separation between LBM and Control. **(B)** Volcano plot of 127 significant differential metabolites. **(C–E)** KEGG classification and enrichment analysis of identified metabolites at multiple pathway levels. **(F)** Heatmap of Spearman correlations between four key bacteria and 17 differential metabolites. **(G)** Network visualization of significant microbe–metabolite interactions. **(H)** KEGG pathway enrichment of 17 microbe-linked metabolites showing involvement in lipid, carbohydrate, and secondary metabolism.

Untargeted metabolomic analysis identified 16,887 metabolic features across all fecal samples in both positive and negative ionization modes. Of these, 13,097 features were from MS1 spectra, and 2,044 features included MS2 spectral data. Among the metabolites with MS2 spectra, 1,060 were annotated against HMDB and 779 against KEGG database, yielding 1,762 classification terms from HMDB and 4,703 from KEGG (Figure 4C). Partial least squares discriminant analysis (PLS-DA) of the metabolomic data revealed a clear separation in fecal metabolic profiles between the Control and LBM groups, indicating significant group-level differences (Figure 4A). We identified 127 differential metabolites (49 upregulated and 78 downregulated in the LBM group) that were confidently annotated with MS2 spectral data (Figure 4B). To understand the biological roles of these differential metabolites, we performed pathway enrichment analysis. At KEGG Level 1, the majority of annotations were categorized under “Metabolism.” At KEGG Level 2, the results showed significant enrichment in several KEGG pathways, including “Metabolic pathways,” “Biosynthesis of secondary metabolites,” and “Glycerophospholipid metabolism” (Figure 4D). Furthermore, significant enrichment of several KEGG Level 3 pathways among the differential metabolites was observed (Figure 4E).

Next, we performed an integration analysis to link these metabolic changes with the key microbial biomarkers identified previously. Spearman correlation analysis was conducted between the four bacterial species and the 127 differential metabolites across the 37 subjects with matched multi-omics data. This revealed 17 metabolites that were significantly correlated (|ρ| > 0.4, *P* < 0.05) with at least one of the key bacteria (Figure 4F). Notably, *L. eligens* (enriched in the LBM group) showed strong positive correlations with N-acetylneuraminate and 5-oxooctanoic acid. These microbe-metabolite interactions are visualized in a correlation network (Figure 4G).

Finally, to elucidate the functional implications of these specific interactions of the 17 correlated metabolites. KEGG pathway analysis showed that these metabolites were predominantly involved in “Carbohydrate metabolism,” “Lipid metabolism,” and “Transport and catabolism” at Level 2, and more specifically in pathways like “Porphyrin metabolism,” “Glycerophospholipid metabolism,” and “Biosynthesis of secondary metabolites” at Level 3, suggesting their roles in lipid processing, energy regulation, and complex host–microbiome metabolic interactions (Figure 4H).

### 3.5 Integrated multi-omics analysis reveals functional links between the microbiome, metabolome, and bone health

**FIGURE 5:**
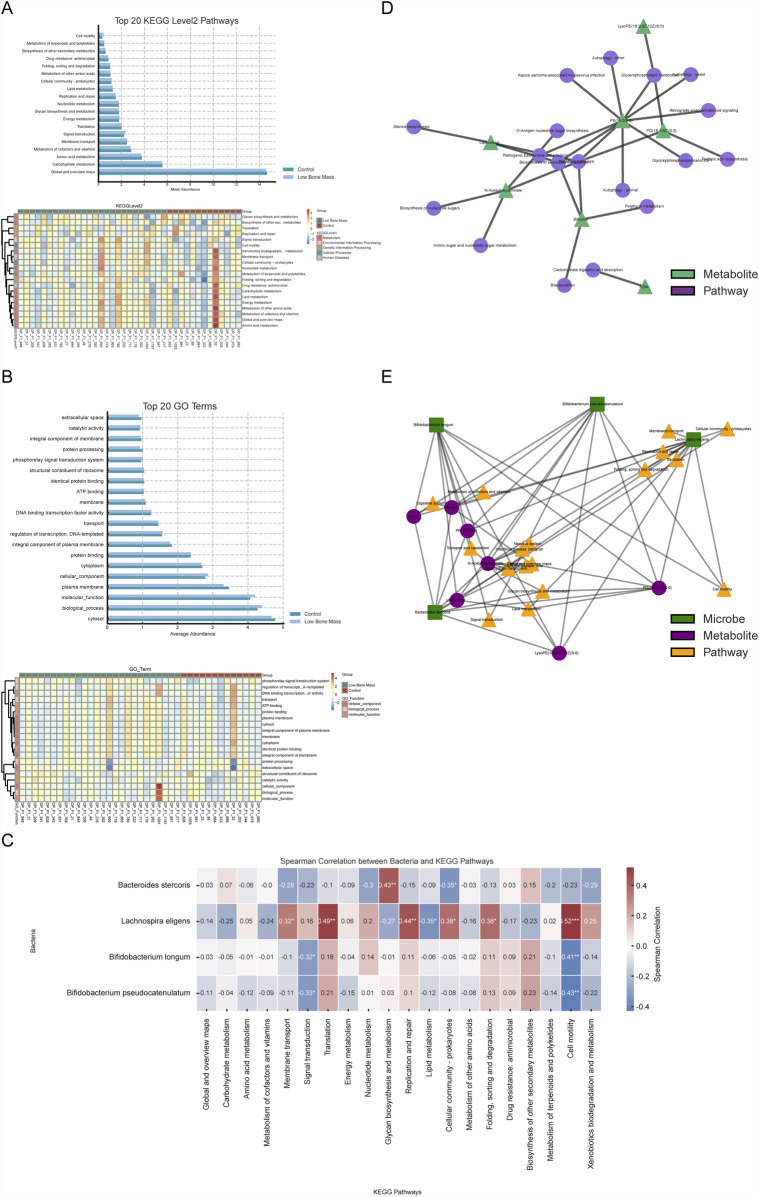
Integrated functional analysis linking microbiota, metabolites, and bone health pathways. **(A, B)** Functional profiles from metagenomic data showing enriched KEGG and GO terms in LBM *versus* Control. **(C)** Correlation matrix linking key bacteria to Level 2 microbial KEGG pathways. **(D)** Metabolite–pathway network highlighting central roles of PG (18:1 (9Z)/0:0) and N-acetylneuraminate. **(E)** Comprehensive multi-omics network connecting species, metabolites, and functional pathways associated with skeletal regulation.

To understand the functional consequences of the altered microbiota in the Low Bone Mass (LBM) group, we first analyzed the functional potential encoded in the metagenomes. At KEGG Level 2, pathways related to signal transduction, metabolism of cofactors and vitamins, and nucleotide metabolism were significantly more abundant in the LBM group (Figure 5A). Conversely, GO analysis revealed that terms associated with baseline cellular activities, such as “cytosol,” “plasma membrane,” and “transcriptional regulation” were more enriched in the Control group, suggesting a broader functional stability in individuals with normal bone mass (Figure 5B).

We investigated the functional impact of the differential fecal metabolites. Pathway enrichment analysis of these metabolites revealed 22 significantly enriched pathways (*Q* < 0.05, Supplementary Figure S2). These included pathways directly relevant to bone physiology, such as the GnRH signaling cascade, parathyroid hormone synthesis, secretion and action, and the AGE-RAGE signaling axis, implying that the gut metabolome may interface with host neuroendocrine and immune circuits that regulate skeletal health.

To investigate direct microbiota-functional pathway interactions, Spearman correlation analysis was conducted between the four key bacterial biomarkers and the top 20 KEGG Level 2 microbial pathways across all 39 samples with metagenomic data (Figure 5C). This analysis revealed distinct functional associations. *Bacteroides stercoris*, enriched in the Control group, was positively correlated with core energy pathways like carbohydrate metabolism. In contrast, *L. eligens*, enriched in the LBM group, showed a negative correlation with lipid metabolism. Additionally, the two *Bifidobacterium* species were positively associated with nutrient biosynthesis pathways, including amino acid and vitamin metabolism.

To elucidate the functional roles of the key microbially-associated metabolites, we focused on the 17 metabolites previously found to be significantly correlated with the four key bacterial species. Among these, seven had clear pathway annotations in the KEGG database. A metabolite-pathway interaction network was constructed for these seven metabolites, revealing their biochemical context (Figure 5D). This network highlighted that hub metabolites, such as PG (18:1 (9Z)/0:0) and N-Acetylneuraminate, were involved in multiple pathways, including “Glycerophospholipid metabolism” and “Amino sugar and nucleotide sugar metabolism,” suggesting their pivotal position in the metabolic dysregulation associated with low bone mass.

Finally, to visualize the complete multi-omics interplay, we constructed an integrated network connecting the four key bacterial species, the seven core metabolites, and their corresponding KEGG Level 2 pathways (Figure 5E). This revealed specific microbe → metabolite → pathway axes. *Lachnospira eligens* was strongly associated with metabolites like Bilirubin and LysoPE (18:2 (9Z,12Z)/0:0), and pathways such as “Membrane transport” and “Translation,” indicating its potential role in regulating host membrane structure and protein synthesis. Similarly, *B. longum* and *B. pseudocatenulatum* were linked to “Lipid metabolism” and “Signal transduction” pathways via their connections with PE and PG class metabolites.

This integrated analysis identified central hubs within the network, including the species *L. eligens* and the metabolites PG (18:1 (9Z)/0:0) and N-Acetylneuraminate, which may serve as critical mediators of the microbiome-metabolome interactions influencing bone mass. These findings reveal a multi-level coupling pattern between gut microbes, metabolites, and functional pathways, offering a new perspective on how the gut ecosystem may synergistically affect skeletal health.

## 4 Discussion

In this study, an integrated multi-omics approach revealed distinct gut microbial and fecal metabolic signatures that differentiate fracture patients with low bone mass (LBM) from those with normal bone mass. These findings provide novel insights into the mechanisms of the “gut-bone axis” in the context of skeletal fragility (Cronin et al., 2022; Valdes et al., 2018; Xu et al., 2017). Our most striking finding was the significant enrichment of *L. eligens* in the LBM group, a finding that appears counterintuitive given its widely recognized commensal role under homeostatic conditions. *L. eligens* belongs to the *Lachnospiraceae* family, which is widely regarded as a cornerstone of a healthy gut ecosystem, largely due to its capacity to produce the short-chain fatty acid butyrate—a metabolite known to suppress osteoclast differentiation and inhibit bone resorption (Chung et al., 2017; Zheng et al., 2025).

Previous studies have consistently portrayed *L. eligens* as health-promoting. It has been shown to be enriched in healthy individuals compared to colorectal cancer patients and has demonstrated anti-inflammatory effects, including the induction of interleukin-10 (IL-10) (Chung et al., 2017; Liu et al., 2021; Kong et al., 2023). Moreover, large-scale cohort studies have associated its abundance with favorable lipid profiles, reinforcing its presumed protective role in metabolic health (Lord et al., 2014). In the context of bone biology, a recent metagenomic study reported a positive correlation between the abundance of multiple *Lachnospiraceae* species and bone mineral density (BMD), which stands in direct contrast to our current findings (Yan et al., 2024). This discrepancy suggests that the role of *L. eligens* in host physiology may be highly context-dependent and potentially strain-specific.

One potential data-driven explanation for this unexpected enrichment involves the strong association observed between *L. eligens* and the metabolite N-acetylneuraminate—a sialic acid involved in immune regulation and bone remodeling (Varki et al., 2009; Shimizu et al., 2015; Varki and Gagneux, 2012). Dysregulation of sialic acid metabolism has been associated with chronic inflammatory conditions, partly through its interaction with sialic acid-binding immunoglobulin-type lectins (Siglecs) on immune cells, which modulate inflammatory signaling and osteoclastogenesis. Emerging evidence suggests that gut microbes may influence host sialic acid pools by producing sialidases that cleave sialic acids from host glycoconjugatesimpacting host-microbe interactions and immune responses (Varki and Gagneux, 2012; Li et al., 2019). Thus, it is plausible that *L. eligens*—especially in the context of systemic inflammation and elevated oxidative stress commonly observed post-fracture—may undergo a functional shift (Wang et al., 2023; Li et al., 2023).While typically a butyrate producer under homeostatic conditions, certain environmental cues, such as inflammatory signals in a compromised host, can trigger metabolic re-programming in commensal bacteria (Sun et al., 2024). In this altered environment, *L. eligens* could instead participate in altered sialic acid metabolism in LBM patients, potentially contributing to low-grade inflammation and bone loss (Noh et al., 2020; Bell et al., 2023).

This hypothesis aligns with prior research identifying *L. eligens* as highly sensitive to oxidative stress (Zünd et al., 2025), suggesting that environmental cues in the post-fracture gut may modulate its gene expression and metabolic output, potentially shifting its role from predominantly butyrogenic to one influencing inflammatory pathways via sialic acid. Furthermore, we found *L. eligens* also significantly associated with 5-oxooctanoic acid, a metabolite with currently undefined physiological relevance in humans, highlighting the need for further exploration.

The enrichment of *L. eligens* in LBM patients may be interpreted through multiple, non-mutually exclusive ecological frameworks. First, its increased abundance may represent a reactive adaptation to a dysbiotic gut environment shaped by fracture-induced stress and systemic inflammation (Vacca et al., 2020; Su et al., 2023). Second, *L. eligens* may be part of a compensatory microbial response attempting to restore host-microbe homeostasis—albeit insufficiently—in the face of host-derived inflammatory signaling (Yang and Cong, 2021). Third, exposure to oxidative or inflammatory cues may trigger functional reprogramming within *L. eligens*, transforming it from a mutualist under healthy conditions into a conditionally adaptive taxon with altered metabolite production (Zeng et al., 2025).

Beyond the observed enrichment of *L. eligens*, our study revealed a broader dysbiosis in the LBM group, characterized by the depletion of several taxa traditionally considered beneficial. Notably, *B. longum* and *B. pseudocatenulatum* were significantly reduced in LBM patients. This is significant, as *Bifidobacterium* species are well-recognized probiotics that can bolster bone health by enhancing mineral absorption, attenuating inflammation, and producing beneficial SCFAs (Collins et al., 2017). Similarly, the depletion of *B. stercoris*, another key SCFA-producing commensal, further points towards an impaired capacity for carbohydrate degradation and gut immune regulation in the LBM group. The cumulative loss of these beneficial microbes—key modulators of host immunity and metabolism via microbial metabolites—may contribute to a gut environment less capable of maintaining skeletal homeostasis and regulating inflammatory tone (Rooks and Garrett, 2016).

This microbial dysbiosis was functionally mirrored by a distinct fecal metabolic signature in LBM patients. Among the 127 differential metabolites identified, pathway enrichment analysis highlighted alterations integral to systemic health. The significant enrichment of pathways like glycerophospholipid metabolism points toward altered cell membrane dynamics and signaling, a finding consistent with recent metabolomic studies that link this pathway to low bone mineral density (Aleidi et al., 2022). Changes in porphyrin metabolism suggest a dysregulated oxidative stress response, as porphyrins (like heme) are central to redox balance and their dysregulation can induce oxidative damage (Heme in pathophysiology). The identification of 17 metabolites that were significantly correlated with our key bacterial biomarkers provides a crucial bridge, confirming that the observed microbial shifts have tangible, functional metabolic consequences.

Ultimately, the strength of our study lies in the integration of these multi-omics datasets to build a cohesive model of the gut-bone axis in fracture patients. Our findings suggest that a compositional shift, marked by the enrichment of a functionally-altered *L. eligens* and the depletion of beneficial *Bifidobacterium* and *Bacteroides* species, drives profound changes in the fecal metabolome. These microbial-derived metabolites then appear to interface with host pathways critical to skeletal health. Our integrated network analysis pinpointed specific, high-impact microbe → metabolite → pathway axes. Particularly noteworthy was the role of phosphatidylglycerol (PG), such as PG (18:1 (9Z)/0:0), which emerged as a critical hub metabolite. Bacterial PGs are known to act as signaling molecules that can directly engage with the host immune system, for instance, by acting as ligands for Toll-like receptors (Choudhary et al., 2019). Its observed role in our network, suggesting a link between the LBM-enriched *L. eligens* and the glycerophospholipid metabolism pathway, may indicate its potential as a key lipid mediator in the gut-bone dialogue. These metabolites and their associated bacteria form a compelling, interconnected network that offers potential mechanistic insights into how the gut ecosystem influences bone mass. However, these proposed functional links are based on associative findings and warrant further experimental validation to establish causality.

The distinct microbial and metabolic signatures identified in this study hold considerable potential for future clinical applications (Hou et al., 2022). Our multi-species microbial models, particularly the four-species panel discriminating LBM from normal bone mass with high accuracy (AUC >0.9), could serve as a basis for developing non-invasive biomarkers. Such tools could help identify individuals at higher risk for low bone mass or stratify fracture patients for personalized interventions.

This study has several strengths, including its integrated multi-omics design and focus on a unique group of fracture patients. However, there are some limitations. First, the cross-sectional design of the study precludes any definitive causal inferences. Second, an age difference was observed between the groups. While this trend accurately reflects the clinical reality that osteoporosis is an age-related disorder, age remains a major confounding factor whose independent effects cannot be fully disentangled from those of bone status itself. Therefore, the observed microbial and metabolic signatures might partially reflect general age-related changes rather than being exclusively linked to low bone mass. This necessitates a cautious interpretation of our cross-sectional associations. Third, the sample size is relatively modest and from a single center, which may limit the generalizability of our findings. We sought to mitigate this by performing a deep, multi-omics characterization for each participant, prioritizing detailed, mechanistic insights over population-level breadth. Fourth, although we collected basic dietary information through a structured questionnaire, this assessment represents a significant limitation given that diet is one of the most critical modulators of gut microbiota composition and function. While no significant differences in dietary habits were observed across groups, we acknowledge that the absence of detailed nutritional profiling may introduce residual confounding. Collectively, these limitations highlight several critical directions for future research. Longitudinal designs or rigorously age-matched cohorts are essential to disentangle the interplay between aging, gut microbiota, and bone status. To minimize dietary confounding, future studies should incorporate precise dietary assessments—such as 24-h recalls or dietary metabolomics—and, ideally, enroll participants with comparable dietary patterns. Finally, establishing causality will require *in vitro* mechanistic studies and prospective clinical interventions to validate the roles of identified microbial species and metabolites in bone metabolism.

## 5 Conclusion

Our integrated multi-omics analysis reveals a substantial remodeling of the gut microbiota-metabolome axis in fracture patients with low bone mass. A notable finding was the enrichment of *L. eligens*, a species generally regarded as beneficial, yet found here to be strongly correlated with inflammation-associated metabolites. This suggests that *L. eligens* may adopt a context-dependent role under pathological conditions such as systemic inflammation and oxidative stress. The findings challenge static classifications of commensal function and underscore the ecological plasticity of the gut microbiota in response to host physiological states. The identified four-species signature, which demonstrated high diagnostic accuracy (AUC >0.9), may serve as a promising non-invasive biomarker and offers novel targets for future diagnostic and therapeutic strategies aimed at skeletal fragility.

## Data Availability

The raw metagenomic sequencing and untargeted metabolomics data generated in this study have been deposited in the Genome Sequence Archive (GSA) under the National Genomics Data Center. The project accession number is CRA027769.
