# Supplementary material for: Gut microbiota-metabolome remodeling associated with low bone mass: an integrated multi-omics study in fracture patients
Source: Front Mol Biosci. 2025 Sep 1;12:1646361. doi: 10.3389/fmolb.2025.1646361 (PMC12433786; doi:10.3389/fmolb.2025.1646361)
Supplement: Supplementary file 1 [file Supplementaryfile1.docx]

***Supplementary Material***

**Supplementary Figures**
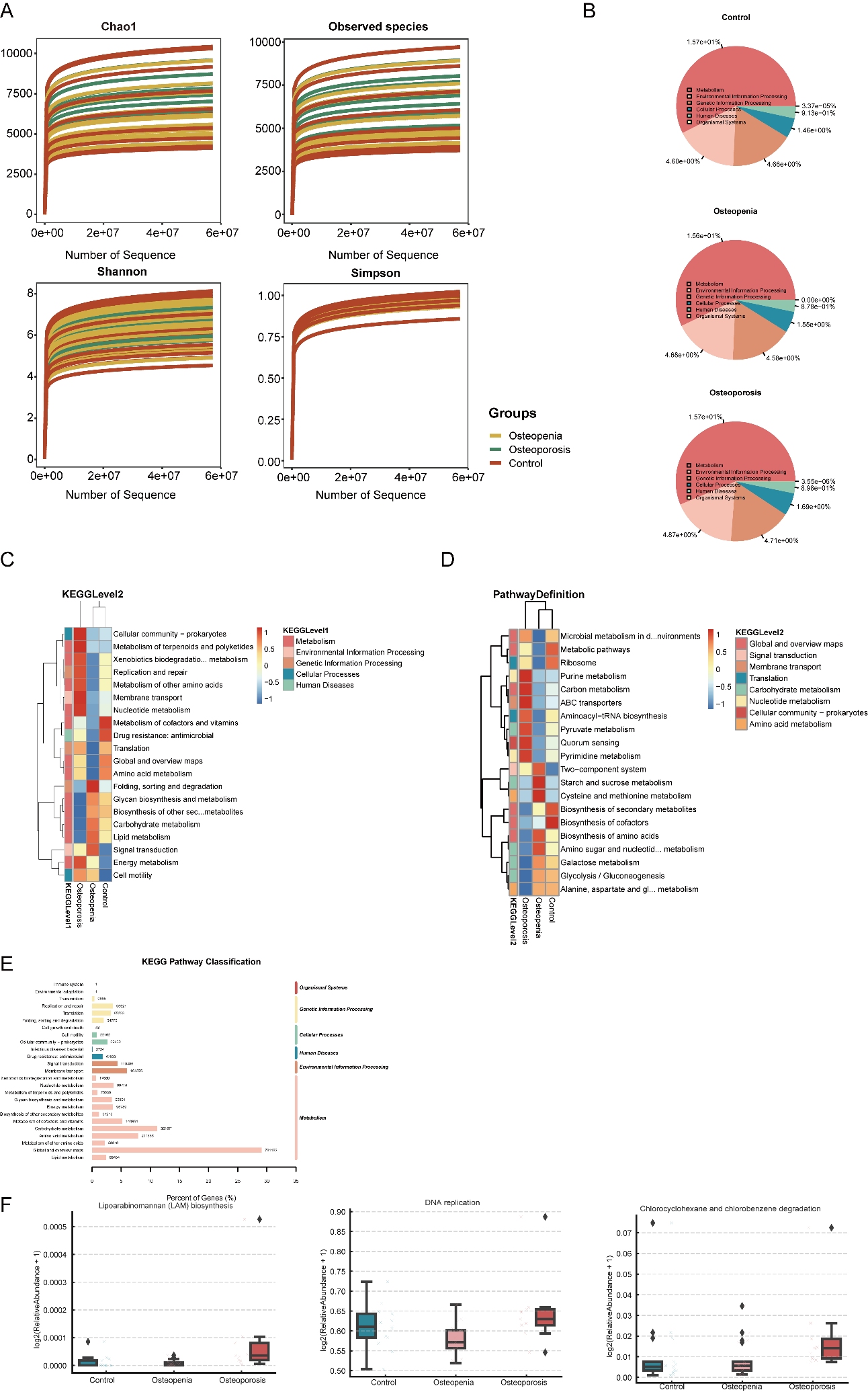


**Supplementary Figure S1.** Microbial diversity and functional potential analysis across bone mass groups. (A) Rarefaction curves based on species richness indices (Chao1, Observed species, Shannon, Simpson) demonstrate adequate sequencing depth for capturing gut microbial diversity across Control, Osteopenia, and Osteoporosis groups. (B) KEGG Level 1 pie charts showing overall distribution of microbial functional categories, with "Metabolism" being dominant across all groups. (C) Heatmap of KEGG Level 2 pathways highlighting differences in metabolic and cellular processing functions among groups. (D) Heatmap of specific KEGG Level 3 pathways (PathwayDefinition) revealing changes in microbial metabolism, translation, and biosynthesis functions. (E) KEGG pathway classification bar chart indicating the percent of annotated genes mapped to major functional categories, categorized by KEGG Level 1. (F) Boxplots showing significantly upregulated KEGG Level 3 pathways in the Osteoporosis group, including “Lipoarabinomannan (LAM) biosynthesis,” “DNA replication,” and “Chlorocyclohexane and chlorobenzene degradation.”


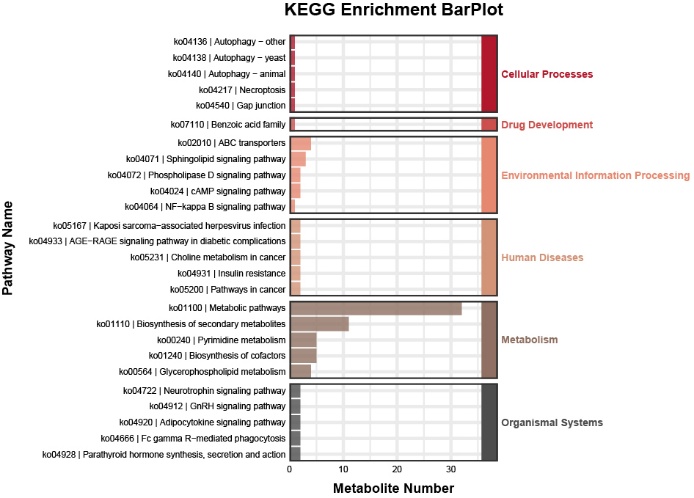


**Supplementary Figure S2.** KEGG pathway enrichment analysis of differential fecal metabolites based on untargeted metabolomics. A total of 22 significantly enriched pathways (Q < 0.05) were identified, including modules related to metabolism, lipid signaling, neuroendocrine regulation, and immune function. Prominent pathways include pyrimidine metabolism, glycerophospholipid metabolism, and AGE-RAGE signaling. These findings suggest that altered fecal metabolite profiles may reflect metabolic reprogramming linked to bone mass status.
