# Supplementary material for: Gut microbiota-metabolome remodeling associated with low bone mass: an integrated multi-omics study in fracture patients
Source: Front Mol Biosci. 2025 Sep 1;12:1646361. doi: 10.3389/fmolb.2025.1646361 (PMC12433786; doi:10.3389/fmolb.2025.1646361)
Supplement: Supplementary file 2 [file Table1.docx]

**Supplementary Table**

Table S1 LEfSe-identified differential taxa among Control, Osteopenia, and Osteoporosis groups with FDR-corrected q-values (Benjamini–Hochberg method)

| **Biomarkernames** | **Log10 Average Abundance** | **Enriched Groups** | **LDA** | **KW_P value** | **q value** |
| --- | --- | --- | --- | --- | --- |
| d__Bacteria.p__Bacteroidota.c__Bacteroidia.o__Bacteroidales.f__Bacteroidaceae.g__Phocaeicola.s__Phocaeicola_vulgatus | 4.807692 | Control | 4.350789 | 0.032036 | 0.047793 |
| d__Bacteria.p__Actinomycetota | 4.439208 | Control | 3.954228 | 0.042609 | 0.047829 |
| d__Bacteria.p__Bacteroidota.c__Bacteroidia.o__Bacteroidales.f__Bacteroidaceae.g__Phocaeicola.s__Phocaeicola_unclassified | 4.389018 | Osteopenia | 3.917224 | 0.044235 | 0.047992 |
| d__Bacteria.p__Actinomycetota.c__Actinomycetes | 4.374344 | Control | 3.910988 | 0.033542 | 0.047793 |
| d__Bacteria.p__Actinomycetota.c__Actinomycetes.o__Bifidobacteriales | 4.359836 | Control | 3.907623 | 0.035386 | 0.047793 |
| d__Bacteria.p__Actinomycetota.c__Actinomycetes.o__Bifidobacteriales.f__Bifidobacteriaceae | 4.359667 | Control | 3.907454 | 0.035386 | 0.047793 |
| d__Bacteria.p__Actinomycetota.c__Actinomycetes.o__Bifidobacteriales.f__Bifidobacteriaceae.g__Bifidobacterium | 4.352468 | Control | 3.900146 | 0.035386 | 0.047793 |
| d__Bacteria.p__Bacillota.c__Bacilli | 4.136257 | Osteoporosis | 3.740763 | 0.044701 | 0.048001 |
| d__Bacteria.p__Actinomycetota.c__Actinomycetes.o__Bifidobacteriales.f__Bifidobacteriaceae.g__Bifidobacterium.s__Bifidobacterium_longum | 3.977527 | Control | 3.582585 | 0.009193 | 0.047793 |
| d__Bacteria.p__Bacillota.c__Clostridia.o__Eubacteriales.f__Lachnospiraceae.g__Lachnospira.s__Lachnospira_eligens | 3.92568 | Osteoporosis | 3.489249 | 0.049285 | 0.049639 |
| d__Bacteria.p__Actinomycetota.c__Actinomycetes.o__Bifidobacteriales.f__Bifidobacteriaceae.g__Bifidobacterium.s__Bifidobacterium_pseudocatenulatum | 3.747261 | Control | 3.318413 | 0.011423 | 0.047793 |
| d__Bacteria.p__Bacillota.c__Clostridia.o__Eubacteriales.f__Oscillospiraceae.g__Oscillibacter.s__Oscillibacter_sp__ER4 | 3.180221 | Osteoporosis | 2.71337 | 0.034024 | 0.047793 |
| d__Bacteria.p__Bacillota.c__Bacillota_unclassified.o__Bacillota_unclassified.f__Bacillota_unclassified.g__Bacillota_unclassified.s__Firmicutes_bacterium_CAG_460 | 2.900706 | Control | 2.697952 | 0.042924 | 0.047928 |
| d__Bacteria.p__Actinomycetota.c__Coriobacteriia.o__Eggerthellales | 3.125803 | Osteoporosis | 2.599623 | 0.011498 | 0.047793 |
| d__Bacteria.p__Actinomycetota.c__Coriobacteriia.o__Eggerthellales.f__Eggerthellaceae | 3.12086 | Osteoporosis | 2.591077 | 0.011498 | 0.047793 |
| d__Bacteria.p__Actinomycetota.c__Coriobacteriia.o__Eggerthellales.f__Eggerthellaceae.g__Eggerthella | 3.014215 | Osteoporosis | 2.575309 | 0.001949 | 0.047793 |
| d__Bacteria.p__Bacteroidota.c__Bacteroidia.o__Bacteroidales.f__Rikenellaceae.g__Alistipes.s__Alistipes_indistinctus | 2.954605 | Osteoporosis | 2.51968 | 0.009163 | 0.047793 |
| d__Bacteria.p__Bacteroidota.c__Bacteroidia.o__Bacteroidales.f__Bacteroidaceae.g__Bacteroides.s__Bacteroides_sp__4_3_47FAA | 2.936289 | Osteopenia | 2.505894 | 0.025714 | 0.047793 |
| d__Bacteria.p__Bacillota.c__Bacillota_unclassified.o__Bacillota_unclassified.f__Bacillota_unclassified.g__Bacillota_unclassified.s__Firmicutes_bacterium_CAG_321_26_22 | 2.92368 | Osteoporosis | 2.500515 | 0.048384 | 0.049404 |
| d__Bacteria.p__Bacteroidota.c__Bacteroidia.o__Bacteroidales.f__Bacteroidaceae.g__Bacteroides.s__Bacteroides_sp__3_1_40A | 2.939218 | Osteopenia | 2.478129 | 0.024659 | 0.047793 |
| d__Bacteria.p__Actinomycetota.c__Actinomycetes.o__Bifidobacteriales.f__Bifidobacteriaceae.g__Bifidobacterium.s__Bifidobacterium_breve | 2.835086 | Control | 2.427641 | 0.017939 | 0.047793 |
| d__Bacteria.p__Bacteroidota.c__Bacteroidia.o__Bacteroidales.f__Odoribacteraceae.g__Butyricimonas.s__Butyricimonas_unclassified | 2.901914 | Osteoporosis | 2.422242 | 0.034762 | 0.047793 |
| d__Bacteria.p__Bacillota.c__Negativicutes.o__Acidaminococcales.f__Acidaminococcaceae.g__Acidaminococcus | 2.612619 | Control | 2.379387 | 0.008506 | 0.047793 |
| d__Bacteria.p__Mycoplasmatota.c__Mollicutes.o__Mycoplasmatales.f__Mycoplasmataceae.g__Mycoplasma.s__Mycoplasma_sp__CAG_611 | 2.668604 | Osteoporosis | 2.332391 | 0.045307 | 0.048164 |
| d__Bacteria.p__Bacillota.c__Negativicutes.o__Acidaminococcales.f__Acidaminococcaceae.g__Acidaminococcus.s__Acidaminococcus_fermentans | 2.437876 | Control | 2.236363 | 0.014339 | 0.047793 |
| d__Bacteria.p__Bacillota.c__Negativicutes.o__Veillonellales.f__Veillonellaceae.g__Dialister.s__Dialister_hominis | 2.482261 | Osteoporosis | 2.219335 | 0.03087 | 0.047793 |
| d__Bacteria.p__Bacillota.c__Clostridia.o__Eubacteriales.f__Clostridiaceae.g__Clostridium.s__Clostridium_sp__CAG_299 | 2.539852 | Control | 2.176398 | 0.013263 | 0.047793 |
| d__Viruses.p__Uroviricota.c__Caudoviricetes.o__Caudoviricetes_unclassified.f__Straboviridae.g__Kanagawavirus.s__Enterobacter_phage_vB_EclM_Q7622 | 2.420668 | Osteoporosis | 2.16784 | 0.032678 | 0.047793 |
| d__Bacteria.p__Bacillota.c__Clostridia.o__Eubacteriales.f__Eubacteriales_unclassified.g__Eubacteriales_unclassified.s__Clostridiales_bacterium_AM23_16LB | 2.678518 | Osteopenia | 2.156228 | 0.01906 | 0.047793 |
| d__Bacteria.p__Actinomycetota.c__Actinomycetes.o__Bifidobacteriales.f__Bifidobacteriaceae.g__Bifidobacterium.s__Bifidobacterium_catenulatum | 2.607304 | Control | 2.156075 | 0.006169 | 0.047793 |
| d__Bacteria.p__Actinomycetota.c__Actinomycetes.o__Bifidobacteriales.f__Bifidobacteriaceae.g__Bifidobacteriaceae_unclassified | 2.565826 | Control | 2.126794 | 0.023134 | 0.047793 |
| d__Bacteria.p__Bacillota.c__Bacillota_unclassified.o__Bacillota_unclassified.f__Bacillota_unclassified.g__Bacillota_unclassified.s__Firmicutes_bacterium_CAG_65 | 2.432628 | Osteopenia | 2.096499 | 0.040336 | 0.047793 |
| d__Bacteria.p__Actinomycetota.c__Coriobacteriia.o__Coriobacteriales.f__Coriobacteriaceae.g__Senegalimassilia | 2.435227 | Osteoporosis | 2.084082 | 0.043044 | 0.047934 |
| d__Bacteria.p__Actinomycetota.c__Coriobacteriia.o__Coriobacteriales.f__Coriobacteriaceae.g__Senegalimassilia.s__Senegalimassilia_anaerobia | 2.409918 | Osteoporosis | 2.070429 | 0.013469 | 0.047793 |

Table S2 LEfSe-identified differential taxa between Control and Low Bone Mass groups with FDR-corrected q-values (Benjamini–Hochberg method)

| **Biomarkernames** | **Log10 Average Abundance** | **Enriched Groups** | **LDA** | **KW_P value** | **q value** |
| --- | --- | --- | --- | --- | --- |
| d__Bacteria.p__Bacteroidota.c__Bacteroidia.o__Bacteroidales.f__Bacteroidaceae.g__Bacteroides.s__Bacteroides_stercoris | 4.301329 | Control | 3.841382 | 0.015815 | 0.048281 |
| d__Bacteria.p__Bacillota.c__Clostridia.o__Eubacteriales.f__Lachnospiraceae.g__Lachnospira.s__Lachnospira_eligens | 3.929619 | Low_Bone_Mass | 3.533706 | 0.025456 | 0.048281 |
| d__Bacteria.p__Actinomycetota.c__Actinomycetes.o__Bifidobacteriales.f__Bifidobacteriaceae.g__Bifidobacterium.s__Bifidobacterium_longum | 4.054272 | Control | 3.589787 | 0.001589 | 0.039888 |
| d__Bacteria.p__Actinomycetota.c__Actinomycetes.o__Bifidobacteriales.f__Bifidobacteriaceae.g__Bifidobacterium.s__Bifidobacterium_pseudocatenulatum | 3.843407 | Control | 3.338938 | 0.003184 | 0.04351 |
| d__Bacteria.p__Actinomycetota.c__Actinomycetes.o__Bifidobacteriales.f__Bifidobacteriaceae.g__Bifidobacterium.s__Bifidobacterium_unclassified | 3.615422 | Control | 2.989064 | 0.029643 | 0.048281 |
| d__Bacteria.p__Bacillota.c__Clostridia.o__Eubacteriales.f__Lachnospiraceae.g__Mediterraneibacter.s___Ruminococcus__lactaris | 3.33945 | Low_Bone_Mass | 2.825214 | 0.049266 | 0.049266 |
| d__Bacteria.p__Bacillota.c__Clostridia.o__Eubacteriales.f__Lachnospiraceae.g__Lachnoclostridium.s___Clostridium__symbiosum | 3.123521 | Low_Bone_Mass | 2.695433 | 0.042778 | 0.048281 |
| d__Bacteria.p__Bacillota.c__Clostridia.o__Eubacteriales.f__Lachnospiraceae.g__Simiaoa.s__Simiaoa_sunii | 3.159418 | Low_Bone_Mass | 2.885005 | 0.037028 | 0.048281 |
| d__Bacteria.p__Bacillota.c__Clostridia.o__Eubacteriales.f__Eubacteriaceae.g__Eubacterium.s__Eubacterium_unclassified | 2.92825 | Low_Bone_Mass | 2.377236 | 0.042778 | 0.048281 |
| d__Bacteria.p__Bacillota.c__Clostridia.o__Eubacteriales.f__Clostridiaceae.g__Clostridium.s__Clostridium_sp__AF34_10BH | 2.978562 | Low_Bone_Mass | 2.62664 | 0.042778 | 0.048281 |
| d__Bacteria.p__Bacillota.c__Clostridia.o__Eubacteriales.f__Oscillospiraceae.g__Ruminococcus.s__Ruminococcus_sp__CAG_403 | 2.719874 | Low_Bone_Mass | 2.337358 | 0.037028 | 0.048281 |
| d__Bacteria.p__Bacillota.c__Clostridia.o__Eubacteriales.f__Clostridiaceae.g__Clostridium.s__Clostridium_sp__AF36_18BH | 2.720272 | Low_Bone_Mass | 2.373896 | 0.037028 | 0.048281 |
| d__Bacteria.p__Actinomycetota.c__Actinomycetes.o__Bifidobacteriales.f__Bifidobacteriaceae.g__Bifidobacterium.s__Bifidobacterium_breve | 2.894815 | Control | 2.436193 | 0.005594 | 0.048281 |
| d__Bacteria.p__Actinomycetota.c__Actinomycetes.o__Bifidobacteriales.f__Bifidobacteriaceae.g__Bifidobacterium.s__Bifidobacterium_catenulatum | 2.700076 | Control | 2.182928 | 0.001946 | 0.039888 |
| d__Bacteria.p__Bacillota.c__Clostridia.o__Eubacteriales.f__Lachnospiraceae.g__Waltera.s__Waltera_intestinalis | 2.643653 | Low_Bone_Mass | 2.366903 | 0.025456 | 0.048281 |
| d__Bacteria.p__Bacteroidota.c__Bacteroidia.o__Bacteroidales.f__Bacteroidaceae.g__Bacteroides.s__Bacteroides_stercoris_CAG_120 | 2.697006 | Control | 2.338504 | 0.044318 | 0.048281 |
| d__Bacteria.p__Bacillota.c__Clostridia.o__Eubacteriales.f__Eubacteriaceae.g__Eubacterium.s__Eubacterium_sp__AM49_13BH | 2.433135 | Low_Bone_Mass | 2.061724 | 0.045926 | 0.048281 |
| d__Bacteria.p__Bacillota.c__Clostridia.o__Eubacteriales.f__Clostridiaceae.g__Clostridium.s__Clostridium_sp__CAG_299 | 2.640142 | Control | 2.404085 | 0.027481 | 0.048281 |
| d__Bacteria.p__Bacillota.c__Clostridia.o__Eubacteriales.f__Clostridiaceae.g__Clostridium.s__Clostridium_sp__CAG_91 | 2.348845 | Low_Bone_Mass | 2.032262 | 0.025448 | 0.048281 |
| d__Bacteria.p__Bacillota.c__Negativicutes.o__Acidaminococcales.f__Acidaminococcaceae.g__Acidaminococcus.s__Acidaminococcus_fermentans | 2.595294 | Control | 2.33685 | 0.049266 | 0.049266 |
| d__Bacteria.p__Actinomycetota | 4.487644 | Control | 3.851401 | 0.037028 | 0.048281 |
| d__Bacteria.p__Actinomycetota.c__Actinomycetes | 4.437719 | Control | 3.865606 | 0.017155 | 0.048281 |
| d__Bacteria.p__Actinomycetota.c__Actinomycetes.o__Bifidobacteriales | 4.427971 | Control | 3.864192 | 0.014567 | 0.048281 |
| d__Bacteria.p__Actinomycetota.c__Actinomycetes.o__Bifidobacteriales.f__Bifidobacteriaceae | 4.427971 | Control | 3.864192 | 0.014567 | 0.048281 |
| d__Bacteria.p__Actinomycetota.c__Actinomycetes.o__Bifidobacteriales.f__Bifidobacteriaceae.g__Bifidobacterium | 4.427971 | Control | 3.864192 | 0.014567 | 0.048281 |
| d__Bacteria.p__Bacillota.c__Clostridia.o__Eubacteriales.f__Lachnospiraceae.g__Lachnoclostridium | 3.249067 | Low_Bone_Mass | 2.742345 | 0.039815 | 0.048281 |
| d__Bacteria.p__Bacillota.c__Clostridia.o__Eubacteriales.f__Lachnospiraceae.g__Simiaoa | 3.159418 | Low_Bone_Mass | 2.885005 | 0.037028 | 0.048281 |
| d__Bacteria.p__Bacillota.c__Clostridia.o__Eubacteriales.f__Lachnospiraceae.g__Waltera | 2.643653 | Low_Bone_Mass | 2.366903 | 0.025456 | 0.048281 |

Table S3 Detailed information on 127 differential metabolites identified between bone mass groups.

| Type | ID | MS2.name | Formula | SuperClass | Class | Subclass | HMDB | kegg | kegg_pathway | MS1.name | FC | regulation | p_value | q_value | VIP |
| --- | --- | --- | --- | --- | --- | --- | --- | --- | --- | --- | --- | --- | --- | --- | --- |
| NEG | NEG_191.056_0.6142_1 | Quinic acid | C7H12O6 | Organic oxygen compounds | Organooxygen compounds | Alcohols and polyols | HMDB0003072 | C00296 | map00400(Phenylalanine, tyrosine and tryptophan biosynthesis);map01100(Metabolic pathways) | 6,8-Bis(sulfanyl)octanal;Isopropyl tartaric acid;2-Hydroxypyrazine;3(2H)-Pyridazinone;5-Hydroxypyrimidine;pyrimidin-2-ol;pyrimidin-4-ol;2-Methylglutaric acid;Adipic acid;Methylglutaric acid;Monomethyl glutaric acid;2,2-Dimethylsuccinic acid;Solerol;(S)-2-Aceto-2-hydroxybutanoic acid;Dimethyl succinate;4-Ethoxy-4-oxobutanoic acid;1,2,4-Benzotriazin-3-amine;2-Ethylbutanedioic acid;2,3-Dimethylsuccinic acid;(5S)-5,6-Dihydroxy-2-oxohexanal;Ethylidene diacetate;Acetone lactate;Conduritol B;Dianhydrodulcitol;Glucal;isosorbide;3-(Hydroxymethyl)-5-oxopentanoic acid;2-dehydropantoate;Monoethyl malonic acid;Ethylmalonic acid;Glutaric acid;Methylsuccinic acid;Dimethylmalonic acid;2-Acetolactate;2-C-Methyl-1,4-erythrono-D-lactone;2-Deoxy-L-ribono-1,4-lactone;2-Hydroxy-4-oxopentanoic acid;Monomethyl succinate;Dimethyl malonate;Pteridine;Pyrimido[5,4-d]pyrimidine;Formyl 3-hydroxybutanoate | 0.23 | down | 0.05 | 0.57 | 1.39 |
| POS | POS_316.248_3.9241_1 | Decanoylcarnitine | C17H33NO4 | Lipids and lipid-like molecules | Fatty Acyls | Fatty acid esters | HMDB0000651 | C03299 | NA | Decanoylcarnitine;6-Methylnonanoylcarnitine;5-Methylnonanoylcarnitine;8-Methylnonanoylcarnitine;7-Methylnonanoylcarnitine;4-Methylnonanoylcarnitine;3-Methylnonanoylcarnitine;N-Myristoyl Serine | 0.74 | down | 0.05 | 0.57 | 1.24 |
| POS | POS_279.134_2.7154_1 | Tyr-Pro | C14H18N2O4 | Organic acids and derivatives | Carboxylic acids and derivatives | Amino acids, peptides, and analogues | NA | NA | NA | 3,4-Dihydroxybenzylamine;5-Acetyl-2,4-dimethyloxazole;Ethyl 2-pyrrolecarboxylate;3-((Aminooxy)methyl)phenol;3-(1H-Pyrrol-2-yl)propanoic Acid;Deferiprone;Dihydroxybenzylamine;Dimethyl-3-hydroxypyrid-4-one;3-Amino-2,3-dihydrobenzoic acid;N1-(alpha-D-ribosyl)-5,6-dimethyl-benzimidazole;Prolyl-Tyrosine;Tyrosyl-Proline;4'-Hydroxymethohexital;Musk moskene;L-Menthyl acetoacetate;Falcarindione;4,4'-Methylenebis(2,6-dimethylphenol) | 0.35 | down | 0.03 | 0.56 | 1.66 |
| POS | POS_153.099_1.221_1 | N&#937;-Acetylhistamine | C7H11N3O | Organic acids and derivatives | Carboxylic acids and derivatives | Carboxylic acid derivatives | NA | C05135 | map00340(Histidine metabolism) | N-Acetylputrescine;(S)-2-Amino-4-methylpentanamide;2,6-Diaminohexanal;L-isoleucinamide;N-(2-Hydroxyethyl)piperazine;N-Amyl-N-methylnitrosamine;N-Nitrosodipropylamine;N-Nitrosomethylamylamine | 2.06 | up | 0.05 | 0.57 | 1.53 |
| POS | POS_127.05_2.6444_1 | Thymine | C5H6N2O2 | Organoheterocyclic compounds | Diazines | Pyrimidines and pyrimidine derivatives | HMDB0000262 | C00178 | map00240(Pyrimidine metabolism);map01100(Metabolic pathways);map01232(Nucleotide metabolism) | Thymine;Imidazoleacetic acid;1H-Imidazole-1-acetic acid;5-Methyl-1H-pyrazole-3-carboxylic acid;6-Methyluracil | 0.52 | down | 0.05 | 0.57 | 1.05 |
| NEG | NEG_203.083_2.7325_1 | 3,4-Dihydroxybenzeneacetic acid | C8H8O4 | Benzenoids | Phenols | Benzenediols | HMDB0001336 | C01161 | map00350(Tyrosine metabolism);map00946(Degradation of flavonoids);map01100(Metabolic pathways);map01120(Microbial metabolism in diverse environments);map01220(Degradation of aromatic compounds);map04728(Dopaminergic synapse);map05030(Cocaine addiction);map05031(Amphetamine addiction);map05034(Alcoholism) | L-Tryptophan;D-Tryptophan;3-Hydroxymethylantipyrine;Ethotoin;(&#177;)-Tryptophan;Nirvanol;4-Hydroxyantipyrine;S-nirvanol;3h-Indole-3-propanoic acid,a-amino-;Idazoxan;trans-4,5-epoxy-2(E)-decenal;Ethyl (4Z)-4,7-octadienoate;Methyl octynecarboxylate;(+/-)-Dihydromintlactone;2,4-Hexadienyl butyrate;2,4-Hexadienyl isobutyrate;cis-3-Hexenyl crotonate;(+/-)-2-(5-Methyl-5-vinyltetrahydrofuran-2-yl)propionaldehyde;(-)-(Z)-Tetrahydro-6-(2-pentenyl)-2H-pyran-2-one;5,6-Dihydro-6-pentyl-2H-pyran-2-one;6-Hydroxy-2,6-dimethyl-2,7-octadien-4-one;Epoxyartemisia ketone;Ascaridole;Dihydronepetalactone;Epoxycampholenic aldehyde;Geranic acid;gamma-Diosphenol;4-Isopropyl-3-cyclohexene-1-carboxylic acid;xi-1,8,8-Trimethyl-2-oxabicyclo[3.2.1]octan-3-one;(xi)-(Z)-5-(3-Hexenyl)dihydro-2(3H)-furanone;6-exo-Hydroxyfenchone;6-endo-Hydroxyfenchone;Sebacil;4,5-Epoxydec-2(trans)-enal;Dihydroperillic acid;TriMM;(+)-Bottrospicatol;(Z)-3,5-Hexadienyl butyrate;Neric acid;Piperitone oxide;(+/-)-2-Hydroxypiperitone;(&#172;&#177;)-Limonene diepoxide;p-mentha-1,8-dien-4-yl-hydroperoxide;(E)-p-mentha-1(7),8-dien-2-hydroperoxide;(E)-p-mentha-6,8-dien-2-hydroperoxide;(Z)-p-mentha-1,8-dien-2-hydroperoxide;(3S)-3-hydroxycyclocitral;1-Benzylimidazole;1,2-Diaminonaphthalene;1,5-Naphthalenediamine;2,3-Diaminonaphthalene;Nicotyrine;5-Methylquinoxaline;2-Aminoquinoline;1-Phenylpyrazole;1,4-Benzodiazepine;1H-1,5-Benzodiazepine;2-Phenylimidazole;3-Phenyl-1H-pyrazole;3H-2,3-Benzodiazepine;4-Aminoquinoline;5-Aminoisoquinoline;6-Aminoquinoline;8-Aminoquinoline;Benzodiazepine;Dipyrrin;2-methyl chinoxaline | 0.36 | down | 0.03 | 0.56 | 1.89 |
| NEG | NEG_136.049_3.1953_1 | Anthranilic acid | C7H7NO2 | Benzenoids | Benzene and substituted derivatives | Benzoic acids and derivatives | NA | C00108 | map00380(Tryptophan metabolism);map00400(Phenylalanine, tyrosine and tryptophan biosynthesis);map00405(Phenazine biosynthesis);map00621(Dioxin degradation);map00627(Aminobenzoate degradation);map00996(Biosynthesis of various alkaloids);map00997(Biosynthesis of various other secondary metabolites);map00998(Biosynthesis of various antibiotics);map00999(Biosynthesis of various plant secondary metabolites);map01060(Biosynthesis of plant secondary metabolites);map01061(Biosynthesis of phenylpropanoids);map01063(Biosynthesis of alkaloids derived from shikimate pathway);map01070(Biosynthesis of plant hormones);map01100(Metabolic pathways);map01110(Biosynthesis of secondary metabolites);map01120(Microbial metabolism in diverse environments);map01220(Degradation of aromatic compounds);map01230(Biosynthesis of amino acids);map07110(Benzoic acid family) | NA | 1.87 | up | 0.01 | 0.52 | 1.96 |
| POS | POS_229.154_2.7996_1 | Leu-Pro | C11H20N2O3 | Organic acids and derivatives | Carboxylic acids and derivatives | Amino acids, peptides, and analogues | HMDB0011175 | NA | NA | Isoleucylproline;Leucylproline;N,N,N-Trimethyl-L-alanyl-L-proline betaine;Pro-leu;Pro-Ile;4-(1,1,3,3-Tetramethylbutyl)-phenol;delta-Methylionone;3-Methyl-4-(2,6,6-trimethyl-2-cyclohexen-1-yl)-3-buten-2-one;Methyl-delta-ionone;1-(2,6,6-Trimethyl-2-cyclohexen-1-yl)-1-penten-3-one;alpha-Irone;Etaspirene;10-Isopropyl-2,7-dimethyl-1-oxaspiro[4.5]deca-3,6-diene;1-(2,6,6-Trimethyl-1-cyclohexen-1-yl)-1-penten-3-one;(Z)-alpha-Irone;4-(1,1-Dimethylhexyl)phenol;4-Octylphenol;4-(2,5,6,6-Tetramethylcyclohexen-1-yl)but-3-en-2-one;Octylphenol;Phenol, 2-(1,1,3,3-tetramethylbutyl)-;Cedranoxide;Zerumbodienone;Methoxamine;Orciprenaline;Isoproterenol;mescaline | 0.38 | down | 0.01 | 0.53 | 1.97 |
| NEG | NEG_583.257_6.7161_1 | Bilirubin | C33H36N4O6 | Organoheterocyclic compounds | Tetrapyrroles and derivatives | Bilirubins | HMDB0000054 | C00486 | map00860(Porphyrin metabolism);map01100(Metabolic pathways);map01110(Biosynthesis of secondary metabolites);map04976(Bile secretion) | Bilirubin;(4E,15Z)-Bilirubin;(4E,15E)-Bilirubin;Lumirubin;11,12-Dimethoxydihydrokawain;Coriandrone A;Coriandrone B;Mono-(2-ethyl-5-oxohexyl) phthalate;Cladosporin;3-(2-Ethyl-5-oxohexyl)phthalic acid;11b,13-Dihydrolactucin | 0.65 | down | 0.02 | 0.56 | 1.59 |
| POS | POS_144.081_4.7483_1 | Tryptamine | C10H12N2 | Organoheterocyclic compounds | Indoles and derivatives | Tryptamines and derivatives | HMDB0000303 | C00398 | map00380(Tryptophan metabolism);map00901(Indole alkaloid biosynthesis);map01060(Biosynthesis of plant secondary metabolites);map01063(Biosynthesis of alkaloids derived from shikimate pathway);map01100(Metabolic pathways);map01110(Biosynthesis of secondary metabolites);map04080(Neuroactive ligand-receptor interaction) | 6-Methylquinoline;2-Aminonaphthalene;Quinaldine;1-Naphthylamine;1-Benzazepine;8-Methylquinoline | 2.56 | up | 0.01 | 0.53 | 2.27 |
| NEG | NEG_297.244_6.4041_1 | Ricinoleic acid | C18H34O3 | Lipids and lipid-like molecules | Fatty Acyls | Fatty acids and conjugates | HMDB0034297 | C08365 | NA | 3-Oxooctadecanoic acid;9-Oxooctadecanoic acid;10-Oxooctadecanoic acid;11-Oxooctadecanoic acid;5-Hexyltetrahydro-2-furanoctanoic acid;5-Oxooctadecanoic acid;Ricinoleic acid;(12R)-12-Hydroxyoctadec-9-enoic acid;9,10-Epoxystearic acid;Methyl palmoxirate;13-Heptadecyn-1-ol;8-Heptadecenal;3-Methylcyclopentadecanone;(&#177;)-2-Dodecylcyclobutanone;Hexadecenal;(Z)-11-Hexadecenal;Hexadec-2-enal;9-Hexadecenal, (9Z)- | 2.02 | up | 0.01 | 0.52 | 1.61 |
| NEG | NEG_478.295_4.6062_1 | PE(18:1(9Z)/0:0) | C23H46NO7P | Lipids and lipid-like molecules | Glycerophospholipids | Glycerophosphoethanolamines | NA | C04438 | map00564(Glycerophospholipid metabolism) | LysoPE(0:0/18:1(11Z));LysoPE(0:0/18:1(9Z));LysoPE(18:1(11Z)/0:0);LysoPE(18:1(9Z)/0:0);Mofarotene | 2.68 | up | 0.04 | 0.57 | 1.23 |
| POS | POS_152.071_2.8472_1 | cis-1,2,3,6-Tetrahydrophthalimide | C8H9NO2 | Organoheterocyclic compounds | Isoindoles and derivatives | Isoindolines | NA | NA | NA | Acetaminophen;2-Phenylglycine;Dopamine quinone;Leukoaminochrome;Methyl 2-aminobenzoate;2-(Methylamino)benzoic acid;3alpha,4,7,7alpha-Tetrahydro-1H-isoindole-1,3(2H)-dione;Ethyl nicotinate;2-Amino-3-methylbenzoate;2'-Hydroxyacetanilide;2-Ethylnitrobenzene;Metacetamol;3-Methoxybenzamide;3,4,5,6-Tetrahydrophthalimide;4-(Aminomethyl)benzoic acid;4-Aminophenylacetic acid;4-Methyl-1,4-dihydropyridine-3,5-dicarbaldehyde;N-Phenylglycine;1-nitro-2-phenylethane;(Z)-[(4-hydroxyphenyl)acetaldehyde oxime];Phthalide;Phenylglyoxal;2,2'-Bifuran;o-Phthalaldehyde;Phenol-formaldehyde resin | 10.64 | up | 0.03 | 0.56 | 1.22 |
| NEG | NEG_329.24_5.7098_1 | 7Z, 10Z, 13Z, 16Z, 19Z-docosapentaenoic acid | C22H34O2 | Lipids and lipid-like molecules | Fatty Acyls | Fatty acids and conjugates | NA | C16513 | map01040(Biosynthesis of unsaturated fatty acids) | NA | 0.23 | down | 0.04 | 0.56 | 1.70 |
| POS | POS_597.45_9.0839_1 | DG 33:4 | C36H62O5 | Lipids and lipid-like molecules | Glycerolipids | Diradylglycerols | NA | C00165 | map01521(EGFR tyrosine kinase inhibitor resistance);map04010(MAPK signaling pathway);map04012(ErbB signaling pathway);map04014(Ras signaling pathway);map04015(Rap1 signaling pathway);map04020(Calcium signaling pathway);map04024(cAMP signaling pathway);map04062(Chemokine signaling pathway);map04064(NF-kappa B signaling pathway);map04066(HIF-1 signaling pathway);map04071(Sphingolipid signaling pathway);map04072(Phospholipase D signaling pathway);map04261(Adrenergic signaling in cardiomyocytes);map04270(Vascular smooth muscle contraction);map04361(Axon regeneration);map04370(VEGF signaling pathway);map04371(Apelin signaling pathway);map04540(Gap junction);map04611(Platelet activation);map04613(Neutrophil extracellular trap formation);map04625(C-type lectin receptor signaling pathway);map04650(Natural killer cell mediated cytotoxicity);map04658(Th1 and Th2 cell differentiation);map04659(Th17 cell differentiation);map04660(T cell receptor signaling pathway);map04662(B cell receptor signaling pathway);map04664(Fc epsilon RI signaling pathway);map04666(Fc gamma R-mediated phagocytosis);map04713(Circadian entrainment);map04714(Thermogenesis);map04720(Long-term potentiation);map04722(Neurotrophin signaling pathway);map04723(Retrograde endocannabinoid signaling);map04724(Glutamatergic synapse);map04725(Cholinergic synapse);map04726(Serotonergic synapse);map04728(Dopaminergic synapse);map04730(Long-term depression);map04745(Phototransduction - fly);map04750(Inflammatory mediator regulation of TRP channels);map04911(Insulin secretion);map04912(GnRH signaling pathway);map04915(Estrogen signaling pathway);map04916(Melanogenesis);map04918(Thyroid hormone synthesis);map04919(Thyroid hormone signaling pathway);map04920(Adipocytokine signaling pathway);map04921(Oxytocin signaling pathway);map04923(Regulation of lipolysis in adipocytes);map04925(Aldosterone synthesis and secretion);map04926(Relaxin signaling pathway);map04928(Parathyroid hormone synthesis, secretion and action);map04929(GnRH secretion);map04931(Insulin resistance);map04933(AGE-RAGE signaling pathway in diabetic complications);map04935(Growth hormone synthesis, secretion and action);map04961(Endocrine and other factor-regulated calcium reabsorption);map04970(Salivary secretion);map04971(Gastric acid secretion);map04972(Pancreatic secretion);map04973(Carbohydrate digestion and absorption);map04975(Fat digestion and absorption);map05012(Parkinson disease);map05017(Spinocerebellar ataxia);map05022(Pathways of neurodegeneration - multiple diseases);map05110(Vibrio cholerae infection);map05131(Shigellosis);map05143(African trypanosomiasis);map05146(Amoebiasis);map05163(Human cytomegalovirus infection);map05167(Kaposi sarcoma-associated herpesvirus infection);map05170(Human immunodeficiency virus 1 infection);map05171(Coronavirus disease - COVID-19);map05200(Pathways in cancer);map05214(Glioma);map05223(Non-small cell lung cancer);map05225(Hepatocellular carcinoma);map05231(Choline metabolism in cancer);map05235(PD-L1 expression and PD-1 checkpoint pathway in cancer);map05415(Diabetic cardiomyopathy) | DG(15:0/18:4(6Z,9Z,12Z,15Z)/0:0);DG(18:4(6Z,9Z,12Z,15Z)/15:0/0:0);DG(15:0/0:0/18:4n3) | 0.68 | down | 0.03 | 0.56 | 1.54 |
| POS | POS_230.248_9.0656_1 | N,N-Dimethyldodecylamine N-oxide | C14H31NO | Organic nitrogen compounds | Organonitrogen compounds | Aminoxides | NA | NA | NA | Xestoaminol C;2-Tetradecanone;12-Methyltridecanal;Tetradecanal | 1.33 | up | 0.04 | 0.57 | 1.10 |
| NEG | NEG_337.312_5.8513_1 | FA 22:1 | C22H42O2 | Lipids and lipid-like molecules | Fatty Acyls | Fatty acid esters | NA | C08316 | map01040(Biosynthesis of unsaturated fatty acids) | Erucic acid;Cetoleic acid;Phytyl acetate;7,9-Docosanedione;6,8-Docosanedione;5,7-Docosanedione;4,6-Docosanedione;N-butyl Oleate;Brassidic acid;15-Docosenoic acid;Docosenic acid;Docosenoic acid;3-Eicosyne;5-Eicosyne;9-Icosyne;Labdane;Neophytadiene | 3.20 | up | 0.03 | 0.56 | 1.35 |
| NEG | NEG_783.577_4.0973_1 | Hyodeoxycholic acid | C24H40O4 | Lipids and lipid-like molecules | Steroids and steroid derivatives | Bile acids, alcohols and derivatives | HMDB0000733 | NA | NA | 3b,12a-Dihydroxy-5a-cholanoic acid;3b,7a-Dihydroxy-5b-cholanoic acid;3a,7a-Dihydroxycholanoic acid;3a,12b-Dihydroxy-5b-cholanoic acid;3b,12a-Dihydroxy-5b-cholanoic acid;Allodeoxycholic acid;Allochenodeoxycholic acid;Chenodeoxycholic acid;Isohyodeoxycholic acid;Hyodeoxycholic acid;Murocholic acid;Ursodeoxycholic acid;7b,12a-Dihydroxycholanoic acid;7a,12b-dihydroxy-5b-Cholan-24-oic acid;Isodeoxycholic acid;3,7-Dihydroxycholan-24-oic acid;3alpha,12beta-Dihydroxy-5alpha-cholan-24-oic Acid;4-[(5R,8S,10R,13R,17R)-3,6-Dihydroxy-10,13-dimethyl-2,3,4,5,6,7,8,9,11,12,14,15,16,17-tetradecahydro-1H-cyclopenta[a]phenanthren-17-yl]pentanoic acid;Methyl-[12]-gingerol;CE(6 keto-PGF1alpha);CE(TXB2) | 0.31 | down | 0.03 | 0.56 | 1.87 |
| POS | POS_114.091_2.9885_1 | 1-Piperidinecarboxaldehyde | C6H11NO | Organoheterocyclic compounds | Piperidines | NA | HMDB0031702 | NA | NA | 2,5-Dihydro-2,4,5-trimethyloxazole;1-Piperidinecarboxaldehyde;2-Acetylpyrrolidine;Epsilon-caprolactam;1-Ethyl-2-pyrrolidinone;2-Methoxyisobutylisonitrile;5,5-Dimethyl-1-pyrroline N-oxide;Cyclohexanone oxime;Methoxyisobutylisonitrile;N-ISOPROPYLACRYLAMIDE;2-Methyl-2-cyclopenten-1-one;3-Methyl-2-cyclopenten-1-one;2,4-Dimethylfuran;2,5-Dimethylfuran;(E,E)-2,4-Hexadienal;2-Ethylfuran;3-Ethylfuran;2-Cyclohexen-1-one;2,3-Dimethylfuran;Hexa-trans-2-cis-4-dien-1-al | 1.26 | up | 0.05 | 0.57 | 1.03 |
| POS | POS_241.03_0.5146_1 | L-Cystine | C6H12N2O4S2 | Organic acids and derivatives | Carboxylic acids and derivatives | Amino acids, peptides, and analogues | HMDB0000192 | C00491 | map00270(Cysteine and methionine metabolism);map01100(Metabolic pathways);map02010(ABC transporters);map04216(Ferroptosis);map04974(Protein digestion and absorption) | L-Cystine;Cystine;Diphenyl sulfone;Sodium gluconate | 0.27 | down | 0.03 | 0.56 | 1.64 |
| POS | POS_222.097_0.5072_1 | N-Acetyl-b-D-galactosamine | C8H15NO6 | Organic oxygen compounds | Organooxygen compounds | Carbohydrates and carbohydrate conjugates | HMDB0000853 | C05021 | NA | N-Acetylgalactosamine;N-Acetyl-D-glucosamine;2'-Deoxysepiapterin;beta-N-Acetylglucosamine;N-Acetyl-b-D-galactosamine;N-Acetylmannosamine;Avenic acid B;Aldehydo-N-acetyl-D-glucosamine;N-((2R,3R,4R,5R)-3,4,5,6-Tetrahydroxy-1-oxohexan-2-yl)acetamide;2-(Acetylamino)-2-deoxy-alpha-D-mannopyranose;(2R,5S)-5-Amino-1,2,6,7-tetrahydroxyoctane-3,4-dione;3,5,6-Trihydroxy-5-(hydroxymethyl)-2-methoxy-2-cyclohexen-1-one;2-(2-Hydroxyethyl)-4-oxohexanedioic acid | 0.48 | down | 0.01 | 0.53 | 2.42 |
| NEG | NEG_438.258_3.5244_1 | PE(15:0/0:0) | C20H41N1O7P1 | Lipids and lipid-like molecules | Glycerophospholipids | Glycerophosphoethanolamines | NA | C00350 | map00563(Glycosylphosphatidylinositol (GPI)-anchor biosynthesis);map00564(Glycerophospholipid metabolism);map01100(Metabolic pathways);map01110(Biosynthesis of secondary metabolites);map04136(Autophagy - other);map04138(Autophagy - yeast);map04140(Autophagy - animal);map04723(Retrograde endocannabinoid signaling);map05130(Pathogenic Escherichia coli infection);map05167(Kaposi sarcoma-associated herpesvirus infection) | Spergualin | 0.38 | down | 0.00 | 0.48 | 2.21 |
| POS | POS_415.212_4.6816_1 | Austinoneol | C24H30O6 | Organoheterocyclic compounds | Pyrans | Pyranones and derivatives | NA | NA | NA | 4-(Methylnitrosamino)-1-(3-pyridyl)-1-butanone;4-Carbamimidoyl-L-phenylalanine;M-Amidinophenyl-3-alanine;4'-(Nitrosomethylamino)-1-(3-pyridyl)-1-butanone;(2S)-2-(Diaminomethylideneamino)-3-phenylpropanoic acid;Eplerenone;Armillaripin;4-O-Methylmelleolide;Armillarin;Clausarinol;N-Palmitoyl Arginine;Trospium;Dihydrofukinolide;10-Hydroperoxy-H4-neuroprostane;11-Hydroperoxy-H4-neuroprostane;14-Hydroperoxy-H4-neuroprostane;N,N'-Dibenzhydrylethane-1,2-diamine;N'-[(6-Oxo-5-prop-2-enyl-1-cyclohexa-2,4-dienylidene)methyl]-2-[4-(phenylmethyl)-1-piperazinyl]acetohydrazide;Reduced ketanserin | 1.87 | up | 0.02 | 0.56 | 1.29 |
| POS | POS_269.161_1.0622_1 | Histidylleucine | C12H20N4O3 | Organic acids and derivatives | Carboxylic acids and derivatives | Amino acids, peptides, and analogues | HMDB0028889 | C05010 | NA | NA | 0.36 | down | 0.02 | 0.55 | 1.86 |
| NEG | NEG_243.063_1.1506_1 | Pseudouridine | C9H12N2O6 | Nucleosides, nucleotides, and analogues | Nucleoside and nucleotide analogues | NA | HMDB0000767 | C02067 | map00240(Pyrimidine metabolism);map01100(Metabolic pathways) | Uridine;Pseudouridine;1-[(2R,3S,5R)-3,4-Dihydroxy-5-(hydroxymethyl)oxolan-2-yl]pyrimidine-2,4-dione;1-[(2R,3R,4S,5R)-3,4-Dihydroxy-5-(hydroxymethyl)oxolan-2-yl]oxypyrimidin-2-one;Ethyl beta-D-glucopyranoside;Dambonitol;Ethyl glucoside;3-Methyl-1-propylxanthine;7-Ethyltheophylline;Isobutylxanthine;2'',6''-O-Diacetyloninin;Ethyl beta-D-fructofuranoside;Imidazotetrazine;Mimosine;2-Chloro-6-(1-piperazinyl)pyrazine;Trimidox | 0.57 | down | 0.05 | 0.57 | 1.16 |
| POS | POS_325.199_0.9893_1 | Quinidine hydrochloride monohydrate | C20H27ClN2O3 | Alkaloids and derivatives | Cinchona alkaloids | NA | NA | NA | NA | Lactapiperanol D;Cibaric acid;N1,N12-Diacetylspermine;S-Methyl hexadecanethioate;N-[4-[Acetyl(3-aminopropyl)amino]butyl]-N-(3-aminopropyl)acetamide;Emedastine;Arginyllysine;xi-7-Hydroxyhexadecanedioic acid;xi-8-Hydroxyhexadecanedioic acid;Alniditan;Gemcabene | 0.80 | down | 0.02 | 0.55 | 1.77 |
| NEG | NEG_308.099_0.6213_1 | N-Acetylneuraminate | C11H19NO9 | Organic oxygen compounds | Organooxygen compounds | Carbohydrates and carbohydrate conjugates | NA | C00270 | map00520(Amino sugar and nucleotide sugar metabolism);map00541(O-Antigen nucleotide sugar biosynthesis);map01100(Metabolic pathways);map01250(Biosynthesis of nucleotide sugars) | N-Acetylneuraminic acid;N-Acetyl-a-neuraminic acid;3h-Sialic acid;Glucose-6-glutamate;9-O-Acetylneuraminic acid;Aceneuramic acid;Neplanocin A;S-Acetyldihydrolipoamide;S-Acetyldihydrolipoamide-E;(1Z)-1-(3-Ethyl-5-methoxy-1,3-benzothiazol-2-ylidene)propan-2-one;4-Nitro-7-piperazinobenzofurazan;2',3'-Didehydro-2',3'-dideoxyguanosine;(E)-3-(4-Tert-butylphenylsulfonyl)acrylonitrile;TVP-1022 | 0.45 | down | 0.03 | 0.56 | 1.68 |
| POS | POS_416.363_8.2815_1 | beta-Tocopherol | C28H48O2 | Lipids and lipid-like molecules | Prenol lipids | Quinone and hydroquinone lipids | HMDB0006335 | C14152 | map00130(Ubiquinone and other terpenoid-quinone biosynthesis);map01100(Metabolic pathways);map01110(Biosynthesis of secondary metabolites);map01240(Biosynthesis of cofactors) | Nb-Palmitoyltryptamine | 1.87 | up | 0.04 | 0.57 | 1.82 |
| POS | POS_119.084_4.274_1 | alpha-Methylstyrene | C9H10 | Benzenoids | Benzene and substituted derivatives | Phenylpropenes | HMDB0059899 | C14395 | NA | Methylenecyclohexane | 2.38 | up | 0.01 | 0.53 | 1.52 |
| NEG | NEG_204.067_2.982_1 | Indole-3-lactic acid | C11H11NO3 | Organoheterocyclic compounds | Indoles and derivatives | Indolyl carboxylic acids and derivatives | HMDB0000671 | C02043 | NA | Cinnamoylglycine;Edulitine;Methyl 1-methoxy-1H-indole-3-carboxylate;3-Indolehydracrylic acid;5-Ethyl-5-phenyl-1,3-oxazolidine-2,4-dione;Methyl oxindole-3-acetate;Pyridoxaminium(1+);N-Hydroxy-1-aminonaphthalene;1-Amino-2-naphthol;2-Quinolinylmethanol;4-Amino-1-naphthol;Indole-3-carboxaldehyde;1-Hydroxyisoquinoline;4-Hydroxyquinoline;4,1-Benzoxazepine | 0.41 | down | 0.05 | 0.57 | 1.61 |
| POS | POS_370.331_5.6027_1 | N-Palmitoyl Leucine | C22H43NO3 | Organic acids and derivatives | Carboxylic acids and derivatives | Amino acids, peptides, and analogues | HMDB0241928 | NA | NA | N-Stearoyl GABA;N-Palmitoyl Isoleucine;N-Palmitoyl Leucine | 2.67 | up | 0.00 | 0.52 | 1.75 |
| NEG | NEG_152.036_0.2737_1 | 3-Amino-5-hydroxybenzoic acid | C7H7NO3 | Benzenoids | Benzene and substituted derivatives | Benzoic acids and derivatives | HMDB0304942 | C12107 | map01051(Biosynthesis of ansamycins);map01110(Biosynthesis of secondary metabolites) | 3-Hydroxyanthranilic acid;3-Aminosalicylic acid;Aminosalicylic Acid;Mesalazine;6-Methoxy-pyridine-3-carboxylic acid;2-Nitro-p-cresol;3-Methyl-4-nitrophenol;4-Nitroanisole;4-Nitrobenzyl alcohol;2,5-Dihydroxybenzamide;Salicylhydroxamic acid;2-Aminooxybenzoic acid;5-Formyl-2H-pyran-6-carboxamide;3-Amino-4-hydroxybenzoic acid;3-Amino-5-hydroxybenzoic acid;2-Amino-3-fluoropropanoic acid;2-Pyridinecarboxaldehyde;3-Pyridinecarboxaldehyde;4-Pyridinecarboxaldehyde;Fluoro-beta-alanine;Nitrosobenzene;p-Benzoquinone imine;3-Amino-3-fluoropropanoic acid | 1.28 | up | 0.02 | 0.56 | 1.05 |
| NEG | NEG_263.123_3.2757_1 | Alkhanin | C15H20O4 | Lipids and lipid-like molecules | Prenol lipids | Terpene lactones | HMDB0036202 | NA | NA | Meprobamate;Isoleucyl-Serine;Leucyl-Serine;Serylisoleucine;Serylleucine;L-Lysopine;Pantothenamide;(2R)-2-Amino-2-[[(1S)-1-carboxyethyl]amino]-4-methylpentanoic acid;N(6)-(1-Carboxyethyl)-L-lysine;Carboxyethyllysine;epsilon-(Carboxyethyl)lysine;1,3-Propanediol, 2,2-diethyl-, dicarbamate;N(epsilon)-(carboxyethyl)lysine;Ser-Leu;Threoninyl-Valine;Serylvaline;N6-Acetyl-5S-hydroxy-L-lysine;3,4-Dihydroxy-2-hydroxymethyl-1-pyrrolidinepropanamide;N6-Carboxymethyllysine;N-(3-Amino-2-hydroxy-3-oxopropyl)-L-valine;Carboxy methyl lysine;epsilon-(Carboxymethyl)lysine;n-carboxymethyllysine;2-[(2S)-2,6-Diaminohexanoyl]oxyacetic acid;[(2S)-6-Amino-1-methoxy-1-oxohexan-2-yl]carbamic acid | 0.39 | down | 0.02 | 0.56 | 1.50 |
| POS | POS_231.17_1.8891_1 | Leu-Val | C11H22N2O3 | Organic acids and derivatives | Carboxylic acids and derivatives | Amino acids, peptides, and analogues | HMDB0028942 | NA | NA | Isoleucyl-Valine;Leucyl-Valine;2-Decylfuran;(E)-6,10-Dimethyl-9-methylene-5-undecen-2-one;2-Cyclotetradecen-1-one;2-nonenoylglycine;3-nonenoylglycine;4-nonenoylglycine;5-nonenoylglycine;6-nonenoylglycine;7-nonenoylglycine;8-nonenoylglycine;Ecgonine Ethyl Ester | 0.50 | down | 0.03 | 0.56 | 1.48 |
| POS | POS_858.54_6.4497_1 | SQDG(18:2/18:3) | C45H80N1O12S1 | Lipids and lipid-like molecules | Glycerolipids | Glycosylglycerols | NA | C13508 | map00561(Glycerolipid metabolism);map01100(Metabolic pathways) | PE(20:0/22:6(4Z,7Z,10Z,13Z,16Z,19Z));PE(20:1(11Z)/22:5(4Z,7Z,10Z,13Z,16Z));PE(20:1(11Z)/22:5(7Z,10Z,13Z,16Z,19Z));PE(20:2(11Z,14Z)/22:4(7Z,10Z,13Z,16Z));PE(20:4(5Z,8Z,11Z,14Z)/22:2(13Z,16Z));PE(20:4(8Z,11Z,14Z,17Z)/22:2(13Z,16Z));PE(20:5(5Z,8Z,11Z,14Z,17Z)/22:1(13Z));PE(22:1(13Z)/20:5(5Z,8Z,11Z,14Z,17Z));PE(22:2(13Z,16Z)/20:4(5Z,8Z,11Z,14Z));PE(22:2(13Z,16Z)/20:4(8Z,11Z,14Z,17Z));PE(22:4(7Z,10Z,13Z,16Z)/20:2(11Z,14Z));PE(22:5(4Z,7Z,10Z,13Z,16Z)/20:1(11Z));PE(22:5(7Z,10Z,13Z,16Z,19Z)/20:1(11Z));PE(22:6(4Z,7Z,10Z,13Z,16Z,19Z)/20:0);PE-NMe2(18:0/22:6(4Z,7Z,10Z,13Z,16Z,19Z));PE-NMe2(18:1(11Z)/22:5(4Z,7Z,10Z,13Z,16Z));PE-NMe2(18:1(11Z)/22:5(7Z,10Z,13Z,16Z,19Z));PE-NMe2(18:1(9Z)/22:5(4Z,7Z,10Z,13Z,16Z));PE-NMe2(18:1(9Z)/22:5(7Z,10Z,13Z,16Z,19Z));PE-NMe2(18:2(9Z,12Z)/22:4(7Z,10Z,13Z,16Z));PE-NMe2(18:4(6Z,9Z,12Z,15Z)/22:2(13Z,16Z));PE-NMe2(20:1(11Z)/20:5(5Z,8Z,11Z,14Z,17Z));PE-NMe2(20:2(11Z,14Z)/20:4(5Z,8Z,11Z,14Z));PE-NMe2(20:2(11Z,14Z)/20:4(8Z,11Z,14Z,17Z));PE-NMe2(20:3(5Z,8Z,11Z)/20:3(5Z,8Z,11Z));PE-NMe2(20:3(5Z,8Z,11Z)/20:3(8Z,11Z,14Z));PE-NMe2(20:3(8Z,11Z,14Z)/20:3(5Z,8Z,11Z));PE-NMe2(20:3(8Z,11Z,14Z)/20:3(8Z,11Z,14Z));PE-NMe2(20:4(5Z,8Z,11Z,14Z)/20:2(11Z,14Z));PE-NMe2(20:4(8Z,11Z,14Z,17Z)/20:2(11Z,14Z));PE-NMe2(20:5(5Z,8Z,11Z,14Z,17Z)/20:1(11Z));PE-NMe2(22:2(13Z,16Z)/18:4(6Z,9Z,12Z,15Z));PE-NMe2(22:4(7Z,10Z,13Z,16Z)/18:2(9Z,12Z));PE-NMe2(22:5(4Z,7Z,10Z,13Z,16Z)/18:1(11Z));PE-NMe2(22:5(4Z,7Z,10Z,13Z,16Z)/18:1(9Z));PE-NMe2(22:5(7Z,10Z,13Z,16Z,19Z)/18:1(11Z));PE-NMe2(22:5(7Z,10Z,13Z,16Z,19Z)/18:1(9Z));PE-NMe2(22:6(4Z,7Z,10Z,13Z,16Z,19Z)/18:0);PC(17:0/22:6(4Z,7Z,10Z,13Z,16Z,19Z));PC(17:2(9Z,12Z)/22:4(7Z,10Z,13Z,16Z));Sulfoquinovosyl diglyceride | 0.31 | down | 0.02 | 0.56 | 2.27 |
| NEG | NEG_157.087_3.2109_1 | 5-Oxooctanoic acid | C8H14O3 | Lipids and lipid-like molecules | Fatty Acyls | Fatty acids and conjugates | HMDB0341004 | NA | NA | cis-4-Hydroxycyclohexylacetic acid;trans-4-Hydroxycyclohexylacetic acid;3-Oxooctanoic acid;Alpha-Ketooctanoic acid;6-Ethyl-1-methyl-2,7,8-trioxabicyclo[3.2.1]octane;Tetrahydrofurfuryl propionate;Ethyl 3-oxohexanoate;1-Methyl-2-oxopropyl butyrate;2-Methylpropyl 3-oxobutanoate;3-Methylbutyl 2-oxopropanoate;Butyl acetoacetate;Propyl levulinate;5-Butyl-1,4-dioxan-2-one;6-Butyl-1,4-dioxan-2-one;2-n-Propyl-4-oxopentanoic acid;3-Oxovalproic acid;Isobutyric Acid Anhydride;Butanoic anhydride;(Z)-4-Heptenal;3-Hepten-2-one;(E)-4-Hepten-2-one;2-Hepten-4-one;2-Methylcyclohexanone;4-Methylcyclohexanone;5-Methyl-3-hexen-2-one;5-Methyl-5-hexen-2-one;trans-2-trans-4-Heptadien-1-ol;(R)-3-Methylcyclohexanone;(2E)-2-Heptenal;xi-3-Methyl-3-cyclohexen-1-ol;2,5-Heptadien-1-ol;1-hepten-3-one;1-methoxycyclohexene;Cyclohexanone;2-Methyl-4-pentenal;4-Methyl-2-pentenal;2-Hexenal;3-Hexenal;4-Methyl-3-penten-2-one, 9CI;4-Methyl-4-penten-2-one;(E)-4-Hexenal;2-Methyl-2-pentenal;4-Hexen-3-one;(Z)-2-Hexenal;(Z)-4-Hexenal;cis-Cyclohexene Oxide;1,4-Epoxycyclohexane;3-Methyl-1-pentyn-3-ol;Allyl ether;Hexenal;Hex-cis-3-en-1-al;2,4-Hexadien-1-ol;1-hexen-3-one | 1.57 | up | 0.05 | 0.57 | 1.04 |
| NEG | NEG_187.109_1.2368_1 | Glycyl-L-leucine | C8H16N2O3 | Organic acids and derivatives | Carboxylic acids and derivatives | Amino acids, peptides, and analogues | NA | C02155 | NA | N6-Acetyl-L-lysine;N-alpha-Acetyl-L-lysine;Glycylleucine;Alanylvaline;Glycyl-Isoleucine;Isoleucyl-Glycine;Leucyl-Glycine;N-(3-Amino-3-oxopropyl)-L-valine;alpha-Acetyllysine;DIBOA tetrahexose;piperidine-1-carboxamide;Polylysine | 0.45 | down | 0.02 | 0.56 | 1.74 |
| NEG | NEG_195.051_0.6062_1 | Gluconic acid | C6H12O7 | Organic oxygen compounds | Organooxygen compounds | Carbohydrates and carbohydrate conjugates | HMDB0000625 | C00257 | map00030(Pentose phosphate pathway);map01100(Metabolic pathways);map01110(Biosynthesis of secondary metabolites);map01120(Microbial metabolism in diverse environments);map01200(Carbon metabolism) | Galactonic acid;Gluconic acid;1,3-Dimethyluric acid;3,7-Dimethyluric acid;1,9-Dimethyluric acid;Gulonic acid;7,9-Dimethyluric acid;1,7-Dimethyluric acid;3,9-Dimethyluric acid;D-Mannonic acid;L-Gluconic acid;2-Carboxyarabinitol;D-Xylose;D-Ribose;2-Deoxyribonic acid;7-Methylhypoxanthine;Arabinofuranose;1-Methylhypoxanthine;Diisopropyl disulfide;D-Apiose;Methyl pentyl disulfide;Dipropyl disulfide;1,6-Hexanedithiol;Butyl ethyl disulfide;Methyl isopentyl disulfide;xi-1-(Propylthio)-1-propanethiol;2-Deoxypentonic acid;(2R,3R,4R)-2,3,4,5-Tetrahydroxypentanal;9-Deazaguanine;alpha-D-Arabinofuranose;D-Arabinopyranose;D-Threo-3-Pentulose;Isopropyl propyl disulfide;alpha-L-arabinofuranose;Hypoxanthine;Erythronic acid;Threonic acid;Allopurinol;1-Pentanesulfenothioic acid;Ethyl propyl disulfide;Ethyl isopropyl disulfide;2-Hydroxypurine;2,3,4-Trihydroxybutanoic acid;9H-Purine-9-ol | 0.59 | down | 0.04 | 0.57 | 1.69 |
| NEG | NEG_241.083_1.9327_1 | thymidine | C10H14N2O5 | Nucleosides, nucleotides, and analogues | Pyrimidine nucleosides | Pyrimidine 2'-deoxyribonucleosides | HMDB0000273 | C00214 | map00240(Pyrimidine metabolism);map01100(Metabolic pathways);map01232(Nucleotide metabolism) | Thymidine;Telbivudine;L-Thymidine;2-(Ethylamino)-4,5-dihydroxybenzamide;5-Nitro-2-propoxyaniline;3-Amino-L-tyrosine;4-Amino-3-hydroxyphenylalanine;5-Aminosalicyl-glycine;Aethallymal;3,4-Dephostatin, Ethyl-;Diisopropylphosphate;TRIETHYL PHOSPHATE;Potassium octanoate | 0.25 | down | 0.04 | 0.57 | 1.76 |
| NEG | NEG_225.07_3.1447_1 | Genipin | C11H14O5 | Lipids and lipid-like molecules | Prenol lipids | Monoterpenoids | HMDB0035830 | C09780 | NA | 1-(4-Methoxyphenyl)-1-penten-3-one;3-(2-Methylpropylidene)-3alpha,4-dihydro-1(3H)-isobenzofuranone;(S)-3-Butyl-1(3H)-isobenzofuranone;Benzyl trans-2-methyl-2-butenoate;(+/-)-Isobutyl 3-methylthiobutyrate;3-Mercaptoheptyl acetate;(S)-3-Methylthiohexyl acetate;(R)-3,7-Dimethyl-5-indanecarboxylic acid;Ligustilide;Benzyl tiglate;Propyl cinnamate;Isopropyl cinnamate;Cinnamyl propionate;Precocene I;3-Butylidene-4,5-dihydrophthalide;Ligusticide;Cyclosarin;Protionamide;Ethionamide;Carpasemine | 0.41 | down | 0.02 | 0.54 | 1.58 |
| POS | POS_318.181_3.1113_1 | Trp Leu | C17H23N3O3 | NA | NA | NA | NA | NA | NA | Isoleucyl-Tryptophan;Leucyl-Tryptophan;Tryptophyl-Isoleucine;Tryptophyl-Leucine;Glemanserin;Methoxphenidine;Normethadone;Pridinol;N-(4-Amino-2,5-diethoxyphenyl)benzamide;2-trans,-6-trans-farnesyl monophosphate | 0.54 | down | 0.02 | 0.56 | 1.34 |
| NEG | NEG_292.14_1.1885_1 | N-Fructosyl isoleucine | C12H23NO7 | Organic acids and derivatives | Carboxylic acids and derivatives | Amino acids, peptides, and analogues | NA | NA | NA | N-(1-Deoxy-1-fructosyl)leucine;N-(1-Deoxy-1-fructosyl)isoleucine;3,4,5-Trihydroxypentanoylcarnitine;3-Hydroxybutyrylcarnitine;(R)-3-hydroxybutyrylcarnitine;Peramine;Hydroxypropionylcarnitine;2-Heptylbenzothiazole;2-Phenyl-4,4,5,5-tetramethylimidazoline-1-oxyl 3-oxide;4-[(2,4-Dihydroxy-3,3-dimethylbutanoyl)amino]butanoic acid;7-Benzyl-3-thia-7-azabicyclo[3.3.1]nonane;Nitrazine | 0.66 | down | 0.03 | 0.56 | 1.99 |
| POS | POS_414.337_3.7352_1 | Solasodine | C27H43NO2 | Lipids and lipid-like molecules | Steroids and steroid derivatives | Steroidal alkaloids | HMDB0035282 | C10822 | map01066(Biosynthesis of alkaloids derived from terpenoid and polyketide) | 5alpha-Tomatidan-3-one;Solasodine;delta3,5-Deoxytigogenin;delta3,5-Deoxyneotigogenin;alpha-Micropteroxanthin B;beta-Micropteroxanthin;(2R)-2,8-Dimethyl-2-(4,8,12-trimethyltrideca-3,7,11-trienyl)-3,4-dihydrochromen-6-ol;Spirosta-3,5-diene | 0.29 | down | 0.03 | 0.56 | 1.66 |
| POS | POS_432.237_4.7282_1 | 7b,9-Dihydroxy-3-(hydroxymethyl)-1,1,6,8-tetramethyl-5-oxo-1,1a,1b,4,4a,5,7a,7b,8,9-decahydro-9aH-cyclopropa[3,4]benzo[1,2-e]azulen-9a-yl acetate | C22H30O6 | Lipids and lipid-like molecules | Prenol lipids | Diterpenoids | NA | NA | NA | Tropolone A;undecylprodigiosin;(3E,5Z,11Z)-Pentadeca-3,5,11-trienedioylcarnitine;Pentadeca-9,11,13-trienedioylcarnitine;Pentadeca-3,6,9-trienedioylcarnitine;Pentadeca-7,10,13-trienedioylcarnitine;Pentadeca-4,6,8-trienedioylcarnitine;Pentadeca-5,8,11-trienedioylcarnitine;(2E,6E,10E)-Pentadeca-2,6,10-trienedioylcarnitine;Pentadeca-5,7,9-trienedioylcarnitine;Pentadeca-3,5,7-trienedioylcarnitine;Eplerenone;Armillaripin;4-O-Methylmelleolide;Armillarin;Clausarinol | 2.48 | up | 0.01 | 0.53 | 1.96 |
| POS | POS_306.145_2.8147_1 | Thr Trp | C15H19N3O4 | NA | NA | NA | NA | NA | NA | Tryptophyl-Threonine;Imazamox;Threoninyl-Tryptophan;Metoprolol;Desacetylmetipranolol;Butaxamine;Fospropofol;3-Carboxy-2,3,4,9-tetrahydro-1H-pyrido[3,4-b]indole-1-propanoic acid;(R)-Myclobutanil;5-(Aziridin-1-yl)-3-(hydroxymethyl)-2-(3-hydroxyprop-1-enyl)-1-methylindole-4,7-dione | 0.21 | down | 0.04 | 0.56 | 1.45 |
| POS | POS_511.303_4.3143_1 | PG(18:1(9Z)/0:0) | C24H47O9P | Lipids and lipid-like molecules | Glycerophospholipids | Glycerophosphoglycerols | NA | C00344 | map00552(Teichoic acid biosynthesis);map00564(Glycerophospholipid metabolism);map01100(Metabolic pathways) | Desmethyl rizatriptan;LysoPG(18:1(9Z)/0:0);Antcin K;4-[[2-Methyl-2-[4-[5-(trifluoromethyl)pyridin-2-yl]piperazin-1-yl]propanoyl]amino]adamantane-1-carboxamide | 2.37 | up | 0.02 | 0.54 | 1.40 |
| NEG | NEG_363.182_3.0923_1 | Cinncassiol C2 | C20H28O6 | Lipids and lipid-like molecules | Prenol lipids | Sesquiterpenoids | HMDB0035168 | C17642 | NA | xml:space="preserve">12-Oxo-20-carboxy-leukotriene B4;Eremopetasitenin A1;Cinncassiol C2;Blumealactone A;Blumealactone B;Gibberellin A123;Gibberellin A74;3b,8a-Dihydroxy-6b-angeloyloxy-7(11)-eremophilen-12,8-olide;3b,8b-Dihydroxy-6b-angeloyloxy-7(11)-eremophilen-12,8-olide;3b,9b-Dihydroxy-6b-angeloyloxy-7(11)-eremophilen-12,8b-olide;3b-Hydroxy-6b-(2,3-epoxy-2-methylbutanoyloxy)-7(11)-eremophilen-12,8b-olide;Gibberellin A100;Gibberellin A97;5-(4'-Hydroxyphenyl)-gamma-valerolactone-4'-O-glucuronide;Oridonin;4alpha-Phorbol;(2R,5R)-2-[6-(Cyclopentylamino)-8-(methylamino)purin-9-yl]-5-(hydroxymethyl)oxolane-3,4-diol;7-Methyl-2-morpholino-9-(1-(phenylamino)ethyl)-4H-pyrido[1,2-a]pyrimidin-4-one;(1S,2S,3S,4R)-3-(1-Acetamido-2-ethylbutyl)-4-(diaminomethylideneamino)-2-hydroxycyclopentane-1-carboxylic acid;Amyl 2-furoate;(&#177;)-threo-Anethole glycol;4-Ethyl-2,6-dimethoxyphenol;Verimol J;4-(Ethoxymethyl)-2-methoxyphenol;Furfuryl pentanoate;Peperinic acid;2-Ethoxy-4-(methoxymethyl)phenol;Furfuryl isovalerate;Isoamyl 2-furoate;(S)-3-(2-Methylphenoxy)propane-1,2-diol;(&#172;&#177;)-erythro-Anethole glycol;1,2,3-Trimethoxy-5-methylbenzene;Dihydroconiferyl alcohol;Ubiquinone-2;Cohulupone;(1(10)E,4E,6a,9b)-9-(2-Methylpropanoyloxy)-1(10),4,11(13)-germacratrien-12,6-olide;Panaquinquecol 6;[8]-Dehydrogingerdione;Ubiquinones;2-Methoxyestriol;1-Dehydro-[8]-gingerdione;2-Polyprenyl-3-methyl-5-hydroxy-6-methoxy-1,4-benzoquinone;3-Polyprenyl-4-hydroxy-5-methoxybenzoate;2-Hydroxyestriol;Tert-butyl 4-(1H-pyrazolo[3,4-d]pyrimidin-4-yl)piperazine-1-carboxylate;Estetrol;Estetrol&#160; | 0.11 | down | 0.04 | 0.57 | 1.82 |
| POS | POS_189.123_1.1999_1 | N-Glycyl-L-leucine | C8H16N2O3 | Organic acids and derivatives | Carboxylic acids and derivatives | Amino acids, peptides, and analogues | HMDB0000759 | C02155 | NA | N6-Acetyl-L-lysine;N-alpha-Acetyl-L-lysine;Glycylleucine;Alanylvaline;Glycyl-Isoleucine;Isoleucyl-Glycine;Leucyl-Glycine;N-(3-Amino-3-oxopropyl)-L-valine;alpha-Acetyllysine;DIBOA tetrahexose;N-[(3s)-2-Oxotetrahydrofuran-3-Yl]butanamide;Diethadione | 0.62 | down | 0.04 | 0.57 | 1.32 |
| POS | POS_277.068_0.9623_1 | 5,7-dinitro-2,3-dihydropyrrolo[2,1-b]quinazolin-9(1H)-one | C11H8N4O5 | Organoheterocyclic compounds | Diazanaphthalenes | Benzodiazines | NA | NA | NA | 4-Hydroxybenzoic acid;Salicylic acid;3-Hydroxybenzoic acid;Gentisate aldehyde;2-Methoxy-1,4-benzoquinone;alpha-Furyl methyl diketone;Sesamol;3,4-Dihydroxybenzaldehyde;keratan sulfate II (core 2-linked), degradation product 1;3,6-Dihydroxycyclohepta-2,4,6-trien-1-one;O-Demethylfonsecin;1-(3-Fluoro-4-hydroxy-5-mercaptomethyltetrahydrofuran-2-yl)-5-methylpyrimidine-2,4(1H,3H)-dione;Biotin thiamine;(x)-1,2-Propanediol 1-O-b-D-glucopyranoside;Proxyphylline;[(2S,3S,4R,5R)-3,4,5,6-Tetrahydroxyhexan-2-yl] 2-hydroxypropanoate;L-Phosphoarginine;2'-Fluoro-2',3'-dideoxyinosine;Bis(2-hydroxyethyl) terephthalate;Glucosamine 6-sulfate;N-Sulfo-D-glucosamine | 0.20 | down | 0.03 | 0.56 | 2.00 |
| POS | POS_219.134_1.1238_1 | Ser-Leu | C9H18N2O4 | Organic acids and derivatives | Carboxylic acids and derivatives | Amino acids, peptides, and analogues | HMDB0029043 | NA | NA | 2-Hydrazinopyridine;3,4-Diaminopyridine;Meprobamate;Isoleucyl-Serine;Leucyl-Serine;Serylisoleucine;Serylleucine;L-Lysopine;Pantothenamide;(2R)-2-Amino-2-[[(1S)-1-carboxyethyl]amino]-4-methylpentanoic acid;N(6)-(1-Carboxyethyl)-L-lysine;Carboxyethyllysine;epsilon-(Carboxyethyl)lysine;1,3-Propanediol, 2,2-diethyl-, dicarbamate;N(epsilon)-(carboxyethyl)lysine;Ser-Leu;Threoninyl-Valine;Dihydro-5-(2-octenyl)-2(3H)-furanone;Methyl 2-undecynoate;6-Heptyl-5,6-dihydro-2H-pyran-2-one;cis-3-Hexenyl trans-4-hexenoate;alpha-Terpineol acetate;alpha-Campholene acetate;(Z)-4-Hydroxy-6-dodecenoic acid lactone;Yuzu lactone;(3R,3aR,7aS)-3-Butylhexahydro-1(3H)-isobenzofuranone;Artemisia alcohol acetate;Ethyl 2Z,4E-decadienoic acid;p-Menth-1-en-9-ol acetate;cis-3-Hexenyl cis-3-hexenoate;cis-3-Hexenyl trans-2-hexenoate;Linalyl acetate;Allyl 3-cyclohexylpropionate;Isopulegol acetate;(-)-Bornyl acetate;Fenchyl acetate;Dihydrocarveol acetate;(S)-Santolina acetate;3,7-Dimethyl-2,6-octadien-1-ylacetate;Pentamethylmelamine;Dodecadienoic acid;cis-Sabinene hydrate acetate;alpha-Fenchyl acetate;(&#172;&#177;)-Isobornyl acetate;7-Acetoxy-7-methyl-3-methylene-1-octene;(+)-beta-Fenchyl acetate | 0.53 | down | 0.03 | 0.56 | 1.32 |
| NEG | NEG_193.035_1.5333_1 | 5-Keto-D-gluconate | C6H10O7 | Organic acids and derivatives | Organic acids and derivatives | Medium-chain hydroxy acids and derivatives | HMDB0011731 | C01062 | NA | Galacturonic acid;Iduronic acid;Pectic acid;Pectin;3-Dehydro-L-gulonate;5-Keto-D-gluconate;2-Keto-L-gluconate;6-(Hydroxymethyl)-2,4(1H,3H)-pteridinedione;L-Altruronic acid;Aldehydo-L-iduronate;(2R,3R,4R,5S,6S)-3,4,5,6-Tetrahydroxyoxane-2-carboxylic acid;Lactate oxygen;theophylline one;Mannuronic acid;Citramalic acid;3-Hydroxyglutaric acid;D-2-Hydroxyglutaric acid;L-2-Hydroxyglutaric acid;Ribonolactone;D-Xylono-1,5-lactone;2-Propenyl propyl disulfide;Methyl 3-methyl-1-butenyl disulfide;1-Propenyl propyl disulfide;3-methylmalate(2-);monoacetone;Poly(lactic acid-co-glycolic acid);D-Arabinono-1,4-lactone;trans-Propenyl propyl disulfide;Malic acid;D-Malic acid;Velcorin;3,3-Dimethyl-1,2-dithiolane;Tetrahydro-2-methyl-2-thiophenethiol;Tetrahydro-2-methyl-3-thiophenethiol;Diglycolic acid;Pyrazolopyrimidinone;2-Carboxyoxypropanoic acid;1,2-Dimercaptocyclopentane | 0.36 | down | 0.01 | 0.53 | 2.16 |
| POS | POS_507.344_3.5012_1 | Asn-CDCA | C28H46N2O6 | Lipids and lipid-like molecules | Steroids and steroid derivatives | Bile acids, alcohols and derivatives | NA | NA | NA | 1R-cis-3,3,5-Trimethylcyclohexyl ester5-oxo-L-proline;O-Demethylmetoprolol;Chenodeoxycholylasparagine;Deoxycholylasparagine;Anadur;26-Methyl nigranoate;3b,18b-3-Methoxy-11-oxo-12-oleanen-30-oic acid;Methyl 3b,24-dihydroxy-11,13(18)-oleanadien-30-oate;2,6-Ditert-butyl-4-[3-(3,5-ditert-butyl-4-hydroxyphenoxy)propoxy]phenol;Dehydrotumulosic acid | 0.82 | down | 0.03 | 0.56 | 1.16 |
| POS | POS_256.264_6.4796_1 | Hexadecanamide | C16H33NO | Lipids and lipid-like molecules | Fatty Acyls | Fatty amides | HMDB0012273 | NA | NA | Palmitic amide;3-Methylcyclopentadecanone;(&#177;)-2-Dodecylcyclobutanone;Hexadecenal;(Z)-11-Hexadecenal;Hexadec-2-enal;9-Hexadecenal, (9Z)- | 1.63 | up | 0.01 | 0.53 | 1.22 |
| NEG | NEG_227.067_1.3776_1 | 2-deoxyuridine | C9H12N2O5 | Nucleosides, nucleotides, and analogues | Pyrimidine nucleosides | Pyrimidine 2'-deoxyribonucleosides | NA | C00526 | map00240(Pyrimidine metabolism);map01100(Metabolic pathways);map01232(Nucleotide metabolism);map02010(ABC transporters) | Deoxyuridine;Pemirolast;1-((2S,4S,5R)-4-Hydroxy-5-(hydroxymethyl)tetrahydrofuran-2-yl)pyrimidine-2,4(1H,3H)-dione;1-[(2R,4R,5R)-3,4-Dihydroxy-5-(hydroxymethyl)-2-oxolanyl]-2-pyrimidinone;Antigastrin;Dioxolane-THYMINE;(5S,6R)-Methyl 5,6,7-trihydroxyheptanoate;3,4-Dephostatin, Ethyl-;Diisopropylphosphate;TRIETHYL PHOSPHATE;Potassium octanoate;2,3-Diaminosalicylic acid;2-Pyrroloylglycine;Dephostatin;2,6-diamino-4-hydroxy-5-formamidopyrimidine | 0.29 | down | 0.04 | 0.57 | 1.57 |
| NEG | NEG_791.499_6.0513_1 | SQDG 16:0_16:1 | C41H76O12S | Lipids and lipid-like molecules | Glycerolipids | Glycosylglycerols | NA | C13508 | map00561(Glycerolipid metabolism);map01100(Metabolic pathways) | PA(19:0/20:3(6,8,11)-OH(5));PA(20:3(6,8,11)-OH(5)/19:0);PA(21:0/18:2(10E,12Z)+=O(9));PA(18:2(10E,12Z)+=O(9)/21:0);PA(21:0/18:2(9Z,11E)+=O(13));PA(18:2(9Z,11E)+=O(13)/21:0);PA(21:0/18:3(10,12,15)-OH(9));PA(18:3(10,12,15)-OH(9)/21:0);PA(21:0/18:3(9,11,15)-OH(13));PA(18:3(9,11,15)-OH(13)/21:0);PA(a-21:0/18:2(10E,12Z)+=O(9));PA(18:2(10E,12Z)+=O(9)/a-21:0);PA(a-21:0/18:2(9Z,11E)+=O(13));PA(18:2(9Z,11E)+=O(13)/a-21:0);PA(a-21:0/18:3(10,12,15)-OH(9));PA(18:3(10,12,15)-OH(9)/a-21:0);PA(a-21:0/18:3(9,11,15)-OH(13));PA(18:3(9,11,15)-OH(13)/a-21:0);PA(i-19:0/20:3(6,8,11)-OH(5));PA(20:3(6,8,11)-OH(5)/i-19:0);PA(i-21:0/18:2(10E,12Z)+=O(9));PA(18:2(10E,12Z)+=O(9)/i-21:0);PA(i-21:0/18:2(9Z,11E)+=O(13));PA(18:2(9Z,11E)+=O(13)/i-21:0);PA(i-21:0/18:3(10,12,15)-OH(9));PA(18:3(10,12,15)-OH(9)/i-21:0);PA(i-21:0/18:3(9,11,15)-OH(13));PA(18:3(9,11,15)-OH(13)/i-21:0) | 0.39 | down | 0.02 | 0.54 | 1.81 |
| POS | POS_203.139_1.1275_1 | Ala-Leu | C9H18N2O3 | Organic acids and derivatives | Carboxylic acids and derivatives | Amino acids, peptides, and analogues | HMDB0028691 | NA | NA | Alanylisoleucine;Alanylleucine;Isoleucyl-Alanine;N-Acetylisoputreanine;n2-Acetyl,n6-methyllysine;DL-Alanyl-DL-leucine;Leucyl-Alanine;Homodihydrojasmone;2-trans-6-cis-Dodecadienal;cis-Quinceoxepane;Tricycloekasantalol;(2E,4E)-2,4-Dodecadienal;Pseudoecgonine;Ecgonine;2-Hepteneoylglycine;3-Hepteneoylglycine;4-Hepteneoylglycine;5-Hepteneoylglycine;3h-Adrenaline | 0.22 | down | 0.05 | 0.57 | 1.54 |
| NEG | NEG_270.216_4.5742_1 | Tridecanoylglycine | C15H29NO3 | Organic acids and derivatives | Carboxylic acids and derivatives | Amino acids, peptides, and analogues | HMDB0013317 | NA | NA | NA | 2.62 | up | 0.00 | 0.52 | 2.14 |
| NEG | NEG_150.042_1.0237_1 | 8-Hydroxyadenine | C5H5N5O | Organoheterocyclic compounds | Imidazopyrimidines | Purines and purine derivatives | HMDB0000542 | C22499 | map00230(Purine metabolism) | Guanine;2-Hydroxyadenine;8-Hydroxyadenine;8-Oxo-7,8-dihydrodeoxyguanine;(S)-2-Amino-4-methylsulfanyl-butane-1-thiol;L-Serine;D-Serine;2,3-Dihydroxypropanamide;3-Nitropropanol;Aminohydroxyacetic acid;Aminooxyacetic acid;Ethyl nitrate | 0.36 | down | 0.01 | 0.52 | 1.97 |
| POS | POS_321.24_5.6325_1 | 15-deoxy-Delta-12,14-PGJ2-d4 | C20H24D4O3 | NA | NA | NA | NA | NA | NA | 5,6-Epoxy-8,11,14-eicosatrienoic acid;8,9-Epoxyeicosatrienoic acid;14R,15S-EpETrE;14,15-Epoxy-5,8,11-eicosatrienoic acid;8-HETE;16(R)-HETE;11(R)-HETE;18-Hydroxyarachidonic acid;9-HETE;19(S)-HETE;10-HETE;13-HETE;17-HETE;Annoglabasin E;Ucriol;8alpha,13R-Epoxy-14-labden-19-oic acid;2-Hydroxy-6-tridecylbenzoic acid;(ent-2alpha,3beta,15beta,16beta)-15,16-Epoxy-2,3-kauranediol;(ent-2alpha,3beta,15beta)-16-Kaurene-2,3,15-triol;Crispane;12 Hydroxy arachidonic acid;12S-hydroxy-5E,8Z,10Z,14Z-eicosatetraenoic acid;15R-hydroxy-5Z,8Z,11Z,13E-eicosatetraenoic acid;18-Hydroxy-5Z,8Z,11Z,14Z-eicosatetraenoic acid;7-HETE;8-hydroxy-5Z,9E,11Z,14Z-eicosatetraenoic acid;12,15-Epoxy-13,14-dimethyloctadeca-10,12,14-trienoic acid;12,15-Epoxy-13,14-dimethyloctadeca-12,14,16-trienoic acid;(12S)-12-Hydroxyicosa-2,4,6,8-tetraenoic acid;(14R,15S)-14,15-Epoxy-5,8,11-icosatrienoic acid;(5E,8E,11E,14E)-19-Hydroxyicosa-5,8,11,14-tetraenoic acid;11-Hydroxy-5Z,8Z,11E,14Z-eicosatetraenoic acid;11,12-Epoxyeicosantrienoic acid;11R,12S-EpETrE;12(S)-Hydroxy-5,8,10,14-eicosatetraenoic acid;15-Hydroxy-5,8,11,13-eicosatetraenoic acid;Eicosatetraenoic acid, 15-hydroxy-;18-Hydroxyeicosatetraenoic acid;19-Hydroxyeicosatetraenoic acid;20-Hydroxyarachlidonic acid;4-[3-(Tetradeca-2,5,8-trien-1-yl)oxiran-2-yl]butanoic acid;20-Hydroxyeicosatetraenic acid;8-Hydroxy-5E,9Z,11Z,14Z-eicosatetraenoic acid;(Z)-7-((2S,3R)-3-((2Z,5Z)-Undeca-2,5-dienyl)oxiran-2-yl)hept-5-enoic acid;arachidonic acid hydroperoxide;Epoxyeicosatrienoic acid;Isocupressic acid;Mutilin;13-(3-Pentyloxiran-2-yl)trideca-2,4,6-trienoic acid;3-Heptadeca-1,3-dienyloxirene-2-carboxylic acid;5-Oxoicosa-3,6,8-trienoic acid;3-Oxooctadecanoic acid;9-Oxooctadecanoic acid;10-Oxooctadecanoic acid;11-Oxooctadecanoic acid;5-Hexyltetrahydro-2-furanoctanoic acid;5-Oxooctadecanoic acid;Ricinoleic acid;(12R)-12-Hydroxyoctadec-9-enoic acid;9,10-Epoxystearic acid;Methyl palmoxirate;3-hydroxyoctanoyl carnitine;5-Hydroxyoctanoylcarnitine;7-Hydroxyoctanoylcarnitine;6-Hydroxyoctanoylcarnitine;4-Hydroxyoctanoylcarnitine;5-Hydroxy-2-propylpentanoylcarnitine;3-v-2-propylpentanoylcarnitine;4-Hydroxy-2-propylpentanoylcarnitine;1-[(5-Methoxy-2,3-dihydro-1H-indol-3-yl)methylideneamino]-2-pentylguanidine | 1.98 | up | 0.01 | 0.52 | 1.49 |
| NEG | NEG_607.472_5.8714_1 | FAHFA 20:4/20:3 | C40H64O4 | Lipids and lipid-like molecules | Fatty Acyls | Fatty acid esters | NA | NA | NA | Arachidonic acid;Cis-8,11,14,17-Eicosatetraenoic acid;Mesterolone;Drostanolone;Sideridiol;ent-17-Hydroxy-16beta-kauran-19-al;Yucalexin P21;Copalic acid;Junicedral;7,13-Eperudien-15-oic acid;Oryzalexin S;Oryzalexin E;2,6,10,14-Hexadecatetraenoic acid, 3,7,11,15-tetramethyl-, (Z,E,E)-;5,8,11,14-Icosatetraenoic Acid;Eicosatetraenoic acid;Mestaline;Methandriol;Oryzalexin D;Oryzalexin F;abietadiene-diol;Retinol linoleate | 0.41 | down | 0.01 | 0.54 | 1.85 |
| POS | POS_298.128_2.6795_1 | MMV099637 | C17H16FN3O | Organoheterocyclic compounds | Azoles | Pyrazoles | NA | NA | NA | Phenethylamine glucuronide;Duloxetine;O-Glutarylcarnitine;2-Ethylpropanedioylcarnitine;[(1S,2S,4S,5S)-2-(6-Aminopurin-9-yl)-5-(hydroxymethyl)-4-bicyclo[3.1.0]hexanyl]methanol;Methionyl-Methionine;Gravolenic acid | 0.42 | down | 0.04 | 0.57 | 1.74 |
| POS | POS_401.288_2.987_1 | Arg Leu Ile | C18H36N6O4 | NA | NA | NA | NA | NA | NA | NA | 0.49 | down | 0.04 | 0.57 | 1.30 |
| POS | POS_324.093_0.4953_1 | N-(5-chloro(2-pyridyl))-2-(2-1,2,3,4-tetrahydroisoquinolyl)acetamide | C16H16ClN3O | Organic acids and derivatives | Carboxylic acids and derivatives | Amino acids, peptides, and analogues | NA | NA | NA | Myo-inositol glutamate;Isothipendyl;Leucomethylene blue;Prothipendyl;Flutriafol;1H-1,2,4-Triazole-1-ethanol, alpha,alpha-bis(4-fluorophenyl)-;2-(3,4-Dihydro-2,2-dimethyl-6-nitro-2H-1,4-benzoxazin-4-yl)pyridine N-oxide;3'-Deoxythymidine-5'-monophosphate | 0.73 | down | 0.02 | 0.55 | 1.73 |
| POS | POS_360.183_3.3405_1 | Leu Asn Asn | C14H25N5O6 | NA | NA | NA | NA | NA | NA | 2-[(3,17-Dihydroxy-13-methyl-8,9,11,12,14,15,16,17-octahydro-7H-cyclopenta[a]phenanthren-6-ylidene)amino]oxyacetic acid;Rimcazole;Austrobailignan 7;Hydroxygaleon;7-Hydroxyaustrobailignan 5;1,2,3,4-Tetrahydro-1-[1-hydroxy-3-(4-hydroxyphenyl)-2-propenyl]-7-methoxy-2,6-naphthalenediol | 0.51 | down | 0.00 | 0.48 | 1.61 |
| POS | POS_360.149_3.8585_1 | Pro Glu Asp | C14H21N3O8 | NA | NA | NA | NA | NA | NA | Chlorambucil-tertiary butyl ester;3-[[3-Methoxy-5-(1H-pyrrol-2-yl)pyrrol-2-ylidene]methyl]-1,4-dimethyl-4,5,6,7-tetrahydro-2H-isoindole;Oclacitinib;Cellobiose;D-Maltose;Alpha-Lactose;Sucrose;Lactulose;Trehalose;3-b-Galactopyranosyl glucose;Turanose;Kojibiose;Sakebiose;Inulobiose;Maltulose;Mannobiose;DEAE-cellulose;Allolactose;Glucinol;Trehalulose;Fagopyritol A1;beta-Lactose;Glucose-1,3-mannose oligosaccharide;Polymaltose;laminaribiose;3h-Sucrose;D-Glucose, 4-O-beta-D-galactopyranosyl-;D-Fructose, 6-O-alpha-D-glucopyranosyl-;6-O-alpha-D-Galactopyranosyl-D-galactopyranose;Gal-alpha1,2-Gal;Palatinose;(2R,3S,4R,5R)-2,4,5,6-Tetrahydroxy-3-[(2S,3R,4S,5S,6R)-3,4,5-trihydroxy-6-(hydroxymethyl)oxan-2-yl]oxyhexanal;D-Glucose, 6-O-alpha-D-glucopyranosyl-;(3S,4R,5R)-1,4,5,6-Tetrahydroxy-3-[(2S,3R,4S,5S,6R)-3,4,5-trihydroxy-6-(hydroxymethyl)oxan-2-yl]oxyhexan-2-one;(3S,4R,5S)-3,4,5,6-Tetrahydroxy-1-[(2S,3R,4S,5S,6R)-3,4,5-trihydroxy-6-(hydroxymethyl)oxan-2-yl]oxyhexan-2-one;5-[[5-[[5-(Pyrrol-2-ylidenemethyl)pyrrol-2-ylidene]methyl]-1H-pyrrol-2-yl]methylidene]pyrrole-2-carboxylic acid;beta-D-Fructofuranosyl-(2,1)-beta-D-Fructofuranose | 4.98 | up | 0.01 | 0.53 | 1.83 |
| NEG | NEG_557.459_6.0459_1 | FAHFA 36:4 | C36H62O4 | Lipids and lipid-like molecules | Fatty Acyls | Fatty acid esters | NA | NA | NA | Chatenaytrienin 1;Chatenaytrienin 2;Cholesteryl caprylate | 1.34 | up | 0.04 | 0.57 | 1.46 |
| POS | POS_262.119_2.8417_1 | Tryptophyl-Glycine | C13H15N3O3 | Organic acids and derivatives | Carboxylic acids and derivatives | Amino acids, peptides, and analogues | HMDB0029083 | NA | NA | Glycyl-Tryptophan;Tryptophyl-Glycine;Acetamide, N-[2-(5-methoxy-1-nitroso-1H-indol-3-yl)ethyl]-;Imazapyr;N-[2-(5-Methoxy-1H-indol-3-yl)ethyl]-N-nitrosoacetamide;Dihydrodioscorine;Tigloidine;Dumetorine;2,4-Undecadiene-8,10-diynoic acid 2,3-dehydropiperidide;Nornefopam;Indolylacryloylglycine;Haematopodin;3'-DEOXY-3'-FLUOROTHYMIDINE;Phthalimidinoglutarimide | 0.64 | down | 0.01 | 0.52 | 1.77 |
| POS | POS_150.077_2.7227_1 | 6-Methyladenine | C6H7N5 | Organoheterocyclic compounds | Imidazopyrimidines | Purines and purine derivatives | HMDB0002099 | C08434 | NA | 6-Methyladenine;1-Methyladenine;3-Methyladenine;7-Methyladenine;9-Methyladenine;4-Amino-4-deoxyarabinose;Monoethyl malonic acid;Ethylmalonic acid;Glutaric acid;Methylsuccinic acid;Dimethylmalonic acid;2-Acetolactate;2-C-Methyl-1,4-erythrono-D-lactone;2-Deoxy-L-ribono-1,4-lactone;2-Hydroxy-4-oxopentanoic acid;Monomethyl succinate;Dimethyl malonate;Pteridine;Pyrimido[5,4-d]pyrimidine;Formyl 3-hydroxybutanoate | 0.43 | down | 0.03 | 0.56 | 1.37 |
| NEG | NEG_736.493_5.6348_1 | PE(18:2(9Z,12Z)/18:3(6Z,9Z,12Z)) | C41H72NO8P | Lipids and lipid-like molecules | Glycerophospholipids | Glycerophosphoethanolamines | HMDB0009094 | C00350 | map00563(Glycosylphosphatidylinositol (GPI)-anchor biosynthesis);map00564(Glycerophospholipid metabolism);map01100(Metabolic pathways);map01110(Biosynthesis of secondary metabolites);map04136(Autophagy - other);map04138(Autophagy - yeast);map04140(Autophagy - animal);map04723(Retrograde endocannabinoid signaling);map05130(Pathogenic Escherichia coli infection);map05167(Kaposi sarcoma-associated herpesvirus infection) | PE(14:0/22:5(4Z,7Z,10Z,13Z,16Z));PE(14:0/22:5(7Z,10Z,13Z,16Z,19Z));PE(14:1(9Z)/22:4(7Z,10Z,13Z,16Z));PE(16:0/20:5(5Z,8Z,11Z,14Z,17Z));PE(16:1(9Z)/20:4(5Z,8Z,11Z,14Z));PE(16:1(9Z)/20:4(8Z,11Z,14Z,17Z));PE(18:1(11Z)/18:4(6Z,9Z,12Z,15Z));PE(18:1(9Z)/18:4(6Z,9Z,12Z,15Z));PE(18:2(9Z,12Z)/18:3(6Z,9Z,12Z));PE(18:2(9Z,12Z)/18:3(9Z,12Z,15Z));PE(18:3(6Z,9Z,12Z)/18:2(9Z,12Z));PE(18:3(9Z,12Z,15Z)/18:2(9Z,12Z));PE(18:4(6Z,9Z,12Z,15Z)/18:1(11Z));PE(18:4(6Z,9Z,12Z,15Z)/18:1(9Z));PE(20:4(5Z,8Z,11Z,14Z)/16:1(9Z));PE(20:4(8Z,11Z,14Z,17Z)/16:1(9Z));PE(20:5(5Z,8Z,11Z,14Z,17Z)/16:0);PE(22:4(7Z,10Z,13Z,16Z)/14:1(9Z));PE(22:5(4Z,7Z,10Z,13Z,16Z)/14:0);PE(22:5(7Z,10Z,13Z,16Z,19Z)/14:0);Ternatin;PE-NMe(15:0/20:5(5Z,8Z,11Z,14Z,17Z));PE-NMe(20:5(5Z,8Z,11Z,14Z,17Z)/15:0);PE-NMe2(14:0/20:5(5Z,8Z,11Z,14Z,17Z));PE-NMe2(14:1(9Z)/20:4(5Z,8Z,11Z,14Z));PE-NMe2(14:1(9Z)/20:4(8Z,11Z,14Z,17Z));PE-NMe2(16:1(9Z)/18:4(6Z,9Z,12Z,15Z));PE-NMe2(18:4(6Z,9Z,12Z,15Z)/16:1(9Z));PE-NMe2(20:4(5Z,8Z,11Z,14Z)/14:1(9Z));PE-NMe2(20:4(8Z,11Z,14Z,17Z)/14:1(9Z));PE-NMe2(20:5(5Z,8Z,11Z,14Z,17Z)/14:0);PE(P-16:0/20:4(6E,8Z,11Z,14Z)+=O(5));PE(20:4(6E,8Z,11Z,14Z)+=O(5)/P-16:0);PE(P-16:0/20:4(5Z,8Z,11Z,13E)+=O(15));PE(20:4(5Z,8Z,11Z,13E)+=O(15)/P-16:0);PE(P-16:0/20:5(5Z,8Z,11Z,14Z,16E)-OH(18R));PE(20:5(5Z,8Z,11Z,14Z,16E)-OH(18R)/P-16:0);PE(P-16:0/20:5(5Z,8Z,11Z,14Z,16E)-OH(18));PE(20:5(5Z,8Z,11Z,14Z,16E)-OH(18)/P-16:0);PE(P-16:0/20:5(5Z,8Z,10E,14Z,17Z)-OH(12));PE(20:5(5Z,8Z,10E,14Z,17Z)-OH(12)/P-16:0);PE(P-16:0/20:5(6E,8Z,11Z,14Z,17Z)-OH(5));PE(20:5(6E,8Z,11Z,14Z,17Z)-OH(5)/P-16:0) | 0.33 | down | 0.01 | 0.53 | 1.88 |
| POS | POS_277.068_0.6152_1 | O-Demethylfonsecin | C14H12O6 | Organoheterocyclic compounds | Naphthopyrans | Naphthopyranones | HMDB0033649 | NA | NA | 4-Hydroxybenzoic acid;Salicylic acid;3-Hydroxybenzoic acid;Gentisate aldehyde;2-Methoxy-1,4-benzoquinone;alpha-Furyl methyl diketone;Sesamol;3,4-Dihydroxybenzaldehyde;keratan sulfate II (core 2-linked), degradation product 1;3,6-Dihydroxycyclohepta-2,4,6-trien-1-one;O-Demethylfonsecin;1-(3-Fluoro-4-hydroxy-5-mercaptomethyltetrahydrofuran-2-yl)-5-methylpyrimidine-2,4(1H,3H)-dione;Biotin thiamine;(x)-1,2-Propanediol 1-O-b-D-glucopyranoside;Proxyphylline;[(2S,3S,4R,5R)-3,4,5,6-Tetrahydroxyhexan-2-yl] 2-hydroxypropanoate;L-Phosphoarginine;2'-Fluoro-2',3'-dideoxyinosine;Bis(2-hydroxyethyl) terephthalate;Glucosamine 6-sulfate;N-Sulfo-D-glucosamine | 0.14 | down | 0.04 | 0.57 | 1.64 |
| POS | POS_776.231_8.9867_1 | Contaminant vial septum ThermoFisher C5000-44B | NA | NA | NA | NA | NA | NA | NA | Mertansine | 1.62 | up | 0.02 | 0.54 | 1.22 |
| POS | POS_271.226_4.8775_1 | 16-Hydroxypalmitic acid | C16H32O3 | Lipids and lipid-like molecules | Fatty Acyls | Fatty acids and conjugates | NA | C18218 | map00073(Cutin, suberine and wax biosynthesis);map01100(Metabolic pathways) | 3-Oxohexadecanoic acid;8-Oxohexadecanoic acid;9-Oxohexadecanoic acid;11-Oxohexadecanoic acid;2-Hydroxydodecyl methacrylate | 1.88 | up | 0.01 | 0.53 | 1.44 |
| POS | POS_415.212_4.2749_1 | Benzoic acid, 4-hydroxy-, (1aS,2aR,3S,5R,6S,7aR)-3-(acetyloxy)decahydro-5-hydroxy-2a,7a-dimethyl-5-(1-methylethyl)azuleno[5,6-b]oxiren-6-yl ester | C24H32O7 | Benzenoids | Benzene and substituted derivatives | Benzoic acids and derivatives | NA | NA | NA | 4-(Methylnitrosamino)-1-(3-pyridyl)-1-butanone;4-Carbamimidoyl-L-phenylalanine;M-Amidinophenyl-3-alanine;4'-(Nitrosomethylamino)-1-(3-pyridyl)-1-butanone;(2S)-2-(Diaminomethylideneamino)-3-phenylpropanoic acid;Eplerenone;Armillaripin;4-O-Methylmelleolide;Armillarin;Clausarinol;N-Palmitoyl Arginine;Trospium;Dihydrofukinolide;10-Hydroperoxy-H4-neuroprostane;11-Hydroperoxy-H4-neuroprostane;14-Hydroperoxy-H4-neuroprostane;N,N'-Dibenzhydrylethane-1,2-diamine;N'-[(6-Oxo-5-prop-2-enyl-1-cyclohexa-2,4-dienylidene)methyl]-2-[4-(phenylmethyl)-1-piperazinyl]acetohydrazide;Reduced ketanserin | 2.29 | up | 0.01 | 0.53 | 1.89 |
| POS | POS_291.272_3.8441_1 | Cis-11-Eicosenoic acid | C20H38O2 | Lipids and lipid-like molecules | Fatty Acyls | Fatty acids and conjugates | NA | C16526 | map01040(Biosynthesis of unsaturated fatty acids) | NA | 1.42 | up | 0.02 | 0.56 | 1.08 |
| NEG | NEG_377.198_3.55_1 | 11-Dihydro-12-norneoquassin | C21H30O6 | Lipids and lipid-like molecules | Prenol lipids | Polyprenols | HMDB0036889 | NA | NA | 18-Hydroxycortisol;Pentosidine;Isohumulinone A;Humulinone;Eremopetasitenin C1;Eremopetasitenin D1;11-Dihydro-12-norneoquassin;Sugetriol triacetate;6-beta-hydrocortisol;6-Hydroxycortisol;(2E)-2-[(4,5-Dimethoxy-2-methyl-3,6-dioxocyclohexa-1,4-dien-1-yl)methylidene]undecanoic acid;hydroxycortisol;3-(2-Aminoethyl)-5-methoxy-N-[2-(5-methoxy-1H-indol-3-yl)ethyl]indol-1-amine;Pregn-4-ene-3,20-dione, 11,17,18,21-tetrahydroxy-, (11beta)-;(8S,9S,10S,11S,13S,14S,17R)-11,17-Dihydroxy-17-(2-hydroxyacetyl)-10,13-dimethyl-1,4,5,6,7,8,9,11,12,14,15,16-dodecahydrocyclopenta[a]phenanthrene-2,3-dione;2-[4-(Carboxymethyl)-1,4,8,11-tetrazabicyclo[6.6.2]hexadecan-11-yl]acetic acid;Carnosic acid;7'-Carboxy-gamma-tocotrienol;Hulupone;Adhulupone;(1(10)E,4E,6a,9b)-9-(2-Methylbutanoyloxy)-1(10),4,11(13)-germacratrien-12,6-olide;(1(10)E,4E,6a,9b)-9-(3-Methylbutanoyloxy)-1(10),4,11(13)-germacratrien-12,6-olide;Petasitin;6-Angeloylfuranofukinol;4-Deoxycohumulone;ent-7alpha,12beta-Dihydroxy-16-kauren-19,6beta-olide;ent-15-Kaurene-17,19-dioic acid;7,18-Dihydroxykaurenolide;(9Z,11S,16S)-1-Acetoxy-9,17-octadecadiene-12,14-diyne-11,16-diol;5,6-epoxy,18R-HEPE;14-Deoxy-11,12-didehydroandrographolide;Glaucocalyxin A;Dehydroandrographolide;Gibberellin A116;Horminone;19-Hydroxyroyleanone;Gibberellin A12;11b-Hydroxyprogesterone;Ubiquinone-2;Cohulupone;(1(10)E,4E,6a,9b)-9-(2-Methylpropanoyloxy)-1(10),4,11(13)-germacratrien-12,6-olide;Panaquinquecol 6;[8]-Dehydrogingerdione;Ubiquinones;2-Methoxyestriol;1-Dehydro-[8]-gingerdione | 0.48 | down | 0.01 | 0.53 | 1.58 |
| NEG | NEG_265.13_3.6139_1 | 3-Phenylpropyl cinnamate | C18H18O2 | Phenylpropanoids and polyketides | Cinnamic acids and derivatives | Cinnamic acid esters | HMDB0036387 | NA | NA | Isoleucyl-Valine;Leucyl-Valine;Lidamidine | 0.10 | down | 0.01 | 0.52 | 3.23 |
| NEG | NEG_815.497_5.982_1 | PG(20:4(6Z,8E,10E,14Z)-2OH(5S,12R)/i-17:0) | C43H77O12P | Lipids and lipid-like molecules | Glycerophospholipids | Glycerophosphoglycerols | HMDB0271605 | C00344 | map00552(Teichoic acid biosynthesis);map00564(Glycerophospholipid metabolism);map01100(Metabolic pathways) | Arginyl-arginyl-prolyl-tyrosyl-isoleucyl-leucine;PA(21:0/20:4(6E,8Z,11Z,14Z)+=O(5));PA(20:4(6E,8Z,11Z,14Z)+=O(5)/21:0);PA(21:0/20:4(5Z,8Z,11Z,13E)+=O(15));PA(20:4(5Z,8Z,11Z,13E)+=O(15)/21:0);PA(21:0/20:5(5Z,8Z,11Z,14Z,16E)-OH(18R));PA(20:5(5Z,8Z,11Z,14Z,16E)-OH(18R)/21:0);PA(21:0/20:5(5Z,8Z,11Z,14Z,16E)-OH(18));PA(20:5(5Z,8Z,11Z,14Z,16E)-OH(18)/21:0);PA(21:0/20:5(5Z,8Z,10E,14Z,17Z)-OH(12));PA(20:5(5Z,8Z,10E,14Z,17Z)-OH(12)/21:0);PA(21:0/20:5(6E,8Z,11Z,14Z,17Z)-OH(5));PA(20:5(6E,8Z,11Z,14Z,17Z)-OH(5)/21:0);PA(a-21:0/20:4(6E,8Z,11Z,14Z)+=O(5));PA(20:4(6E,8Z,11Z,14Z)+=O(5)/a-21:0);PA(a-21:0/20:4(5Z,8Z,11Z,13E)+=O(15));PA(20:4(5Z,8Z,11Z,13E)+=O(15)/a-21:0);PA(a-21:0/20:5(5Z,8Z,11Z,14Z,16E)-OH(18R));PA(20:5(5Z,8Z,11Z,14Z,16E)-OH(18R)/a-21:0);PA(a-21:0/20:5(5Z,8Z,11Z,14Z,16E)-OH(18));PA(20:5(5Z,8Z,11Z,14Z,16E)-OH(18)/a-21:0);PA(a-21:0/20:5(5Z,8Z,10E,14Z,17Z)-OH(12));PA(20:5(5Z,8Z,10E,14Z,17Z)-OH(12)/a-21:0);PA(a-21:0/20:5(6E,8Z,11Z,14Z,17Z)-OH(5));PA(20:5(6E,8Z,11Z,14Z,17Z)-OH(5)/a-21:0);PA(i-21:0/20:4(6E,8Z,11Z,14Z)+=O(5));PA(20:4(6E,8Z,11Z,14Z)+=O(5)/i-21:0);PA(i-21:0/20:4(5Z,8Z,11Z,13E)+=O(15));PA(20:4(5Z,8Z,11Z,13E)+=O(15)/i-21:0);PA(i-21:0/20:5(5Z,8Z,11Z,14Z,16E)-OH(18R));PA(20:5(5Z,8Z,11Z,14Z,16E)-OH(18R)/i-21:0);PA(i-21:0/20:5(5Z,8Z,11Z,14Z,16E)-OH(18));PA(20:5(5Z,8Z,11Z,14Z,16E)-OH(18)/i-21:0);PA(i-21:0/20:5(5Z,8Z,10E,14Z,17Z)-OH(12));PA(20:5(5Z,8Z,10E,14Z,17Z)-OH(12)/i-21:0);PA(i-21:0/20:5(6E,8Z,11Z,14Z,17Z)-OH(5));PA(20:5(6E,8Z,11Z,14Z,17Z)-OH(5)/i-21:0);MG(PGJ2/0:0/0:0);MG(0:0/PGJ2/0:0);[8]-Gingerdiol | 0.27 | down | 0.02 | 0.56 | 2.02 |
| POS | POS_243.087_3.3855_1 | 2-amino-3-(5-hydroxy-1H-indol-3-yl)propanoic acid | C19H22N2O | Alkaloids and derivatives | Eburnan-type alkaloids | NA | NA | NA | NA | Imidazotriazine;pyrazolotriazine;Genipinic acid;Elenaic acid;Lumichrome;Bufotenin;Psilocin;Nebracetam;(+)-gamma-Hydroxy-L-homoarginine;2,4-Diamino-6,7-dimethoxyquinazoline | 1.99 | up | 0.03 | 0.56 | 1.26 |
| NEG | NEG_712.501_8.3001_1 | PE(18:3(9Z,12Z,15Z)/16:0) | C39H72NO8P | Lipids and lipid-like molecules | Glycerophospholipids | Glycerophosphoethanolamines | HMDB0009154 | C00350 | map00563(Glycosylphosphatidylinositol (GPI)-anchor biosynthesis);map00564(Glycerophospholipid metabolism);map01100(Metabolic pathways);map01110(Biosynthesis of secondary metabolites);map04136(Autophagy - other);map04138(Autophagy - yeast);map04140(Autophagy - animal);map04723(Retrograde endocannabinoid signaling);map05130(Pathogenic Escherichia coli infection);map05167(Kaposi sarcoma-associated herpesvirus infection) | NA | 0.35 | down | 0.01 | 0.53 | 1.94 |
| POS | POS_275.277_4.3604_1 | Digeranyl | C20H34 | Lipids and lipid-like molecules | Prenol lipids | Diterpenoids | HMDB0035152 | NA | NA | NA | 1.44 | up | 0.02 | 0.56 | 1.40 |
| NEG | NEG_165.041_0.606_1 | Xylan | C5H10O6 | Organic oxygen compounds | Organooxygen compounds | Carbohydrates and carbohydrate conjugates | HMDB0301774 | C00707 | map04973(Carbohydrate digestion and absorption) | Arabinonic acid;Ribonic acid;3-Methylxanthine;7-Methylxanthine;1-Methylxanthine;S-Propyl 1-propanesulfinothioate;9-Methylxanthine;D-Xylonic acid;L-Lyxonic acid;L-Xylonic acid;Xylan;(S)-3,4-Dihydroxybutyric acid;2,4-Dihydroxybutanoic acid;4-Deoxyerythronic acid;Purine;4-Deoxythreonic acid;A,b-Dihydroxyisobutyric acid;Erythrose;L-Erythrulose;(2-Hydroxyethoxy)acetic acid;1H-Pyrazolo[4,3-d]pyrimidine;2,3-Dihydroxybutanoic acid;5-Aminopyrimidine-2-carbonitrile;D-Erythrose;1,3-Dihydroxypropan-2-yl formate;pyrazolopyridazine;L-Glyceric acid;Propaneperoxoic acid, 2-hydroxy-;2-(Hydroxymethoxy)acetic acid | 0.38 | down | 0.03 | 0.56 | 1.65 |
| NEG | NEG_560.477_7.6378_1 | Cer(d17:1/18:3(10,12,15)-OH(9)) | C35H63NO4 | Lipids and lipid-like molecules | Sphingolipids | Ceramides | HMDB0289933 | C00195 | map00600(Sphingolipid metabolism);map01100(Metabolic pathways);map04071(Sphingolipid signaling pathway);map04217(Necroptosis);map04722(Neurotrophin signaling pathway);map04920(Adipocytokine signaling pathway);map04931(Insulin resistance);map04933(AGE-RAGE signaling pathway in diabetic complications);map05140(Leishmaniasis);map05415(Diabetic cardiomyopathy) | NA | 1.41 | up | 0.04 | 0.56 | 1.23 |
| NEG | NEG_447.135_3.4724_1 | Aromadendrin 4'-methyl ether 7-rhamnoside | C22H24O10 | Phenylpropanoids and polyketides | Flavonoids | Flavonoid glycosides | HMDB0040561 | NA | NA | Pamicogrel;Dicaffeoylputrescine;Fluocinolone;Propyl 1-(propylthio)propyl disulfide;Rafabegron;Setipiprant;Sulfinpyrazone sulfide;Fipexide | 3.41 | up | 0.00 | 0.48 | 2.03 |
| POS | POS_294.154_1.1365_1 | Glucuronic_acid,_8CI,_9CI_6-Aminohexyl_glycoside | C12H23NO7 | Lipids and lipid-like molecules | Fatty Acyls | Fatty acyl glycosides | NA | NA | NA | N-(1-Deoxy-1-fructosyl)leucine;N-(1-Deoxy-1-fructosyl)isoleucine;3,4,5-Trihydroxypentanoylcarnitine;Arginylproline;Prolyl-Arginine;Triethyl citrate | 0.60 | down | 0.02 | 0.56 | 2.21 |
| POS | POS_286.176_2.8384_1 | Gly Pro Ile | C13H23N3O4 | NA | NA | NA | NA | NA | NA | N-Desmethylvenlafaxine;Desvenlafaxine;Hydroxy-alpha-sanshool;6-(Dipropylamino)-5,6,7,8-tetrahydronaphthalene-1,2-diol;3-Amino-1-(3-(cyclohexylmethoxy)phenyl)propan-1-ol;Ultram;6-(Dipropylamino)-5,6,7,8-tetrahydronaphthalene-2,3-diol;4-Bis(2-hydroxyethyl)amino-L-phenylalanine | 0.41 | down | 0.02 | 0.54 | 1.49 |
| NEG | NEG_383.353_7.3546_1 | Cerebronic acid | C24H48O3 | Lipids and lipid-like molecules | Fatty Acyls | Fatty acids and conjugates | HMDB0039540 | C17873 | NA | Cerebronic acid;24-Hydroxytetracosanoic acid;Tricosanal;Docosanal | 1.68 | up | 0.02 | 0.56 | 1.54 |
| POS | POS_379.296_4.4254_1 | Ergosterol | C28H44O | Lipids and lipid-like molecules | Steroids and steroid derivatives | Ergostane steroids | HMDB0000878 | C01694 | map00100(Steroid biosynthesis);map01100(Metabolic pathways);map01110(Biosynthesis of secondary metabolites) | 6-Hydroxy-8-docosanone;8-Hydroxy-6-docosanone;5-Hydroxy-7-docosanone;4-Hydroxy-6-docosanone;Butyl octadecanoate;Isobehenic acid;N-Myristoyl Lysine;6-(6-(3r-Hydroxy-1e,5z-undecadien-1-yl)-2-pyridinyl)-1,5s-hexanediol | 0.53 | down | 0.00 | 0.52 | 1.69 |
| POS | POS_288.192_2.9118_1 | Gly Val Ile | C13H25N3O4 | NA | NA | NA | NA | NA | NA | Mebeverine alcohol | 0.56 | down | 0.03 | 0.56 | 1.26 |
| POS | POS_450.32_4.9621_1 | Glycochenodeoxycholic acid | C26H43NO5 | Lipids and lipid-like molecules | Steroids and steroid derivatives | Bile acids, alcohols and derivatives | NA | C05466 | map00120(Primary bile acid biosynthesis);map00121(Secondary bile acid biosynthesis);map04976(Bile secretion);map04979(Cholesterol metabolism) | Deoxycholic acid glycine conjugate;Chenodeoxyglycocholic acid;Chenodeoxycholylglycine;Deoxycholylglycine;Glycohyodeoxycholic acid;Latanoprost;7-[(1R,2R,3R,5S)-3,5-Dihydroxy-2-[(3R)-3-hydroxy-5-phenylpentyl]cyclopentyl]-5-heptenoic acid propan-2-yl ester | 0.13 | down | 0.05 | 0.57 | 1.75 |
| POS | POS_432.237_4.2724_1 | N-(2-(((1S,9aR)-octahydro-1H-quinolizin-1-yl)methyl)-3-oxo-1,2,3,4-tetrahydrobenzo[4,5]imidazo[1,2-a]pyrazin-8-yl)propionamide | C23H31N5O2 | Organoheterocyclic compounds | Quinolizines | NA | NA | NA | NA | Tropolone A;undecylprodigiosin;(3E,5Z,11Z)-Pentadeca-3,5,11-trienedioylcarnitine;Pentadeca-9,11,13-trienedioylcarnitine;Pentadeca-3,6,9-trienedioylcarnitine;Pentadeca-7,10,13-trienedioylcarnitine;Pentadeca-4,6,8-trienedioylcarnitine;Pentadeca-5,8,11-trienedioylcarnitine;(2E,6E,10E)-Pentadeca-2,6,10-trienedioylcarnitine;Pentadeca-5,7,9-trienedioylcarnitine;Pentadeca-3,5,7-trienedioylcarnitine;Eplerenone;Armillaripin;4-O-Methylmelleolide;Armillarin;Clausarinol | 2.19 | up | 0.01 | 0.52 | 1.73 |
| POS | POS_207.149_3.01_1 | Agrocybenine | C12H18N2O | Organoheterocyclic compounds | Pyrrolopyridines | NA | HMDB0041445 | NA | NA | Agrocybenine;Monoethylglycinexylidide;N-Hydroxy-N'-(4-butyl-2-methylphenyl)formamidine;ISOPROTURON;Oxotremorine;2-(Ethylamino)-N-methyl-N-(p-tolyl)acetamide;(+/-)-N,N-Dimethyl menthyl succinamide;1-Dodecene;(E)-2-Dodecene;(Z)-3-Dodecene;(E)-3-Dodecene;4,6,8-trimethyl-1-nonene;2,2,4,6,6-Pentamethyl-3-heptene;Nonyl cyclopropane;3-Methyl-2-undecene (Z);Cyclododecane;1-(Benzofuran-5-yl)-N-Methylpropan-2-aMine;2-(Dimethylaminomethyl)-1-phenyl-prop-2-en-1-one;Phenyl(piperidin-4-yl)methanone | 7.03 | up | 0.05 | 0.57 | 1.53 |
| NEG | NEG_283.265_3.3623_1 | Stearic acid | C18H36O2 | Lipids and lipid-like molecules | Fatty Acyls | Fatty acids and conjugates | HMDB0000827 | C01530 | map00061(Fatty acid biosynthesis);map01040(Biosynthesis of unsaturated fatty acids);map01060(Biosynthesis of plant secondary metabolites);map01100(Metabolic pathways);map04745(Phototransduction - fly) | Stearic acid;Ethyl hexadecanoate;16-Methylheptadecanoic acid;Hexyl dodecanoate;2-Octadecanone, 3-hydroxy-;Dihydroisophorol;(Z)-3-Nonen-1-ol;2-Nonanone;3-Nonanone;Amyl propyl ketone;2-Methyloctanal;3,5,5-Trimethylhexanal;1-Nonen-3-ol;(&#177;)-2,6-Dimethyl-6-hepten-1-ol;(Z)-6-Nonen-1-ol;2-Nonen-1-ol;(Z)-2-Nonen-1-ol;(E)-3-Nonen-1-ol;Nonanal;(E)-6-Nonenol;5-Nonanone;Menotrophin;1-Heptadecene;7-heptadecene;1-Hexadecene | 1.51 | up | 0.04 | 0.57 | 1.47 |
| POS | POS_152.057_1.0041_1 | 3-Methoxybenzoic acid | C8H8O3 | Benzenoids | Benzene and substituted derivatives | Benzoic acids and derivatives | HMDB0032606 | NA | NA | Guanine;2-Hydroxyadenine;8-Hydroxyadenine;8-Oxo-7,8-dihydrodeoxyguanine;(S)-2-Amino-4-methylsulfanyl-butane-1-thiol;3,3-Dimethyl-1,2-dithiolane;Tetrahydro-2-methyl-2-thiophenethiol;Tetrahydro-2-methyl-3-thiophenethiol;Pyrazolopyrimidinone;1,2-Dimercaptocyclopentane | 0.40 | down | 0.05 | 0.57 | 1.39 |
| POS | POS_211.087_3.0376_1 | Bancroftinone | C11H14O4 | Organic oxygen compounds | Organooxygen compounds | Carbonyl compounds | HMDB0041406 | NA | NA | 1-Methoxyphenazine;2-Aminoacridone;Pyocyanin;C1=CC=CC2=CC3=C(O)C(N)=CC=C3N=C21;1,2-Dihydro-1,1,6-trimethylnaphthalene;6-Fluoro-8-methoxy quinolone;3-Fluoro-8-methoxy-1H-quinolin-2-one | 9.86 | up | 0.04 | 0.56 | 1.52 |
| POS | POS_381.08_0.8714_1 | Melibiose | C12H22O11 | Organic oxygen compounds | Organic oxygen compounds | Carbohydrates and carbohydrate conjugates | HMDB0000048 | C05402 | map00052(Galactose metabolism);map01100(Metabolic pathways);map02010(ABC transporters) | Nitroxoline;4-Nitroquinoline N-oxide;Piclamilast;Cellobiose;D-Maltose;Alpha-Lactose;Sucrose;Lactulose;Trehalose;3-b-Galactopyranosyl glucose;Turanose;Kojibiose;Sakebiose;Inulobiose;Maltulose;Mannobiose;DEAE-cellulose;Allolactose;Glucinol;Trehalulose;Fagopyritol A1;beta-Lactose;Glucose-1,3-mannose oligosaccharide;Polymaltose;laminaribiose;3h-Sucrose;D-Glucose, 4-O-beta-D-galactopyranosyl-;D-Fructose, 6-O-alpha-D-glucopyranosyl-;6-O-alpha-D-Galactopyranosyl-D-galactopyranose;Gal-alpha1,2-Gal;Palatinose;(2R,3S,4R,5R)-2,4,5,6-Tetrahydroxy-3-[(2S,3R,4S,5S,6R)-3,4,5-trihydroxy-6-(hydroxymethyl)oxan-2-yl]oxyhexanal;D-Glucose, 6-O-alpha-D-glucopyranosyl-;(3S,4R,5R)-1,4,5,6-Tetrahydroxy-3-[(2S,3R,4S,5S,6R)-3,4,5-trihydroxy-6-(hydroxymethyl)oxan-2-yl]oxyhexan-2-one;(3S,4R,5S)-3,4,5,6-Tetrahydroxy-1-[(2S,3R,4S,5S,6R)-3,4,5-trihydroxy-6-(hydroxymethyl)oxan-2-yl]oxyhexan-2-one;beta-D-Fructofuranosyl-(2,1)-beta-D-Fructofuranose;N1-(5-Phospho-a-D-ribosyl)-5,6-dimethylbenzimidazole;Dihydrocaffeic acid 3-O-glucuronide;6-[3-(2-carboxyethyl)-5-hydroxyphenoxy]-3,4,5-trihydroxyoxane-2-carboxylic acid;Homovanillic acid 4-glucuronide;4-(4,5-Diphenyl-1H-imidazol-2-yl)benzoyl Chloride;7-(2-Chloro-6-fluorophenyl)-5-(4-chlorophenyl)-1,5,6,7-tetrahydrotetrazolo[1,5-a]pyrimidine | 0.37 | down | 0.03 | 0.56 | 1.58 |
| NEG | NEG_155.047_1.1299_1 | 5-(2-carboxylatoethyl)-4-oxo-4,5-dihydro-1H-imidazol-5-ide | C6H8N2O3 | Organoheterocyclic compounds | Azoles | Imidazoles | HMDB0062719 | NA | NA | 5-Hydroxymethyl-4-methyluracil;4-Imidazolone-5-propionic acid;Imidazolelactic acid;5-(2-carboxylatoethyl)-4-oxo-4,5-dihydro-1H-imidazol-5-ide;1,5-Dimethylbarbituric acid;2-Hydroxy-3-(1H-imidazol-5-yl)propanoic acid;1,3,4-Pentanetriol;2-(2-Methoxyethoxy)ethanol;Trimethyl orthoacetate;Imidazole-4-acetaldehyde;Methoxypyrazine;3-Hydroxy-4-aminopyridine;N-Acetylimidazole;2-Furancarboximidamide;2-Hydroxypyrazine;3(2H)-Pyridazinone;5-Hydroxypyrimidine;pyrimidin-2-ol;pyrimidin-4-ol | 0.44 | down | 0.05 | 0.57 | 1.41 |
| POS | POS_267.052_1.9648_1 | Oxyresveratrol | C14H12O4 | Phenylpropanoids and polyketides | Stilbenes | NA | NA | C10273 | NA | 2-O-p-Coumaroyltartronic acid;4-[3-(4-Chlorophenyl)-1,2,4-oxadiazol-5-yl]butanoic acid;Echinochrome A;3-[4-(1-Imidazolylmethyl)phenyl]-2-propenoic acid;Alendronic acid | 0.78 | down | 0.03 | 0.56 | 1.67 |
| POS | POS_344.315_5.6426_1 | Dihydroceramide C2 | C20H41NO3 | NA | NA | NA | NA | NA | NA | N-Acetylsphinganine | 2.13 | up | 0.00 | 0.48 | 1.84 |
| NEG | NEG_135.045_4.4021_1 | 2-Hydroxy-4-methylbenzaldehyde | C8H8O2 | Organic oxygen compounds | Organooxygen compounds | Carbonyl compounds | HMDB0032603 | NA | NA | Phenylacetic acid;2-Methylbenzoic acid;4-Hydroxyphenylacetaldehyde;4-Methylbenzoic acid;4-Methoxybenzaldehyde;3-Methoxybenzaldehyde;3-(5-Methyl-2-furyl)prop-2-enal;2'-Hydroxyacetophenone;2-Hydroxy-4-methylbenzaldehyde;4-(2-Furanyl)-3-buten-2-one;2-Methoxybenzaldehyde;Methyl benzoate;alpha-Methyl-2-furanacrolein;3-(3-Furanyl)-2-methyl-2-propenal;Phenyl acetate;Benzyl formate;M-toluic Acid;1,4-Benzodioxane;2-Hydroxyacetophenone;Oxonol;3-Butyn-1-al;Furan;Cyclohexa-1,2,4,5-tetraene | 2.07 | up | 0.01 | 0.53 | 1.97 |
| POS | POS_336.326_8.3537_1 | N-Isobutyloctadeca-trans-2-trans-4-dienamide | C22H41NO | Lipids and lipid-like molecules | Fatty Acyls | Fatty amides | HMDB0302880 | NA | NA | Pipericine;N-Isobutyloctadeca-trans-2-trans-4-dienamide | 1.76 | up | 0.00 | 0.48 | 1.68 |
| POS | POS_512.503_8.1085_1 | Cer(d18:0/14:0) | C32H66N1O3 | Lipids and lipid-like molecules | Sphingolipids | Ceramides | HMDB0011759 | NA | NA | Cer(d18:0/14:0) | 1.40 | up | 0.02 | 0.55 | 1.07 |
| NEG | NEG_509.258_3.4923_1 | Physagulin A | C30H38O7 | Organoheterocyclic compounds | Quinolines and derivatives | Benzoquinolines | HMDB0041047 | NA | NA | Physagulin A;Vanoxerine | 0.60 | down | 0.01 | 0.54 | 1.49 |
| NEG | NEG_233.064_1.0055_1 | 1-Isopropyl citrate | C9H14O7 | Organic acids and derivatives | Carboxylic acids and derivatives | Tricarboxylic acids and derivatives | HMDB0032438 | NA | NA | NA | 0.67 | down | 0.00 | 0.48 | 2.23 |
| NEG | NEG_166.018_0.6754_1 | Quinolinic acid | C7H5NO4 | Organoheterocyclic compounds | Pyridines and derivatives | Pyridinecarboxylic acids and derivatives | HMDB0000232 | C03722 | map00380(Tryptophan metabolism);map00410(beta-Alanine metabolism);map00760(Nicotinate and nicotinamide metabolism);map01060(Biosynthesis of plant secondary metabolites);map01064(Biosynthesis of alkaloids derived from ornithine, lysine and nicotinic acid);map01100(Metabolic pathways);map01240(Biosynthesis of cofactors) | Homocysteinesulfinic acid;Thioguanine;N-Acetyltaurine;6-Chlorohexanenitrile;1,2,3-Benzotriazine;PYRIDOPYRIMIDINE;pyrrolodiazepine;L-Cysteine;D-Cysteine;DL-Cysteine;S-Nitrosomercaptoethanol | 1.56 | up | 0.02 | 0.56 | 2.23 |
| POS | POS_360.199_3.4512_1 | Pro Phe Pro | C19H25N3O4 | NA | NA | NA | NA | NA | NA | Norendoxifen;5-Hydroxydec-6-enedioylcarnitine;5-Hydroxydec-7-enedioylcarnitine;5-Hydroxydec-8-enedioylcarnitine;6-Hydroxydec-6-enedioylcarnitine;5-Hydroxydec-5-enedioylcarnitine;(2Z)-5-Hydroxydec-2-enedioylcarnitine;6-Hydroxydec-7-enedioylcarnitine;Jasmolone glucoside | 0.77 | down | 0.04 | 0.57 | 1.21 |
| NEG | NEG_251.079_1.8158_1 | 2'-Deoxyinosine | C10H12N4O4 | Nucleosides, nucleotides, and analogues | Purine nucleosides | Purine 2'-deoxyribonucleosides | HMDB0000071 | C05512 | map00230(Purine metabolism);map01100(Metabolic pathways);map01232(Nucleotide metabolism);map02010(ABC transporters) | Deoxyinosine;Nebularine;4-Hydroxyvalerylcarnitine;4-(3-Phosphonopropyl)piperazine-2-carboxylic acid;3'-Deoxyinosine;Cndac;Glucose propionate;[(2R,3S,4R,5R)-3,4,5,6-Tetrahydroxy-1-oxohexan-2-yl] 2-hydroxypropanoate;Thymine dimer;Prolyl-Threonine;Sakacin P;2,5-Bis(acetylamino)pentanoic acid;[(2S,3R)-2-Amino-3-hydroxybutanoyl] (2S)-pyrrolidine-2-carboxylate;Thymine;Imidazoleacetic acid;1H-Imidazole-1-acetic acid;5-Methyl-1H-pyrazole-3-carboxylic acid;6-Methyluracil;Diethyl tartrate;2,4,6-Triethyl-1,3,5-oxadithiane;4,6-O-Ethylidene-alpha-D-glucose;4,6-O-Ethylidene-D-glucose;6,8-Bis(sulfanyl)octanal;Isopropyl tartaric acid | 0.29 | down | 0.02 | 0.56 | 1.52 |
| POS | POS_123.061_2.1784_1 | Nicotinamide | C6H6N2O | Organoheterocyclic compounds | Pyridines and derivatives | Pyridinecarboxylic acids and derivatives | NA | C00153 | map00760(Nicotinate and nicotinamide metabolism);map01100(Metabolic pathways);map01240(Biosynthesis of cofactors);map04212(Longevity regulating pathway - worm);map04977(Vitamin digestion and absorption) | NA | 1.61 | up | 0.01 | 0.53 | 1.21 |
| POS | POS_369.347_5.6316_1 | Lathosterol | C27H46O | Lipids and lipid-like molecules | Steroids and steroid derivatives | Cholestane steroids | HMDB0001170 | C01189 | map00100(Steroid biosynthesis);map01100(Metabolic pathways);map01110(Biosynthesis of secondary metabolites) | NA | 0.46 | down | 0.04 | 0.57 | 1.34 |
| POS | POS_162.058_1.0369_1 | 2,8-Quinolinediol | C9H7NO2 | Organoheterocyclic compounds | Quinolines and derivatives | Quinolones and derivatives | HMDB0240311 | C06342 | NA | trans-S-(1-Propenyl)-L-cysteine;S-Allylcysteine;Allyl cysteine;Cysteine allyl ester;S-Propenylcysteine;S-Ethyl 2-acetylaminoethanethioate;5-Hydroxy-2,4-dimethylthiophen-3-one | 0.79 | down | 0.04 | 0.56 | 1.47 |
| POS | POS_175.001_1.0219_1 | L-Theanine | C7H14N2O3 | Organic acids and derivatives | Carboxylic acids and derivatives | Amino acids, peptides, and analogues | HMDB0034365 | C01047 | NA | Hypoxanthine;Erythronic acid;Threonic acid;Allopurinol;1-Pentanesulfenothioic acid;Ethyl propyl disulfide;Ethyl isopropyl disulfide;2-Hydroxypurine;2,3,4-Trihydroxybutanoic acid;9H-Purine-9-ol;N-methylethanolamine phosphate | 0.60 | down | 0.04 | 0.57 | 1.45 |
| NEG | NEG_193.035_0.5957_1 | Glucuronic acid | C6H10O7 | Organic oxygen compounds | Organooxygen compounds | Carbohydrates and carbohydrate conjugates | HMDB0000127 | C00191 | map00040(Pentose and glucuronate interconversions);map00053(Ascorbate and aldarate metabolism);map00520(Amino sugar and nucleotide sugar metabolism);map00562(Inositol phosphate metabolism);map01100(Metabolic pathways);map01240(Biosynthesis of cofactors);map01250(Biosynthesis of nucleotide sugars) | Galacturonic acid;Iduronic acid;Pectic acid;Pectin;3-Dehydro-L-gulonate;5-Keto-D-gluconate;2-Keto-L-gluconate;6-(Hydroxymethyl)-2,4(1H,3H)-pteridinedione;L-Altruronic acid;Aldehydo-L-iduronate;(2R,3R,4R,5S,6S)-3,4,5,6-Tetrahydroxyoxane-2-carboxylic acid;Lactate oxygen;theophylline one;Mannuronic acid;Citramalic acid;3-Hydroxyglutaric acid;D-2-Hydroxyglutaric acid;L-2-Hydroxyglutaric acid;Ribonolactone;D-Xylono-1,5-lactone;2-Propenyl propyl disulfide;Methyl 3-methyl-1-butenyl disulfide;1-Propenyl propyl disulfide;3-methylmalate(2-);monoacetone;Poly(lactic acid-co-glycolic acid);D-Arabinono-1,4-lactone;trans-Propenyl propyl disulfide;Malic acid;D-Malic acid;Velcorin;3,3-Dimethyl-1,2-dithiolane;Tetrahydro-2-methyl-2-thiophenethiol;Tetrahydro-2-methyl-3-thiophenethiol;Diglycolic acid;Pyrazolopyrimidinone;2-Carboxyoxypropanoic acid;1,2-Dimercaptocyclopentane | 0.53 | down | 0.02 | 0.56 | 1.66 |
| NEG | NEG_721.53_6.3941_1 | Cer(d18:0/LTE4) | C41H74N2O6S | Lipids and lipid-like molecules | Sphingolipids | Ceramides | HMDB0289958 | C12126 | map00600(Sphingolipid metabolism);map01100(Metabolic pathways);map04071(Sphingolipid signaling pathway) | DG(18:0/20:4(6Z,8E,10E,14Z)-2OH(5S,12R)/0:0);DG(20:4(6Z,8E,10E,14Z)-2OH(5S,12R)/18:0/0:0);DG(18:0/0:0/20:4(6Z,8E,10E,14Z)-2OH(5S,12R));DG(20:4(6Z,8E,10E,14Z)-2OH(5S,12R)/0:0/18:0);DG(18:0/20:4(6E,8Z,11Z,13E)-2OH(5S,15S)/0:0);DG(20:4(6E,8Z,11Z,13E)-2OH(5S,15S)/18:0/0:0);DG(18:0/0:0/20:4(6E,8Z,11Z,13E)-2OH(5S,15S));DG(20:4(6E,8Z,11Z,13E)-2OH(5S,15S)/0:0/18:0);DG(18:0/20:4(8Z,11Z,14Z,17Z)-2OH(5S,6R)/0:0);DG(20:4(8Z,11Z,14Z,17Z)-2OH(5S,6R)/18:0/0:0);DG(18:0/0:0/20:4(8Z,11Z,14Z,17Z)-2OH(5S,6R));DG(20:4(8Z,11Z,14Z,17Z)-2OH(5S,6R)/0:0/18:0);DG(i-18:0/20:4(6Z,8E,10E,14Z)-2OH(5S,12R)/0:0);DG(20:4(6Z,8E,10E,14Z)-2OH(5S,12R)/i-18:0/0:0);DG(i-18:0/0:0/20:4(6Z,8E,10E,14Z)-2OH(5S,12R));DG(20:4(6Z,8E,10E,14Z)-2OH(5S,12R)/0:0/i-18:0);DG(i-18:0/20:4(6E,8Z,11Z,13E)-2OH(5S,15S)/0:0);DG(20:4(6E,8Z,11Z,13E)-2OH(5S,15S)/i-18:0/0:0);DG(i-18:0/0:0/20:4(6E,8Z,11Z,13E)-2OH(5S,15S));DG(20:4(6E,8Z,11Z,13E)-2OH(5S,15S)/0:0/i-18:0);DG(i-18:0/20:4(8Z,11Z,14Z,17Z)-2OH(5S,6R)/0:0);DG(20:4(8Z,11Z,14Z,17Z)-2OH(5S,6R)/i-18:0/0:0);DG(i-18:0/0:0/20:4(8Z,11Z,14Z,17Z)-2OH(5S,6R));DG(i-18:0/22:6(5Z,7Z,10Z,13Z,16Z,19Z)-OH(4)/0:0);DG(17:0/20:4(6Z,8E,10E,14Z)-2OH(5S,12R)/0:0);DG(20:4(6Z,8E,10E,14Z)-2OH(5S,12R)/17:0/0:0);DG(17:0/0:0/20:4(6Z,8E,10E,14Z)-2OH(5S,12R));DG(20:4(6Z,8E,10E,14Z)-2OH(5S,12R)/0:0/17:0);DG(17:0/20:4(6E,8Z,11Z,13E)-2OH(5S,15S)/0:0);DG(20:4(6E,8Z,11Z,13E)-2OH(5S,15S)/17:0/0:0);DG(17:0/0:0/20:4(6E,8Z,11Z,13E)-2OH(5S,15S));DG(20:4(6E,8Z,11Z,13E)-2OH(5S,15S)/0:0/17:0);DG(17:0/20:4(8Z,11Z,14Z,17Z)-2OH(5S,6R)/0:0);DG(20:4(8Z,11Z,14Z,17Z)-2OH(5S,6R)/17:0/0:0);DG(17:0/0:0/20:4(8Z,11Z,14Z,17Z)-2OH(5S,6R));DG(20:4(8Z,11Z,14Z,17Z)-2OH(5S,6R)/0:0/17:0);DG(a-17:0/20:4(6Z,8E,10E,14Z)-2OH(5S,12R)/0:0);DG(20:4(6Z,8E,10E,14Z)-2OH(5S,12R)/a-17:0/0:0);DG(a-17:0/0:0/20:4(6Z,8E,10E,14Z)-2OH(5S,12R));DG(20:4(6Z,8E,10E,14Z)-2OH(5S,12R)/0:0/a-17:0);DG(a-17:0/20:4(6E,8Z,11Z,13E)-2OH(5S,15S)/0:0);DG(20:4(6E,8Z,11Z,13E)-2OH(5S,15S)/a-17:0/0:0);DG(a-17:0/0:0/20:4(6E,8Z,11Z,13E)-2OH(5S,15S));DG(20:4(6E,8Z,11Z,13E)-2OH(5S,15S)/0:0/a-17:0);DG(a-17:0/20:4(8Z,11Z,14Z,17Z)-2OH(5S,6R)/0:0);DG(20:4(8Z,11Z,14Z,17Z)-2OH(5S,6R)/a-17:0/0:0);DG(a-17:0/0:0/20:4(8Z,11Z,14Z,17Z)-2OH(5S,6R));DG(20:4(8Z,11Z,14Z,17Z)-2OH(5S,6R)/0:0/a-17:0);DG(i-17:0/20:4(6Z,8E,10E,14Z)-2OH(5S,12R)/0:0);DG(20:4(6Z,8E,10E,14Z)-2OH(5S,12R)/i-17:0/0:0);DG(i-17:0/0:0/20:4(6Z,8E,10E,14Z)-2OH(5S,12R));DG(20:4(6Z,8E,10E,14Z)-2OH(5S,12R)/0:0/i-17:0);DG(i-17:0/20:4(6E,8Z,11Z,13E)-2OH(5S,15S)/0:0);DG(20:4(6E,8Z,11Z,13E)-2OH(5S,15S)/i-17:0/0:0);DG(i-17:0/0:0/20:4(6E,8Z,11Z,13E)-2OH(5S,15S));DG(20:4(6E,8Z,11Z,13E)-2OH(5S,15S)/0:0/i-17:0);DG(i-17:0/20:4(8Z,11Z,14Z,17Z)-2OH(5S,6R)/0:0);DG(20:4(8Z,11Z,14Z,17Z)-2OH(5S,6R)/i-17:0/0:0);DG(i-17:0/0:0/20:4(8Z,11Z,14Z,17Z)-2OH(5S,6R));DG(20:4(8Z,11Z,14Z,17Z)-2OH(5S,6R)/0:0/i-17:0) | 1.82 | up | 0.05 | 0.57 | 1.43 |
| NEG | NEG_135.031_1.0794_1 | Threonic acid | C4H8O5 | Organic oxygen compounds | Organooxygen compounds | Carbohydrates and carbohydrate conjugates | HMDB0000943 | C01620 | map00053(Ascorbate and aldarate metabolism);map01100(Metabolic pathways) | Hypoxanthine;Erythronic acid;Threonic acid;Allopurinol;1-Pentanesulfenothioic acid;Ethyl propyl disulfide;Ethyl isopropyl disulfide;2-Hydroxypurine;2,3,4-Trihydroxybutanoic acid;9H-Purine-9-ol;L-Lactic acid;Glyceraldehyde;Dihydroxyacetone;Dimethyl carbonate;Monoethyl carbonate;Methoxyacetic acid;1,2,4-Trioxane;1,3,5-Trioxane;Methyl ethaneperoxoate;Peracetic acid;1,2,3-Trioxolane;Monomethyl carbonate | 0.54 | down | 0.02 | 0.56 | 1.57 |
| NEG | NEG_476.279_4.6413_1 | LysoPE(18:2(9Z,12Z)/0:0) | C23H44NO7P | Lipids and lipid-like molecules | Glycerophospholipids | Glycerophosphoethanolamines | HMDB0011507 | C04438 | map00564(Glycerophospholipid metabolism) | LysoPE(0:0/18:2(9Z,12Z));LysoPE(18:2(9Z,12Z)/0:0);VAPIPROST;Aglepristone | 0.26 | down | 0.02 | 0.56 | 1.66 |
| POS | POS_332.11_1.132_1 | Citbrasine | C17H17NO6 | Organoheterocyclic compounds | Quinolines and derivatives | Benzoquinolines | HMDB0037797 | NA | NA | R-95913;Citbrasine;N-(1-Deoxy-1-fructosyl)leucine;N-(1-Deoxy-1-fructosyl)isoleucine;3,4,5-Trihydroxypentanoylcarnitine;Ketotifen;4',7-Dihydroxy-2',5-dimethoxyisoflavone;Gnaphaliin;5-Hydroxy-7,8-dimethoxyflavonol;4',5-Dihydroxy-7,8-dimethoxyflavone;3,4-Dihydroxycinnamoyl-(Z)-2-(3,4-dihydroxyphenyl)ethenol;Muscomosin;Pisatin;4',5-Dihydroxy-3',7-dimethoxyisoflavone;Aflatoxin B2;Sylpin;2',5-Dihydroxy-4',7-dimethoxyisoflavone;3',7-Dihydroxy-4',8-dimethoxyisoflavone;2',7-Dihydroxy-4',5'-dimethoxyisoflavone;3-O-Acetylpinobanksin;(3S,7R)-16-Hydroxy-11-methoxy-6,8,19-trioxapentacyclo[10.7.0.02,9.03,7.013,17]nonadeca-1,4,9,11,13(17)-pentaen-18-one;Cirsimaritin;5,7-Dihydroxy-3,6-dimethoxyflavone | 0.42 | down | 0.00 | 0.48 | 2.47 |
| POS | POS_410.29_3.5761_1 | 7-(3,4-Dimethyl-5-propylfuran-2-yl)heptanoylcarnitine | C23H39NO5 | Lipids and lipid-like molecules | Fatty Acyls | Fatty acid esters | HMDB0241849 | NA | NA | 5-(3,4-Dimethyl-5-pentylfuran-2-yl)pentanoylcarnitine;6-(5-Hexylfuran-2-yl)hexanoylcarnitine;7-(3,4-Dimethyl-5-propylfuran-2-yl)heptanoylcarnitine;7-(5-Pentylfuran-2-yl)heptanoylcarnitine;8-(5-Butylfuran-2-yl)octanoylcarnitine;N-Linoleoyl Glutamic acid;2-Hydroxymyristoylcarnitine;3-Hydroxytetradecanoyl carnitine;20, 22-Dihydrodigoxigenin;VIPROSTOL;MG(20:4(6E,8Z,11Z,14Z)+=O(5)/0:0/0:0);MG(20:4(5Z,8Z,11Z,13E)+=O(15)/0:0/0:0);MG(20:5(5Z,8Z,11Z,14Z,16E)-OH(18R)/0:0/0:0);MG(20:5(5Z,8Z,11Z,14Z,16E)-OH(18)/0:0/0:0);MG(20:5(5Z,8Z,10E,14Z,17Z)-OH(12)/0:0/0:0);MG(20:5(6E,8Z,11Z,14Z,17Z)-OH(5)/0:0/0:0);MG(0:0/20:4(6E,8Z,11Z,14Z)+=O(5)/0:0);MG(0:0/20:4(5Z,8Z,11Z,13E)+=O(15)/0:0);MG(0:0/20:5(5Z,8Z,11Z,14Z,16E)-OH(18R)/0:0);MG(0:0/20:5(5Z,8Z,11Z,14Z,16E)-OH(18)/0:0);MG(0:0/20:5(5Z,8Z,10E,14Z,17Z)-OH(12)/0:0);MG(0:0/20:5(6E,8Z,11Z,14Z,17Z)-OH(5)/0:0) | 1.67 | up | 0.05 | 0.57 | 1.43 |
| POS | POS_934.643_8.869_1 | DGDG 34:2 | C49H88O15 | Lipids and lipid-like molecules | Glycerolipids | Glycosylglycerols | NA | C06037 | map00561(Glycerolipid metabolism);map01100(Metabolic pathways) | Glycerol 2-(9Z,12Z-octadecadienoate) 1-hexadecanoate 3-O-[alpha-D-galactopyranosyl-(1->6)-beta-D-galactopyranoside];Digalactosyldiacylglycerol | 1.95 | up | 0.00 | 0.52 | 1.53 |
| POS | POS_401.343_8.1482_1 | Calcifediol | C27H44O2 | Lipids and lipid-like molecules | Steroids and steroid derivatives | Vitamin D and derivatives | NA | C01561 | map00100(Steroid biosynthesis);map01100(Metabolic pathways);map01110(Biosynthesis of secondary metabolites);map04928(Parathyroid hormone synthesis, secretion and action);map05152(Tuberculosis) | 7-Ketocholesterol;7a-Hydroxy-cholestene-3-one;Calcidiol;5,6-trans-25-Hydroxyvitamin D3;25-Hydroxytachysterol3;Alfacalcidol;(3beta,5alpha,6a)-Cholesta-8,14-diene-3,6-diol;1-Phenyl-1,3-heneicosanedione;5-Methyl-2,4-bis(3-methyl-2-butenyl)-6-(2-methyl-1-oxopropyl)-5-(4-methyl-3-pentenyl)cyclohexanone;5-(12,15-Heneicosadienyl)-1,3-benzenediol;3beta-Hydroxy-5-cholestenal;27alpha-Hydroxy-8-dehydrocholesterol;(1R,3Z)-3-[(2E)-2-[(1R,3As,7aR)-1-[(2R)-7-hydroxy-6-methylheptan-2-yl]-7a-methyl-2,3,3a,5,6,7-hexahydro-1H-inden-4-ylidene]ethylidene]-4-methylidenecyclohexan-1-ol;(1R)-3-[2-[(1R,3Ar,7aR)-1-[(2R)-6-hydroxy-6-methylheptan-2-yl]-7a-methyl-2,3,3a,5,6,7-hexahydro-1H-inden-4-ylidene]ethylidene]-4-methylidenecyclohexan-1-ol;(1R)-5-[2-[(1R,7Ar)-7a-methyl-1-[(2R)-6-methylheptan-2-yl]-2,3,3a,5,6,7-hexahydro-1H-inden-4-ylidene]ethylidene]-4-methylidenecyclohexane-1,3-diol;(20S)-Hydroxyvitamin D3;22-Oxocholesterol;24,25-Epoxycholesterol;3-Hydroxycholest-5-en-7-one;3-Hydroxy-10,13-dimethyl-17-(6-methylheptan-2-yl)-1,2,3,4,5,6,7,9,11,12,16,17-dodecahydrocyclopenta[a]phenanthren-15-one;Cholestane-2,3-dione;Gefarnate;hydroxyvitamin d3;ketocholesterol;Sapogenins;Erucoylacetone | 1.67 | up | 0.05 | 0.57 | 1.16 |
| NEG | NEG_558.46_6.5407_1 | Cer-AS d35:5 | C35H61NO4 | Lipids and lipid-like molecules | Sphingolipids | Ceramides | NA | NA | NA | NA | 1.70 | up | 0.03 | 0.56 | 1.21 |
| NEG | NEG_171.007_0.8994_1 | Glycerol 2-phosphate | C3H9O6P | Lipids and lipid-like molecules | Glycerophospholipids | Glycerophosphates | NA | C02979 | NA | Glycerol 3-phosphate;beta-Glycerophosphoric acid;Glycerophosphoric acid;Hypoxanthine;Erythronic acid;Threonic acid;Allopurinol;1-Pentanesulfenothioic acid;Ethyl propyl disulfide;Ethyl isopropyl disulfide;2-Hydroxypurine;2,3,4-Trihydroxybutanoic acid;9H-Purine-9-ol;Ethylphosphate;Dimethylphosphate;Methylphosphate;Potassium propionate | 0.37 | down | 0.00 | 0.49 | 2.09 |
| POS | POS_515.243_4.513_1 | PA(2:0/20:4(5Z,8Z,11Z,13E)+=O(15)) | C25H39O9P | Lipids and lipid-like molecules | Glycerophospholipids | Glycerophosphates | HMDB0266533 | C00416 | map00561(Glycerolipid metabolism);map00564(Glycerophospholipid metabolism);map01100(Metabolic pathways);map01110(Biosynthesis of secondary metabolites);map04024(cAMP signaling pathway);map04070(Phosphatidylinositol signaling system);map04072(Phospholipase D signaling pathway);map04666(Fc gamma R-mediated phagocytosis);map04912(GnRH signaling pathway);map04975(Fat digestion and absorption);map05200(Pathways in cancer);map05212(Pancreatic cancer);map05231(Choline metabolism in cancer) | Carmofur;Telmisartan;PA(2:0/20:4(6E,8Z,11Z,14Z)+=O(5));PA(20:4(6E,8Z,11Z,14Z)+=O(5)/2:0);PA(2:0/20:4(5Z,8Z,11Z,13E)+=O(15));PA(20:4(5Z,8Z,11Z,13E)+=O(15)/2:0);PA(2:0/20:5(5Z,8Z,11Z,14Z,16E)-OH(18R));PA(20:5(5Z,8Z,11Z,14Z,16E)-OH(18R)/2:0);PA(2:0/20:5(5Z,8Z,11Z,14Z,16E)-OH(18));PA(20:5(5Z,8Z,11Z,14Z,16E)-OH(18)/2:0);PA(2:0/20:5(5Z,8Z,10E,14Z,17Z)-OH(12));PA(20:5(5Z,8Z,10E,14Z,17Z)-OH(12)/2:0);PA(2:0/20:5(6E,8Z,11Z,14Z,17Z)-OH(5));PA(20:5(6E,8Z,11Z,14Z,17Z)-OH(5)/2:0);Lucidenic acid G;Lucidenic acid H;Lucidenic acid C;DG(2:0/22:6(5Z,8E,10Z,13Z,15E,19Z)-2OH(7S, 17S)/0:0);DG(22:6(5Z,8E,10Z,13Z,15E,19Z)-2OH(7S, 17S)/2:0/0:0);DG(2:0/0:0/22:6(5Z,8E,10Z,13Z,15E,19Z)-2OH(7S, 17S));DG(22:6(5Z,8E,10Z,13Z,15E,19Z)-2OH(7S, 17S)/0:0/2:0);DG(2:0/22:6(4Z,7Z,11E,13Z,15E,19Z)-2OH(10S,17)/0:0);DG(22:6(4Z,7Z,11E,13Z,15E,19Z)-2OH(10S,17)/2:0/0:0);DG(2:0/0:0/22:6(4Z,7Z,11E,13Z,15E,19Z)-2OH(10S,17));DG(22:6(4Z,7Z,11E,13Z,15E,19Z)-2OH(10S,17)/0:0/2:0);Bezitramide;Lurasidone;PA(2:0/18:1(12Z)-O(9S,10R));PA(18:1(12Z)-O(9S,10R)/2:0);PA(2:0/18:1(9Z)-O(12,13));PA(18:1(9Z)-O(12,13)/2:0);Validamycin A | 0.23 | down | 0.05 | 0.57 | 2.36 |
| NEG | NEG_560.477_7.0357_1 | Cer 15:3;2O/20:1;(2OH) | C35H63NO4 | Lipids and lipid-like molecules | Sphingolipids | Ceramides | NA | NA | NA | NA | 1.76 | up | 0.04 | 0.56 | 1.27 |
| POS | POS_556.529_8.0788_1 | Cer(t18:0/16:0) | C34H69NO4 | Lipids and lipid-like molecules | Sphingolipids | Ceramides | HMDB0010697 | C12126 | map00600(Sphingolipid metabolism);map01100(Metabolic pathways);map04071(Sphingolipid signaling pathway) | Cer(t18:0/16:0);Armillaramide;FAHFA(16:0/9-O-18:0);FAHFA(16:0/5-O-18:0);FAHFA(16:0/10-O-18:0);FAHFA(16:0/12-O-18:0);FAHFA(16:0/7-O-18:0);FAHFA(16:0/8-O-18:0);FAHFA(16:0/11-O-18:0);FAHFA(16:0/13-O-18:0);FAHFA(18:0/5-O-16:0);FAHFA(18:0/7-O-16:0);FAHFA(18:0/8-O-16:0);FAHFA(18:0/9-O-16:0);FAHFA(18:0/10-O-16:0);FAHFA(18:0/11-O-16:0);FAHFA(18:0/12-O-16:0);FAHFA(18:0/13-O-16:0);FAHFA(16:0/6-O-18:0);FAHFA(18:0/6-O-16:0) | 1.55 | up | 0.03 | 0.56 | 1.47 |
| POS | POS_283.025_1.9271_1 | Scutellarein | C15H10O6 | Phenylpropanoids and polyketides | Flavonoids | Flavones | NA | C10184 | NA | Calcium benzoate;N-Palmitoyl Asparagine | 0.17 | down | 0.01 | 0.52 | 2.91 |
| POS | POS_515.243_5.4275_1 | Asn Gly Pro Val Glu | C21H34N6O9 | NA | NA | NA | NA | NA | NA | Carmofur;Telmisartan;PA(2:0/20:4(6E,8Z,11Z,14Z)+=O(5));PA(20:4(6E,8Z,11Z,14Z)+=O(5)/2:0);PA(2:0/20:4(5Z,8Z,11Z,13E)+=O(15));PA(20:4(5Z,8Z,11Z,13E)+=O(15)/2:0);PA(2:0/20:5(5Z,8Z,11Z,14Z,16E)-OH(18R));PA(20:5(5Z,8Z,11Z,14Z,16E)-OH(18R)/2:0);PA(2:0/20:5(5Z,8Z,11Z,14Z,16E)-OH(18));PA(20:5(5Z,8Z,11Z,14Z,16E)-OH(18)/2:0);PA(2:0/20:5(5Z,8Z,10E,14Z,17Z)-OH(12));PA(20:5(5Z,8Z,10E,14Z,17Z)-OH(12)/2:0);PA(2:0/20:5(6E,8Z,11Z,14Z,17Z)-OH(5));PA(20:5(6E,8Z,11Z,14Z,17Z)-OH(5)/2:0);Lucidenic acid G;Lucidenic acid H;Lucidenic acid C;DG(2:0/22:6(5Z,8E,10Z,13Z,15E,19Z)-2OH(7S, 17S)/0:0);DG(22:6(5Z,8E,10Z,13Z,15E,19Z)-2OH(7S, 17S)/2:0/0:0);DG(2:0/0:0/22:6(5Z,8E,10Z,13Z,15E,19Z)-2OH(7S, 17S));DG(22:6(5Z,8E,10Z,13Z,15E,19Z)-2OH(7S, 17S)/0:0/2:0);DG(2:0/22:6(4Z,7Z,11E,13Z,15E,19Z)-2OH(10S,17)/0:0);DG(22:6(4Z,7Z,11E,13Z,15E,19Z)-2OH(10S,17)/2:0/0:0);DG(2:0/0:0/22:6(4Z,7Z,11E,13Z,15E,19Z)-2OH(10S,17));DG(22:6(4Z,7Z,11E,13Z,15E,19Z)-2OH(10S,17)/0:0/2:0);Bezitramide;Lurasidone;PA(2:0/18:1(12Z)-O(9S,10R));PA(18:1(12Z)-O(9S,10R)/2:0);PA(2:0/18:1(9Z)-O(12,13));PA(18:1(9Z)-O(12,13)/2:0);Validamycin A | 0.32 | down | 0.02 | 0.55 | 2.56 |
| NEG | NEG_155.002_0.6155_1 | Orotic acid | C5H4N2O4 | Organoheterocyclic compounds | Diazines | Pyrimidines and pyrimidine derivatives | HMDB0000226 | C00295 | map00240(Pyrimidine metabolism);map01100(Metabolic pathways);map01240(Biosynthesis of cofactors) | Benzofurazan;Oxazolo[5,4-B]pyridine;Ethanesulfonic acid;Methyl methanesulfonate;Mesylate | 0.33 | down | 0.04 | 0.57 | 1.52 |
| POS | POS_167.115_0.6144_1 | 3-Isopropyl-2-methoxy-5-methylpyrazine | C9H14N2O | Organoheterocyclic compounds | Diazines | Pyrazines | HMDB0029742 | NA | NA | N-Acetylcadaverine;1-(3-Aminopropyl)-4-aminobutanal;4-(Aminomethyl)-1-methylpiperidin-4-ol;Milacemide | 2.08 | up | 0.03 | 0.56 | 1.80 |
